# Changes in Adolescents’ COVID-19-Health-Related Stress, Parent-Adolescent Relationships, and Mental Health During the COVID-19 Pandemic: The Effect of Personality Traits

**DOI:** 10.1007/s10964-024-02048-w

**Published:** 2024-07-23

**Authors:** Monika H. Donker, Stefanos Mastrotheodoros, Takuya Yanagida, Susan Branje

**Affiliations:** 1 Department of Education and Pedagogy, Section Youth and Family, Utrecht University, Utrecht, The Netherlands; 2 Department of Psychology, University of Crete, Rethymno, Greece; 3 Department of Developmental and Educational Psychology, Faculty of Psychology, University of Vienna, Vienna, Austria

**Keywords:** COVID-19, Mental health, Parent-adolescent relationship, Personality, Stress

## Abstract

Previous studies investigated short-term effects of COVID-19 on families. However, much is unknown about how families with adolescents fared throughout the pandemic, as well as factors that might explain interindividual differences in adjustment. The current study used latent change score models to investigate associations between changes in adolescents’ mental health, parent-adolescent relationship quality, and COVID-19-health-related stress from Fall 2019 to Spring 2021, and whether personality predicted changes in adolescents’ mental health, relationship quality, and stress. Participants were 242 adolescents (M_age_ = 11.56, SD = 0.44, 50% girls). Parent-adolescent negative interactions decreased from before the pandemic to the first lockdown, and stronger decreases (both in this period and between Fall 2020 and Spring 2021) were associated with simultaneous stronger increases in mental health. From Spring to Fall 2020, decreases in stress were stronger for less extraverted adolescents and were associated with better mental health. More agreeable adolescents reported a stronger decrease in stress between Fall 2020 and Spring 2021. The findings suggest that it is important to consider heterogeneity in designing future intervention and prevention programs. Especially adolescents with existing problems and from multi-problem families might be at risk for adverse consequences during pandemic-like situations.

## Introduction

**Fig. 1:**
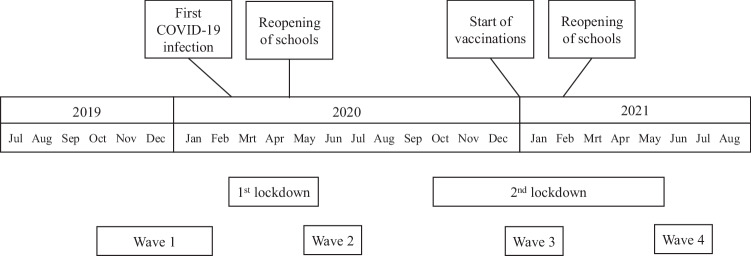
Overview of the main COVID-19-related events in the Netherlands and the timing of the data collection waves

In 2020 and 2021, the coronavirus disease 2019 (COVID-19) pandemic and the preventive measures to control the spread of the virus have had a major impact on the daily life of families. Many studies have investigated short-term effects of the pandemic on anxiety, depression, and stress in the general population (e.g., Wang et al., 2020) as well as on parental stress and mental health (Brown et al., 2020) and adolescent mental health and well-being (Keijsers & Bülow, 2020) more specifically. However, much is unclear about the long-term impact of the pandemic and its preventive measures on stress, parent-child relationship quality, and mental health. Additionally, to inform future decisions, it seems relevant to investigate interindividual differences in resilience to the effects of the pandemic. The current study therefore investigated changes in stress, parent-adolescent relationship quality, and adolescent mental health at four timepoints across a time span of 1.5 years (i.e., Fall 2019, Spring 2020, Fall 2020, Spring 2021; see also Fig. 1), and the effects of pre-pandemic personality factors on these changes.

### COVID-19-Related Changes in Mental Health Problems

Much attention has been paid to the impact of the pandemic on the mental health of people in general (Aknin et al., 2022; Santomauro et al., 2021), and adolescents in particular. A meta-analysis suggests that about 25% of adolescents showed symptoms of depression and 20% showed anxiety symptoms during the pandemic, which is twice as much as before the pandemic (Racine et al., 2021). A systematic review even reported anxiety and depression symptoms in up to 50 or 60% of children and adolescents (aged ≤ 19 years) during the pandemic (Panchal et al., 2021). In the Netherlands, over 90% of children and adolescents reported a negative impact of COVID-19 on their daily life (Luijten et al., 2021).

Previous studies have associated especially quarantine with poorer mental health (Brooks et al., 2020), possibly due to the limited possibilities for interaction with peers. As in many other countries, children in the Netherlands could not attend school and engaged in home schooling for several periods, had limited (face-to-face) contact with peers, and their structured leisure activities were often canceled. It has been suggested that especially these governmental restrictions, rather than the virus itself, have impacted adolescents (Magson et al., 2021).

There is some evidence that the negative effects of the pandemic on mental health increased over time in the first year of the pandemic (Racine et al., 2021). Furthermore, older children (i.e., 13–15 years compared to 6–12 years) and females were especially at risk (Racine et al., 2021). Also, children with special needs and existing mental problems before the pandemic had a heightened risk to experience mental health problems during the COVID-19 period (Panchal et al., 2021).

### COVID-19-Related Changes in Parent-Adolescent Relationships

The social distancing restrictions required changes in family life, with implications for parent-child relationship quality and parenting strategies and behavior (Weeland et al., 2021). Especially during the lockdown periods, many parents had to work from home, while at the same time home-schooling their children (Shockley et al., 2020). In general, caregivers seemed to have increased authoritarian parenting behaviors, such as fear induction practices (Ren et al., 2021), decreased autonomy support (although this decrease seemed temporary; Bülow et al., 2021), and became more knowledgeable of their children’s behaviors and everyday activities (Cassinat et al., 2021). These changes might have impacted the relationships between parents and children. For example, decreases in support and positive parenting, but also in negative interactions during the first period of the pandemic were found (Donker et al., 2021). Also others reported both positive (e.g., re-discovering family) and negative (e.g., limitation of autonomy) consequences for family life during the first months of the pandemic in Italy (Fioretti et al., 2020). It is likely that in the beginning of the pandemic, parents and adolescents were motivated to make the best out of the situation and enjoyed the lack of strict schedules and planned events. It is however unclear how these effects might change later in the pandemic, as parents and adolescents might get tired of staying at home together for a long time, and the effects of social distancing on adolescents’ autonomy development might become more profound (Harms & Record, 2023).

### Changes in COVID-19-Health-Related Stress

An important reason for examining changes in mental health problems and relationship quality during different phases of the pandemic is that levels of COVID-19-related stress are likely to have varied across these different phases. Stress resulting from the pandemic could be related to various aspects, including the adaptation to new roles and routines (Brock & Laifer, 2020), job loss and socioeconomic costs or worries related to infection of yourself or close family members (Taylor et al., 2020). For adolescents, especially the lack of social contacts might have caused stress (Ellis et al., 2020). Levels of stress due to lack of social contacts might have varied considerably across different periods of the pandemic in which different restrictive measures were taken. It might be expected that adolescents’ stress was largest in the early phases of the pandemic when the uncertainty about the course of the pandemic was highest and the lockdown measures limited their face-to-face contacts with peers and friends (Zacher & Rudolph, 2021). Research on full-time employees in Germany (M_age_ 45.0 years) reported declines in perceived stress from April-September 2020 (Zacher & Rudolph, 2021). At the same time, the stress resulting from social restrictions might have increased because of stressor accumulation. Many of the studies so far focused on the pandemic’s first months, yet the prolonged duration of the pandemic could also result in more chronic stress, which might change the impact of the pandemic on adolescent development.

Moreover, although many people experienced COVID-19-related stress because of the huge impact and uncertainty of the situation (e.g., Qiu et al., 2020), youth and families were especially affected by the pandemic (Prime et al., 2020), and they differed largely in the extent of worrying and stress related to their health during the pandemic (Masten & Motti-Stefanidi, 2020). The negative impact of the pandemic on health, finances, and social relations was often higher for those families who were already more at risk before the pandemic (Branje & Morris, 2021), because of individual characteristics such as ineffective coping or emotion regulation strategies, relational factors such as low family or peer support, or contextual factors such as low SES (Mastrotheodoros & Ranta, 2022). Particularly those adolescents who were more vulnerable before the pandemic reported the largest negative changes during the pandemic. For example, adolescents who had specific vulnerabilities such as higher stress, maladaptive coping, or internalizing problems before the pandemic experienced more COVID-19 related concerns during the pandemic (Van Loon et al., 2021).

### Associations between Changes in Health-Related Stress, Parent-Adolescent Relationships, and Mental Health Problems

It is likely that stress, parent-adolescent relationships, and mental health problems reciprocally affect each other during the COVID-19 period (Low & Mounts, 2022). For example, several studies found an association between COVID-19 related stress and self-reported internalizing symptoms (Bernasco et al., 2021), loneliness (Ellis et al., 2020), and life satisfaction (Magson et al., 2021). Furthermore, families with high stress levels might have more difficulty to adapt to the new caregiving situation, and thus experience lower quality parent-adolescent relationships (Brown et al., 2020). On the other hand, many studies describe a buffering effect of a well-functioning family system on adolescent adjustment (Campione-Barr et al., 2021) and stress (Wu et al., 2021). Time spent with the family was for example associated with lower levels of depression and loneliness (Ellis et al., 2020). On the other hand, increased conflict with parents has been associated with increased mental health problems in the beginning of the pandemic (Magson et al., 2021). Thus, strong correlations are expected between changes in health-related stress, parent-adolescent relationship quality, and mental health problems, at least in the early phases of the pandemic.

### The Role of Personality in the Impact of the COVID-19 Pandemic

Although some of the variation in effects of the COVID-19 pandemic have been attributed to variations in health consequences and restrictive measures, individual characteristics might also affect the impact of the pandemic on stress, mental health problems, and parent-adolescent relationships. The Big Five personality factors extraversion, agreeableness, conscientiousness, emotional stability, and openness to experience can be seen as universal among humans (McCrae & Costa, 1997) and relatively stable from age 12 onwards (Borghuis et al., 2017). According to diathesis-stress models, people with different personality characteristics might react differently to similar situations (Monroe & Simons, 1991), which makes it relevant to assess personality in stressful situations, such as the COVID-19 pandemic, to be able to give adequate support to the groups who might need it the most (Zettler et al., 2022). Previous studies have indeed found differential effects of natural disasters on depression for individuals with different personality traits (Kopala-Sibley et al., 2016) and moderating effects of certain character strengths on the association between stress and depression during the COVID-19 pandemic (Liu & Wang, 2021).

In general, a meta-analysis has shown that higher levels of extraversion, agreeableness, conscientiousness, emotional stability, and openness to experience are associated with resilience (Oshio et al., 2018). Similar results are expected also for the COVID-19 pandemic, except for extraversion. Adolescents scoring high on extraversion might be hit extra hard by the contact-restrictive measures. There is some first evidence for these effects during the pandemic. Extraverted adolescents reported more tensions at home (Iterbeke & De Witte, 2021), and more (biological) stress (Engert et al., 2021). Moreover, people scoring high on extraversion reported a larger rise in depressiveness, partly explained by increased loneliness (Alt et al., 2021) and a less strong positive correlation with positive affect during the pandemic (Anglim & Horwood, 2021). Also in an adult sample, participants scoring higher on extraversion experienced larger increases in stress during the beginning of the pandemic (Zacher & Rudolph, 2021), but not in the later phases of the pandemic (Bellingtier et al., 2021).

For the other personality traits, there is indeed some evidence for a protective role in the effects of the COVID-19 pandemic. Emotional stability was associated with lower perceived stress in general (Bellingtier et al., 2021) as well as specifically related to school closure (Iterbeke & De Witte, 2021), less biological stress (Engert et al., 2021), less loneliness, and higher well-being (Gubler et al., 2021). Moreover, less emotionally stable adults reported less adaptive psychological functioning, both directly and indirectly via diminished resilience (Zager Kocjan et al., 2021). People high in conscientiousness and agreeableness better adhered to the social distancing guidelines (Moore et al., 2022), and showed adaptive effects, such as a better perception of remote learning (Iterbeke & De Witte, 2021) and helping others during the first wave (Kohút et al., 2021), respectively. Openness to experience has been linked to seeing the COVID-19 period as an opportunity to learn new skills (Iterbeke & De Witte, 2021).

However, most of these studies were conducted in the early phases of the pandemic, and it is still unknown whether the effects will change with the longer duration of the pandemic. For example, it has been found that the effect of personality traits was lower in the second wave (September 2020; Kohút et al., 2021), possibly because people experienced more threat and negative affect during the first wave, leading to amplified effects of personality (Bedford-Petersen & Saucier, 2021).

## Current Study

To get a better understanding of the long-term effects of the COVID-19 pandemic on adolescents, this study investigated changes in adolescents’ COVID-19-health-related stress, relationship quality with parents, and mental health at four timepoints across a time span of 1.5 years (i.e., Fall 2019, Spring 2020, Fall 2020, Spring 2021; see also Fig. 1). Moreover, to get grip on potential interindividual differences in resilience, the effect of pre-pandemic personality traits on these changes was assessed. The research questions and hypotheses were preregistered at 10.17605/OSF.IO/J9SCW. In general, the impact of COVID-19 on adolescents was expected to vary over the course of the pandemic, based on the strictness of the restrictive measures and the future perspective (see Fig. 1). From Spring 2020 to Fall 2020, an increase in COVID-19 health-related stress was hypothesized, as people were worried about the future with the start of the second lockdown. From Fall 2020 to Spring 2021, a decrease in stress was hypothesized, as the vaccination campaign started and restrictive measures were released. For parental support, an increase from before the pandemic to Spring 2020, but a decrease afterwards were expected, as parents get tired from combining working from home and caring for their children. For negative interaction, a decline from Fall 2019 to Spring 2020, and an increase thereafter were expected, with the longer duration of the pandemic. For adolescent mental health problems, an increase over time was expected, and the strongest increase especially during the later waves, because the long-term duration of the pandemic and the restrictive measures might take their toll. Although it was not explicitly mentioned in the pre-registration, significant concurrent associations between changes in relationship quality, stress, and mental health were also expected. Personality was hypothesized to affect the changes in health-related stress, parent-adolescent relationships, and mental health. It is known from previous research (before COVID-19) that higher levels of extraversion, openness, agreeableness, conscientiousness, and emotional stability are associated with resilience and adaptation. Mainly similar results for the COVID-19 pandemic were hypothesized, except for extraversion, as individuals scoring high on extraversion might be hit extra hard by the contact-restrictive measures during the pandemic. Thus, mainly beneficial effects were expected for adolescents scoring higher on openness, agreeableness, conscientiousness, and emotional stability, but worse effects for more extraverted individuals.

## Methods

### Participants

Participants were 244 adolescents (*M*_*age*_ = 11.56, SD = 0.44, 50% girls, at Wave 1) who were followed for four waves across 1.5 years, starting in Fall 2019 until Spring 2021. At the start of the study, they were in the final grade of primary school, and they transitioned to the first grade of secondary school in the summer of 2020. Participants were mostly recruited through schools, and participants lived all over the Netherlands. Adolescents identified mostly (*N* = 234, 97%) as Dutch. Most adolescents (81.6%) reported living with both parents, and 12.7% reported that their parents were divorced at measurement Wave 1 (5.7% missing values). The median parent-reported family net monthly income was between 4000 and 4499 euros.

Of the adolescents participating at Wave 1, 173 (72%) still participated at Wave 4. Missingness was examined separately for each wave (to discern it from attrition) using the *naniar* (Tierney et al., 2020) and the *finalfit* (Harrison, 2020) packages in R. Missingness was low and it ranged from 1.2% for personality variables to 5.7% for support at Wave 1, from 2.6% for anxiety and depression to 3.1% for support, negative interaction, and COVID-19-health-related stress at Wave 2, from 1.5% for anxiety and depression to 5.6% for COVID-19-health-related stress at Wave 3, and from 4.4% for anxiety and depression to 7.7% for COVID-19-health-related stress for Wave 4. A Little’s Missing Completely At Random (MCAR) test turned a non-significant value [*χ*^*2*^ (310) = 344, *p* = .089]. Lower levels of support at Wave 1 were associated with attrition across Wave 2 to Wave 4, whereas boys had a higher chance of attrition only at Wave 3. Attrition was not associated with any other variable.

### Procedure

Data were collected as part of the InTransition project. The project followed adolescents around their transition from primary school to secondary school. In the Netherlands, adolescents make this transition around the age of 12 years. The InTransition project was designed to study processes of change in identity and autonomy at different time scales: moment-to-moment behavior during interactions with parents and friends, relational experiences across hours and days, and long-term development. The study design was approved by the faculty ethics board of Utrecht University. In the current study, only the questionnaire data sampled each half year was used. Adolescents and their parents were asked to complete online questionnaires in Fall and Spring in the year before and after the transition.

The first data collection wave started before the COVID-19 pandemic, and thus this timepoint functions as a baseline measure. The other waves took place during the pandemic. In the Netherlands, the first official COVID-19 infection was confirmed on February 27, 2020. General hygiene measures were taken and halfway March the first intelligent lockdown was announced, including closure of daycare and schools. Primary schools reopened by the end of April, after which data-collection for this project continued (Wave 2). In the summer of 2020 also other activities could go through at a 1.5 m distance. By the end of summer, the number of infections increased again, and in October, the second wave of COVID-19 was official, and a new intelligent lockdown period started during which the data for Wave 3 was collected. This second lockdown period officially lasted until June 2021 and included some weeks of curfew in February. During the first half of 2021, however, some perspective emerged again. For example, the vaccination campaign started in January 2021 and schools and daycare could reopen in February. The data of Wave 4 were collected in the spring of 2021. An overview of the main events in the Netherlands as well as the data collection periods (i.e., waves) for the InTransition project is given in Fig. 1.

### Measures

#### Parent-adolescent relationship quality

Parent-adolescent relationship quality was assessed using two subscales of the Dutch translation of the Network of Relationships Inventory (NRI; Furman & Buhrmester, 1985): the Negative Interactions scale (6 items; α = .90–.91 across Waves 1–4) and the Parental Support scale (7 items; α = .84–.87, across Waves 1–4). An example item for Negative Interactions is “How often do you disagree with your parents?” and for Parental Support “How much do your parents help you figure out or fix things?”. There was no explicit reference to a specific period. Adolescents answered the questions on a 5-point Likert scale, ranging from 1 (*never*) to 5 (*very often*). Items for each scale were averaged.

#### Mental health problems

Mental health problems were assessed using two subscales of the Dutch version of the Revised Child Anxiety and Depression Scale (RCADS; Chorpita et al., 2000): the Major Depression (10 items; α = .80–.87 across Waves 1 to 4), and the Generalized Anxiety (7 items, α = .82–.88 across Waves 1–4) subscales. An example item for Major Depression is “I feel sad or empty” and for Generalized Anxiety “I worry about things”. Adolescents were instructed to think about how they felt now or in the past six months. Questions were answered on a 4-point Likert scale, ranging from 1 (*never*) to 4 (*always*). Items of both subscales were averaged, such that higher scores indicated more mental health problems.

#### COVID-19-health-related stress

COVID-19-health-related stress was assessed in Wave 2, 3, and 4 with four items assessing the impact of COVID-19 on the health of oneself or one’s family or friends, made for the needs of this study. In Wave 2, the items were preceded by the prime: “Since the outbreak of the pandemic (COVID-19), how concerned have you been about …”, and adolescents were asked to address the four items on a 5-point Likert scale from 1 (*not at all*) to 5 (*very much*). In Waves 3 and 4, the prime referred to experiences *in the past month*. The final items used in this study are: “…the chance that you get infected”, “… the impact of the corona pandemic on your physical health”, “… the impact of the corona pandemic on your mental/emotional health” (see Preliminary Results for more information). Cronbach’s alpha ranged from α = .75–.85 across Waves 2–4. Items were averaged, and higher scores indicated more COVID-19-health-related stress.

#### Personality

Personality was assessed in the first wave with the Dutch Quick Big Five scale (QBF; Vermulst & Gerris, 2005), which consists of 30 adjectives. Adolescents addressed each item on a 7-point Likert scale from 1 (strongly disagree) to 7 (strongly agree). There was no explicit reference to a specific period. Per scale, the 6 items were averaged: Openness to Experience (α = .63, e.g., “Creative”), Conscientiousness (α = .80, e.g., “Systematic”), Extraversion (α = .79, e.g., “Talkative”), Agreeableness (α = .75, e.g., “Kind”) and Emotional Stability (α = .78, e.g., “Nervous”, reversed).

### Analytic Plan

The following steps were preregistered. First, skewness of the distributions was examined, to decide which estimators to use in the latent variable analyses. Following Kline (2016), if the skew index γ was higher than 3, then the distributions would be considered highly skewed, and Maximum Likelihood with robust standard errors (MLR estimator) would be used to obtain more accurate standard errors. Otherwise, Maximum Likelihood estimation (ML estimator) would be used. Second, missing value analysis was applied to examine the amount and the patterns of missingness for all variables. Finally, item-level measurement invariance was examined across all four waves, for all scales separately. If the scales did not fulfill the requirement for measurement invariance, partial measurement invariance would be tested.

Longitudinal measurement invariance was tested separately for negative interactions, support, depression, anxiety, and COVID-19-health-related stress. In each case, consecutively more stringent models were tested examining configural (items loading on the same factors), metric (equality of factor loadings) and scalar (equality of item intercepts) measurement invariance. In each case, nested models were compared based on changes in the relative fit indices RMSEA, CFI, and TLI.

To answer the research questions, Latent Change Score (LCS) analyses were conducted using the lavaan package (Rosseel, 2012). Latent Change Scores are often regarded as a *framework* of modeling longitudinal data, instead of a specific modeling technique (Grimm et al., 2017). The main characteristic of models under this framework is the focus on latent change between adjacent timepoints, that is, on *time-dependent change*, instead of on observed scores at a specific time-point (Grimm et al., 2017). Such an approach fits the goals of this study, namely testing the associations among changes during the different waves of the pandemic. Specifically, the LCS models in this study were specified such that *latent true scores* were estimated for each time point, and *latent proportional change scores* were estimated between adjacent latent true scores. Therefore, latent change scores were proportional to the latent true scores at the previous time point. Latent change across each of the three time-windows [from T1 to T2 (except for stress), T2 to T3, and T3 to T4] was examined. Fit of the models was evaluated using the Root Mean Squared Error of Approximation [RMSEA; Steiger, 1990], the Standardized Root-Mean Residual [SRMR], the Comparative Fit Index [CFI; Bentler, 1990], and the Tucker-Lewis Index [TLI; Tucker & Lewis, 1973].

To examine whether changes in COVID-health-related stress, parent-adolescent relationships, and mental health throughout the pandemic are related to each other, factor covariances were added to the model above, linking the four latent change factors concurrently across each of the time-windows.

**Fig. 2:**
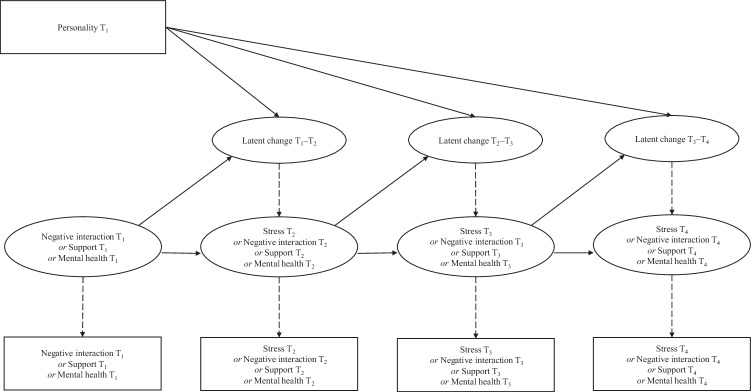
Example path diagram of the univariate Latent Change Score (LCS) model. *Note*. All dashed pathways represent parameters fixed to one

Finally, a model was run where the LCS models for COVID-health-related stress, parent-adolescent relationships, and mental health were regressed on the five personality dimensions. If the model with five predictors would not converge, then the personality dimensions would be used as separate predictors in separate models. Similarly, if model(s) including all LCS analyses at once would not converge, then separate models for each outcome would be ran. In each of the final models, covariates were added, to control for the effects of adolescent gender, living situation, and family SES. See Fig. 2 for a schematic overview of the LCS model.

### Deviations from the Pre-Registered Analytic Plan

During the review process, concerns were raised about the statistical power of the sample size in this study to detect meaningful effects, especially regarding the predictive role of personality. To address these concerns, Bayesian analyses were added as sensitivity analyses to the final models (i.e., those including personality predictors). Compared to frequentist statistics and the standard way to use *p*-values in decision making, Bayesian statistics are less dependent on sample size, and allow for more granular decisions.

In this study, a naïve Bayesian approach was applied, which is based solely on software defaults (Smid et al., 2020). A thoughtful Bayesian approach would require constructing more informative priors, whereby researchers need to find previous studies that used similar models, with measures using the same scales, and controlling for the same variables (see e.g., Smid et al., 2020, p. 147). For this study, such an approach would require the authors to find papers that applied the same type of model (Latent Change Score Models), in roughly the same population (adolescents in the Netherlands, given that COVID-measures differed strongly between countries), in the same type of data (same scale, same control variables). This is impossible given the nature of the study and the conditions of the COVID pandemic.

Bayesian analyses reporting guidelines were followed (Kruschke, 2021), including reporting the Effective Sample Size and Ȓ (or Potential Scale Reduction Factor, PSRF) for each parameter of interest (effects of personality on change scores). Furthermore, to aid interpretation in the Bayesian approach, *probability of direction tests* were applied for these parameters of interest. This test indicates the probability that an effect is larger or smaller than a pre-specified value, and it correlates with the frequentist *p*-value (Makowski et al., 2019). The results of this test are interpreted as the probability of an effect given the data, which is a more useful interpretation compared with the frequentist interpretation of the *p*-value (probability of the data, given no effect) that often leads to a binary decision making.

## Results

### Preliminary Analyses

Regarding the relationship quality indices, scalar longitudinal measurement invariance was supported for negative interactions, but not for support. For support, inspecting the modification indices indicated that the intercepts of four items might be responsible for the large decreases in fit. Releasing the intercepts of these items improved fit but still did not support longitudinal measurement invariance. Thus, partial invariance could not be established, either. Therefore, it was decided to drop support from the analyses. Regarding the mental health problem indices, scalar longitudinal measurement invariance was supported for both depression and anxiety. Furthermore, these two scales correlated highly across waves (*r*’s = .60–.72). Therefore, these two indices were combined into one index of mental health problems. Finally, regarding COVID-19-health-related stress, scalar longitudinal measurement invariance could not be supported. After inspecting the modification indices, it was decided to exclude item 4 (the only item that does not refer to consequences on physical health), and release the equality constraint of the intercept of item 2 between W1 and W4. Therefore, partial longitudinal measurement invariance was supported for COVID-19-health-related stress. No variable had a skew index higher than 3. A Maximum Likelihood (ML) estimator was applied.

### Latent Change Score Models

To answer the research questions, first univariate Latent Change Score models were applied to examine the mean level changes in negative interactions, mental health problems, and COVID-19-health-related stress, separately, and then a multivariate model was run including all three variables, to explore the associations among the change scores. Table 1 presents the means, standard deviations, and bivariate correlations of the main study variables. Table 2 presents the fit indices of all models, and Table 3 presents the estimates of the multivariate Latent Change Score model. On average, the level of negative interactions was low. Negative interactions decreased from before the pandemic (Wave 1) to after the first lockdown period (Wave 2) and remained stable thereafter. The level of mental health problems was low and remained stable, on average, across the pandemic, whereas COVID-19 related stress increased from Wave 2 to Wave 3 and tended to decrease thereafter. Most change score variances were significant or approached significance, indicating individual differences in changes across the pandemic. Please note that the intercepts of the latent change factors are estimated based on the previous wave being 0, but 0 is an out-of-bound value in the scales used in this study. Therefore, the intercepts reported in Table 3 are not readily interpretable. To aid interpretation of these intercepts, Johnson-Neyman plots have been added in the Supplementary Material, for each intercept reported in Table 3. In those plots, the change score is plotted against the level of the previous time point.

**Table 1:** Means, Standard Deviations, and Bivariate Correlations among Study Variables

Variable	*W*	*M*	*SD*	1	2	3	4	5	6	7	8	9	10	11	12	13	14	15
1. Negative interaction	1	2.16	0.66															
2. Negative interaction	2	1.44	0.57	**.37**														
3. Negative interaction	3	1.46	0.53	**.44**	**.68**													
4. Negative interaction	4	1.49	0.56	**.41**	**.52**	**.61**												
5. Mental health problems	1	1.49	0.35	**.33**	.11	*.17*	.14											
6. Mental health problems	2	1.48	0.36	**.33**	**.32**	**.34**	*.20*	**.59**										
7. Mental health problems	3	1.50	0.42	**.30**	**.35**	**.39**	**.29**	**.50**	**.63**									
8. Mental health problems	4	1.53	0.42	**.20**	**.21**	**.23**	**.30**	**.48**	**.54**	**.57**								
9. Stress	2	1.70	0.72	−.04	−.03	.01	.04	**.22**	**.25**	**.23**	*.18*							
10. Stress	3	1.75	0.77	.02	.01	.10	−.03	*.15*	.11	**.33**	*.18*	**.35**						
11. Stress	4	1.64	0.78	.07	.01	.11	.11	.12	.12	**.21**	**.23**	**.27**	**.50**					
12. Extraversion	1	5.00	1.07	−.05	.09	−.01	−.04	−.*17*	−.12	−.**19**	−.*19*	−.12	.04	−.06				
13. Agreeableness	1	5.89	0.62	−.**17**	−.12	−.*17*	−.**27**	−.08	−.11	−.09	−.04	.10	.09	−.13	**.23**			
14. Conscientiousness	1	4.56	1.06	−.**21**	−.**26**	−.*18*	−.**22**	−.05	−.10	−.10	−.04	*.16*	.03	−.11	−.06	**.33**		
15. Emotional stability	1	4.24	1.12	−.*13*	.03	−.07	−.02	−.**36**	**−.24**	−.**34**	−.**38**	−.**22**	−.11	−.*19*	**.44**	.02	−.04	
16. Openness	1	5.25	0.81	−.03	−.12	−.03	−.09	−.01	−.06	.04	.03	.04	.12	−.00	**.18**	**.43**	**.19**	.04

Coefficients significant at *p* < .01 are shown in bold. Coefficients significant at *p* < .05 are shown in italic

*W* wave, *M* mean, *SD* standard deviation

**Table 2:** Fit Indices for the Revised Latent Change Score Models

Model Number	*χ*^*2*^	*df*	*p*	CFI	TLI	RMSEA [90%CI]	SRMR
Univariate Modeling							
1. Negative interaction	2.74	2	.254	.997	.991	.044 [.000–.227]	.028
2. Mental health problems	4.40	2	.111	.990	.971	.086 [.000–.219]	.023
3. Stress	0.55	1	.457	1.00	1.04	.000 [.000–.243]	.023
Multivariate Modeling							
4. All	30.16	35	.701	1.00	1.01	.000 [.000–.050]	.036
Personality Effects							
5. All Big Five - Multivariate LCS	109.78	59	.000	.938	.851	.066 [.041–.088]	.069
6. Openness - Multivariate LCS	64.09	47	.008	.976	.949	.045 [.000–.074]	.052
7. Conscientiousness - Multivariate LCS	77.42	47	.003	.960	.917	.058 [.026–.085]	.059
8. Extraversion - Multivariate LCS	67.24	47	.028	.973	.942	.048 [.000–.077]	.057
9. Agreeableness - Multivariate LCS	73.82	47	.008	.965	.926	.055 [.020–.082]	.056
10. Emotional stability - Multivariate LCS	92.18	47	.000	.945	.885	.069 [.042–.094]	.073

*N* = 244. Maximum Likelihood estimator was used, with Full Information Maximum Likelihood for handling missing data

*CFI* Comparative Fit Index, *TLI* Tucker-Lewis Index, *RMSEA* root mean square error of approximation, *90% CI* 90% confidence intervals, *SRMR* standardized root mean squared residual

**Table 3:** Unstandardized Intercepts, Variances, and Standardized Covariances Among Latent Change Scores of Negative Interaction, Mental Health, and COVID-19-related Stress, from Wave 1 (Fall 2019) to Wave 4 (Spring 2021)

				Standardized Covariances				
Variable	Period	Intercept	Variance	4	5	6	7	8
1. Negative interaction	W1-W2	0.605***	0.585*	.716*				
2. Negative interaction	W2-W3	0.239^	0.876^		.422^		.216	
3. Negative interaction	W3-W4	0.230	0.969*			.480*		.214
4. Mental health problems	W1-W2	−0.046	0.997					
5. Mental health problems	W2-W3	0.002	0.999^				.641**	
6. Mental health problems	W3-W4	0.253	0.947**					.218
7. Stress	W2-W3	0.658*	0.885*					
8. Stress	W3-W4	0.512^	0.796					

*W* wave. Please note that the intercepts of the latent change factors are estimated based on the previous wave being 0, but 0 is an out-of-bound value in the scales used in this study. Therefore, the intercepts reported here are not readily interpretable. To aid interpretation of the intercepts, Johnson-Neyman plots have been added for each intercept reported above in the Supplementary Material

***p < .001; **p < .01; *p < .05, ^*p* < .10

In addition to the mean level changes, the multivariate model examined whether changes in one variable were associated with changes in another. An increase in parent-adolescent negative interactions between Wave 1 and Wave 2 was associated with an increase in mental health problems during the same time; the same applied for changes between Wave 3 and Wave 4, although the association was less strong. Also, increases in COVID-19-health-related stress between Wave 2 and Wave 3 were associated with increases in mental health problems during the same period.

To test the effect of personality characteristics on the changes in negative interactions, mental health problems, and COVID-19-health-related stress, all Big Five factors were added simultaneously to the multivariate model mentioned above. As can be seen in Table 2, the fit of this model was suboptimal. Therefore, the effects of each personality characteristic on the multivariate LCS model were modeled separately. As seen in the first column of Table 4, only two significant effects emerged. Adolescents higher in extraversion at Wave 1 (before the pandemic) experienced a stronger increase in their COVID-19 related stress between Wave 2 and 3. Adolescents with higher levels of pre-pandemic agreeableness showed a stronger decrease in COVID-19-health-related stress between Wave 3 and Wave 4. Finally, several other personality effects were found to be “trends” (*p*-values < .10), all of which were further supported by the Bayesian sensitivity analyses (see below).

**Table 4:** Regression Estimates of Frequentist and Bayesian Model Estimation for the Effects of Personality on the Latent Change Scores

		Frequentist	Bayesian							
Variable	Change	β	Estimate	Posterior S.D.	Lower CI	Higher CI	β	PSRF (Ȓ)	ESS	Probability^a^
Openness										
Negative interaction	W1-W2	−.086	−0.064	0.048	−0.161	0.032	−.088	1.001	2841	.87
Negative interaction	W2-W3	.019	0.010	0.041	−0.070	0.091	.042	1.001	2187	.51
Negative interaction	W3-W4	−.103	−0.042	0.048	−0.132	0.055	−.106	1.000	2691	.75
Mental health problems	W1-W2	−.069	−0.014	0.027	−0.068	0.040	−.077	1.001	2966	.56
Mental health problems	W2-W3	.201	0.046	0.034	−0.024	0.114	.230	1.000	2356	.86
Mental health problems	W3-W4	.002	0.001	0.035	−0.070	0.069	.003	1.000	3352	.38
Stress	W2-W3	.151^	0.119	0.071	−0.019	0.260	.190	1.000	2642	.94
Stress	W3-W4	−.111	−0.103	0.077	−0.258	0.042	−.238	1.001	2516	.89
Conscientiousness										
Negative interaction	W1-W2	−.163	−0.095	0.036	−0.169	−0.023	−.158	1.001	3883	.99
Negative interaction	W2-W3	−.082	−0.010	0.032	−0.071	0.057	−.054	1.000	2611	.50
Negative interaction	W3-W4	−.097	−0.027	0.036	−0.099	0.043	−.089	0.999	3584	.69
Mental health problems	W1-W2	−.166	−0.024	0.021	−0.064	0.017	−.171	1.001	3729	.75
Mental health problems	W2-W3	.019	0.005	0.025	−0.044	0.055	.036	1.000	3234	.43
Mental health problems	W3-W4	.085	0.020	0.026	−0.028	0.071	.091	0.999	3896	.66
Stress	W2-W3	−.088	−0.041	0.056	−0.151	0.071	−.087	1.001	3453	.71
Stress	W3-W4	−.156	−0.080	0.056	−0.190	0.031	−.256	1.000	3451	.89
Extraversion										
Negative interaction	W1-W2	.091	0.053	0.036	−0.017	0.123	.098	0.999	3833	.89
Negative interaction	W2-W3	−.043	−0.015	0.031	−0.075	0.047	−.079	1.000	3355	.56
Negative interaction	W3-W4	−.098	−0.032	0.037	−0.104	0.041	−.107	0.999	4288	.72
Mental health problems	W1-W2	−.079	−0.011	0.022	−0.055	0.032	−.082	0.999	3679	.54
Mental health problems	W2-W3	−.096	−0.016	0.026	−0.066	0.036	−.104	1.000	3244	.59
Mental health problems	W3-W4	−.091	−0.019	0.027	−0.071	0.034	−.085	0.999	3777	.64
Stress	W2-W3	.154*	0.091	0.054	−0.019	0.198	.191	0.999	4269	.93
Stress	W3-W4	−.094	−0.064	0.056	−0.178	0.040	−.190	0.999	3559	.84
Agreeableness										
Negative interaction	W1-W2	−.016	−0.016	0.063	−0.138	0.102	−.018	0.999	2069	.54
Negative interaction	W2-W3	−.212	−0.088	0.049	−0.183	0.006	−.273	1.000	1968	.94
Negative interaction	W3-W4	−.171	−0.085	0.060	−0.199	0.032	−.167	1.001	1790	.90
Mental health problems	W1-W2	−.123	−0.030	0.036	−0.102	0.044	−.124	1.000	1635	.71
Mental health problems	W2-W3	−.015	−0.002	0.043	−0.085	0.080	−.008	0.999	1566	.43
Mental health problems	W3-W4	.071	0.031	0.043	−0.054	0.115	.082	0.999	1846	.69
Stress	W2-W3	.025	0.033	0.090	−0.144	0.206	.041	1.002	1844	.60
Stress	W3-W4	−.227*	−0.211	0.094	−0.392	−0.034	−.387	1.000	1912	.99
Emotional Stability										
Negative interaction	W1-W2	.061	0.034	0.037	−0.038	0.103	.065	1.000	3782	.73
Negative interaction	W2-W3	−.070	−0.017	0.029	−0.073	0.040	−.095	1.000	4919	.61
Negative interaction	W3-W4	.024	0.007	0.035	−0.060	0.074	.026	1.000	5354	.48
Mental health problems	W1-W2	−.049	−0.009	0.022	−0.052	0.033	−.064	0.999	3239	.48
Mental health problems	W2-W3	−.246^	−0.038	0.024	−0.084	0.010	−.255	0.999	3182	.88
Mental health problems	W3-W4	−.183^	−0.037	0.027	−0.087	0.016	−.172	1.000	3142	.84
Stress	W2-W3	.016	0.009	0.053	−0.092	0.112	.020	1.000	4964	.51
Stress	W3-W4	−.151^	−0.072	0.054	−0.177	0.032	−.211	0.999	3960	.88

“Estimate” denotes unstandardized estimates, while “β” denotes standardized estimates. *W* wave, *CI* credible interval, *PSRF* Potential Scale Reduction Factor, *Ȓ* R-hat, *ESS* Effective Sample Size (a measure of MCMC chain resolution, or else of Bayesian estimation resolution, unrelated to the actual study participants). The effects of each personality factor were tested in different models

^a^Probability of direction test (Makowski et al., 2019). Depending on whether the unstandardized estimate has a positive or a negative sign, the probability of direction test was applied to examine the probability that the coefficient is larger than .01 or smaller than −.01, respectively (.00 was avoided as there is little chance that a coefficient is absolutely zero). For example, regarding the effect of Openness on the change in COVID-19-related stress between wave 2 and wave 3 of this study, the unstandardized estimate is .119, and there is 94% probability that this coefficient is actually positive, and higher than .01, even if the prediction intervals contain zero. For this test, an alpha level of .05 was chosen

*p < .05, ^*p* < .10

Table 4 also shows the results of the Bayesian analyses, including the parameter estimates and the convergence diagnostics. Based on the PSRF (Ȓ), Effective Sample Size, and the 40 trace plots for the main parameters of interest (for the latter, see Supplementary Material), these Bayesian analyses reached satisfactory convergence. These analyses support the significant effects for extraversion (on the change in stress from Wave 2 to 3) and agreeableness (on the change in stress from Wave 3 to 4). Furthermore, these results support the four effects that showed a “trend” (*p* < .10) in the frequentist analyses, namely the positive effect of openness on change in stress from Wave 2 to Wave 3 (94% probability of the effect *b* > .01), and the negative effects of emotional stability on change in mental health problems from Wave 2 to Wave 3, and from Wave 3 to Wave 4, as well as on change in stress from Wave 3 to Wave 4 (88%, 84%, and 88% probability of *b* < −.01, respectively). Thus, higher openness to experiences tends to predict an increase in COVID-related stress between Wave 2 and 3. Adolescents with higher emotional stability pre-pandemic tended to experience a stronger decrease in mental health problems between waves 2 and 3 as well as between Wave 3 and 4, and a stronger decrease in stress between Wave 3 and 4. Finally, the Bayesian analyses indicated several other personality effects with a probability larger than 80% being different from zero (see Table 4).

## Discussion

Many studies have investigated effects of COVID-19 on the lives of adolescents during the first period of the pandemic. However, much is unclear about the impact of the pandemic the longer it lasted as well as about potential interindividual differences in resilience to the effects of the pandemic. The current study therefore aimed to examine changes in adolescents’ COVID-19-health-related stress, parent-adolescent relationship quality, and mental health problems across a time span of 1.5 years (i.e., Fall 2019-Spring 2021), as well as the longitudinal associations among these trajectories. Moreover, it was investigated whether personality traits could explain interindividual differences in the changes over time. Most of the hypotheses were only partially or not supported. Negative interactions decreased from before the pandemic to the first lockdown, but then remained stable. Mental health problems and COVID-19-health-related stress remained mostly stable throughout all the phases of this study. There were individual differences in the changes, which could only to a small extent be explained by adolescent personality. Although some of the findings might be specific for the COVID-19 pandemic, it can be expected that most of the findings would also apply to other large-scale disasters, such as earthquakes or floods.

### Changes in Parent-Adolescent Relationships

In line with previous research (Donker et al., 2021), it was found that the average level of negative interactions in the sample decreased from before the pandemic to just after the first lockdown period in spring 2020. However, this decline remained stable and did not carry through in the later phases of the pandemic. Parents and adolescents might thus have been able to diminish their negative interactions during the first lockdown and retained these more positive interaction patterns later during the pandemic. It was also found that adolescents who reported a stronger decline in negative interactions in this first phase of the pandemic experienced a stronger decline (or less increase) in mental health problems in the same period. This suggests that parents have an important protective role against mental health problems in youth, and it suggests that parents adjust their parenting in line with changes in adolescents’ functioning. This is in line with previous findings on the important role of family processes for adolescent development in challenging times, for example in the transition to college (Guassi Moreira & Telzer, 2015; Sasser et al., 2023). Thus, targeting the family as a whole might be worthwhile to improve adolescent well-being by improved family relationships. Moreover, it could potentially also foster parental well-being and thereby their potential to protect and guide their offspring in adverse situations (Masten & Motti-Stefanidi, 2020).

The current study also found a significant association between changes in negative interactions and changes in mental health problems between Wave 3 and 4. Again, in those families that better adapted to the new situation by reducing (or not increasing) their negative interactions, the adolescents also reported a stronger decline in mental health problems. Here the start of the vaccination campaign might also have played a role. For those adolescents who experienced much relief and less mental health problems due to the positive prospects, the interactions with their parents might have also benefited.

### Changes in COVID-19-Health-Related Stress

Regarding changes in stress, an association was found with mental health problems only between Wave 2 and 3. During Wave 2, schools were already partly reopened and in general the regulations were loosened in the summer period. Both for adolescents and for parents, reopening of schools and being able to spend time outside might have put the pressure off the family system and it might have given adolescents opportunities to share their worries and issues with peers, which released both stress and mental health problems. Wave 3 took place during the second lockdown period. Therefore, in a more negative sense, those who were already vulnerable might have experienced heightened stress levels as well as an increase in mental health problems during this period, which could explain the association in this specific time frame. Also, during this period, the adolescents in the sample made the transition to secondary school, which could have amplified the link between stress and mental health.

Adolescents scoring higher on extraversion at Wave 1 (before the pandemic) experienced a stronger increase in their COVID-19 related stress between Wave 2 and 3, which is in line with the expectations that this group would be hit extra hard by the restrictive measures and the perspective of a second winter in lockdown. The effect did not continue to the fourth wave. It could be that this mainly has to do with the relatively young age of the participants and the precautious measures that were taken to reduce the impact of the restrictive measures on this group. Especially in the later phases of the pandemic, many of the contact-restrictive measures in the Netherlands did not apply to them, and thus they could go to school and meet with friends. For extraverted (young) adults, the consequences of the enduring restrictive measures might have been much more severe.

For adolescents with high levels of agreeableness and emotional stability (though the latter was only marginally significant), a stronger decrease in stress from the start of the second lockdown period in Fall 2020 (Wave 3) to the end of this period in Spring 2021 (Wave 4) was found. However, contrary to previous findings (e.g., Bellingtier et al., 2021), this effect was not significant in the earlier waves of the pandemic. The decrease in stress especially in the later phases of the pandemic might be associated with the better future perspectives in the beginning of 2021, when the vaccination campaigns started, and everything was supposed to go back to normal relatively soon. Especially agreeable and emotional stable adolescents might have been glad that they could do something to mitigate the effects of the virus by getting vaccinated themselves. This is in line with their heightened tendency to show prosocial behavior (Kline et al., 2019) and to adhere to governmental guidelines during the pandemic (Moore et al., 2022). That is, they might have been more willing to get vaccinated to protect others and would have confidence that others would do the same. For those more emotionally stable adolescents, the decrease in stress is potentially also associated with their tendency to show a stronger decrease in mental health problems both between Wave 2 and 3 and between Wave 3 and 4. These adolescents thus seem to ‘bounce back to normal’ more easily than their peers, which is in line with previous findings (Zager Kocjan et al., 2021).

### Changes in Mental Health Problems

Contrary to the expectations and to some of the extant empirical evidence (Racine et al., 2021), there was no significant mean level change in mental health symptoms throughout the pandemic; on average, mental health symptoms seemed to be stable. This finding potentially reflects the general well-being of Dutch youth who, in international studies are among the best-faring youth in terms of well-being (Inchley et al., 2020). One recent study in the Netherlands also found that for most adolescents the pandemic did not have a detrimental effect on mental health (Bouter et al., 2022). However, significant variance around the mean implied significant heterogeneity, thus adolescents differ in how their mental health developed throughout the pandemic. Some adolescents might have experienced a decline, while others increased in mental health problems (Bouter et al., 2022). The current study showed that personality factors could explain this heterogeneity only to a limited extent. Adolescents lower in emotional stability before the pandemic tended to experience a stronger increase in mental health symptoms both from Wave 2 to Wave 3 as well as from Wave 3 to Wave 4. This is in line with other studies who found that adolescents who were at risk for problems before the pandemic also reported most problems during the pandemic (Panchal et al., 2021). Especially for mental health, having (other) pre-existing symptoms combined with limited access to mental health care during the pandemic, might have led to an increase of problems in this group. During future large-scale (natural) disasters, vulnerable groups thus might need extra attention and support. Also, access to mental health care seems something that needs to be guaranteed in all situations for those that need it the most, for example via online counseling. Health care organizations could prepare for future disasters, like the COVID-19 pandemic, by improving their digital competencies (Barker & Barker, 2022).

### Limitations and Future Directions

The participants in our sample were a well-functioning group, which might have restricted us from finding strong effects on stress and mental health problems. Furthermore, participants made a school transition in the middle of the data-collection period and the COVID-related restrictions might have differed a bit between participants in the same wave. However, the overall stability in the group means suggests that these factors might not have affected the findings to a substantial extent. The relatively small sample size might have resulted in power issues, which could explain the limited number of significant effects. Also, both Wave 2 and Wave 4 took place mainly after the strict lockdown periods, which might have affected the potential of this study to detect changes. Some of the marginally significant findings could give direction to what future studies could explore in larger datasets or with more sensitive designs. More methodologically, problems with measurement invariance restricted us from drawing conclusions on the changes in parental support during the pandemic, although this might be a highly relevant factor in adolescent stress and well-being. Also, although the relationship quality items were formulated in the present time, the wording of the instructions for the relationship quality questionnaire did not specify a concrete time period. There might thus have been overlap in the responses in the various waves, which could explain the lack of change in the later waves. Furthermore, the Openness to Experience scale had low reliability, thus the results for this scale should be interpreted with caution. Finally, this study used only adolescent-reported variables, which could have led to common-rater (and thus amplified) effects (Podsakoff et al., 2003). Future studies could improve our understanding of the processes by adding parent reports (Mastrotheodoros et al., 2020) or observation (Donker et al., 2021) of relationship quality, stress, and mental health problems.

## Conclusion

Previous studies investigated adolescent well-being during the early phases of COVID-19. However, there was limited information about the long-term impact of the pandemic as well as about potential interindividual differences between adolescents. The current study therefore examined changes in adolescents’ stress, mental health, and relationship quality over a longer period (i.e., up to 1.5 year into the pandemic). Moreover, by investigating the effects of personality traits on changes during the pandemic, the current study was able to tap into a potential factor explaining heterogeneity in the effects. On average, both COVID-19-health-related stress and mental health problems remained stable throughout the pandemic in our sample of Dutch adolescents. Negative interactions between parents and adolescents decreased from before the pandemic to the first lockdown, and then remained stable as well. This suggests that, on average, Dutch youth were resilient and have not suffered much from the situation. The current study also confirmed that adolescents differed in their responses to the COVID-19 pandemic, and that the average effects might not hold for all adolescents. There were some significant associations among the changes in stress, mental health problems, and negative interactions. Especially decreases in negative interactions (between Fall 2019-Spring 2020 and Fall 2020-Spring 2021) and stress (from Spring-Fall 2020) were associated with improved mental health in the same period. This shows that resilience in one setting (for example the family setting) might help to protect against the development of psychological problems in the adolescent, even in the case of a pandemic. Using a multisystem approach might thus be worthwhile, also during disasters. Although personality was not a very influential factor, some personality characteristics could explain part of the heterogeneity in changes over the course of the pandemic. Especially those adolescents who scored low on agreeableness, emotional stability, and/or openness to experience might need extra support during challenging situations to prevent negative effects on stress and mental health. Extraverted individuals might also need extra attention in situations like the pandemic, where their usual support system and active coping mechanisms might be disabled. Thus, although youth appear to be relatively resilient, especially adolescents with existing problems and from multi-problem families might be at risk for adverse consequences during pandemic-like situations.

## Preregistration

The research questions, hypotheses, and data analysis plan were preregistered at 10.17605/OSF.IO/J9SCW.

## Supplementary Information

Supplementary Material #1

Supplementary Material #2
