# Supplementary material for: Changes in Adolescents’ COVID-19-Health-Related Stress, Parent-Adolescent Relationships, and Mental Health During the COVID-19 Pandemic: The Effect of Personality Traits
Source: J Youth Adolesc. 2024 Jul 23;54(1):209–24. doi: 10.1007/s10964-024-02048-w (PMC11742768; doi:10.1007/s10964-024-02048-w)
Supplement: Supplementary file 1 — Supplementary Material #1 [file 10964_2024_2048_MOESM1_ESM.docx]

Supplementary Material #1 – Johnson-Neyman plots


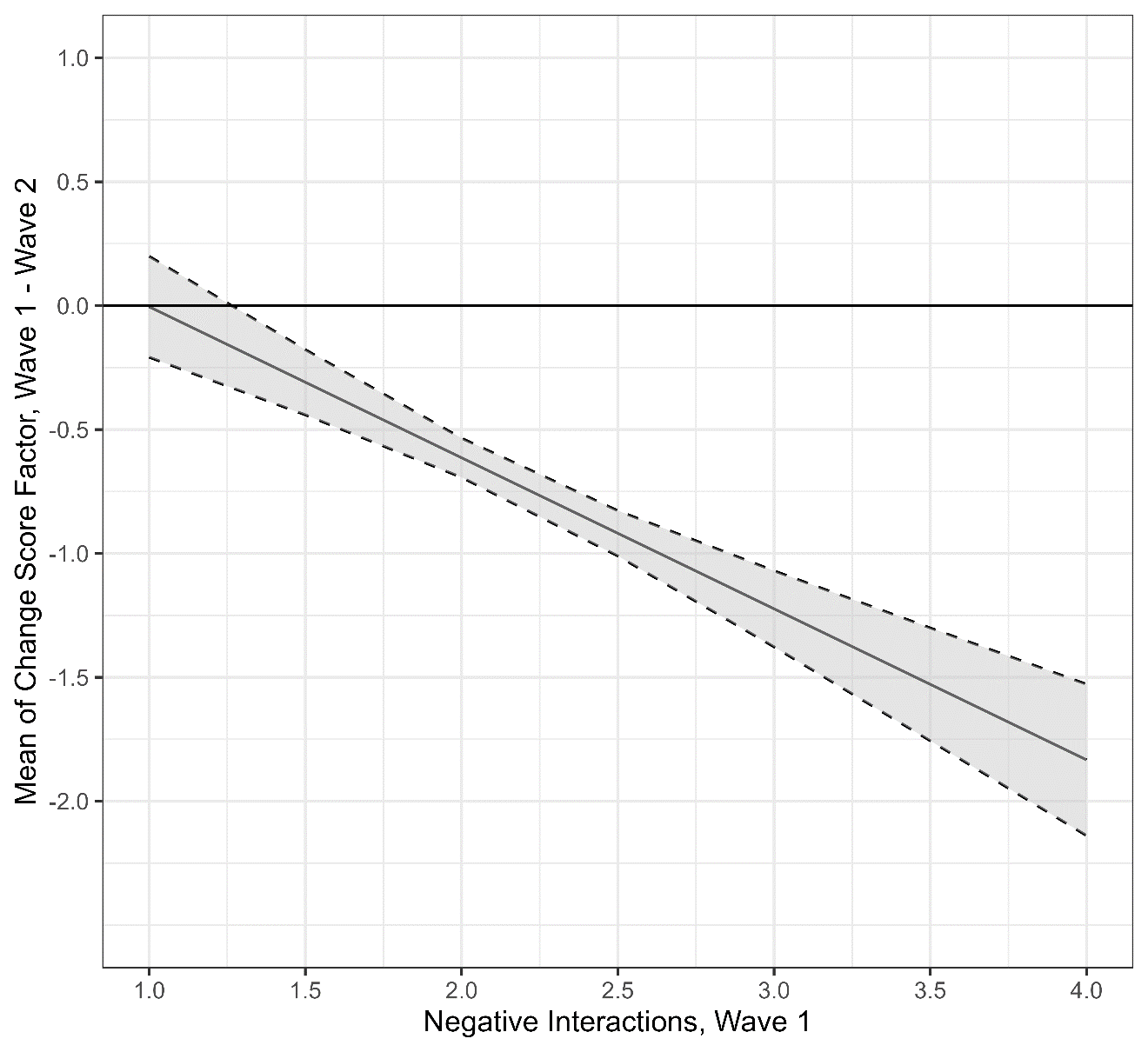


Figure 1. Latent Change Score Mean of Negative Interactions from Wave 1 to Wave 2 Plotted Against Previous Wave Negative Interaction Score.


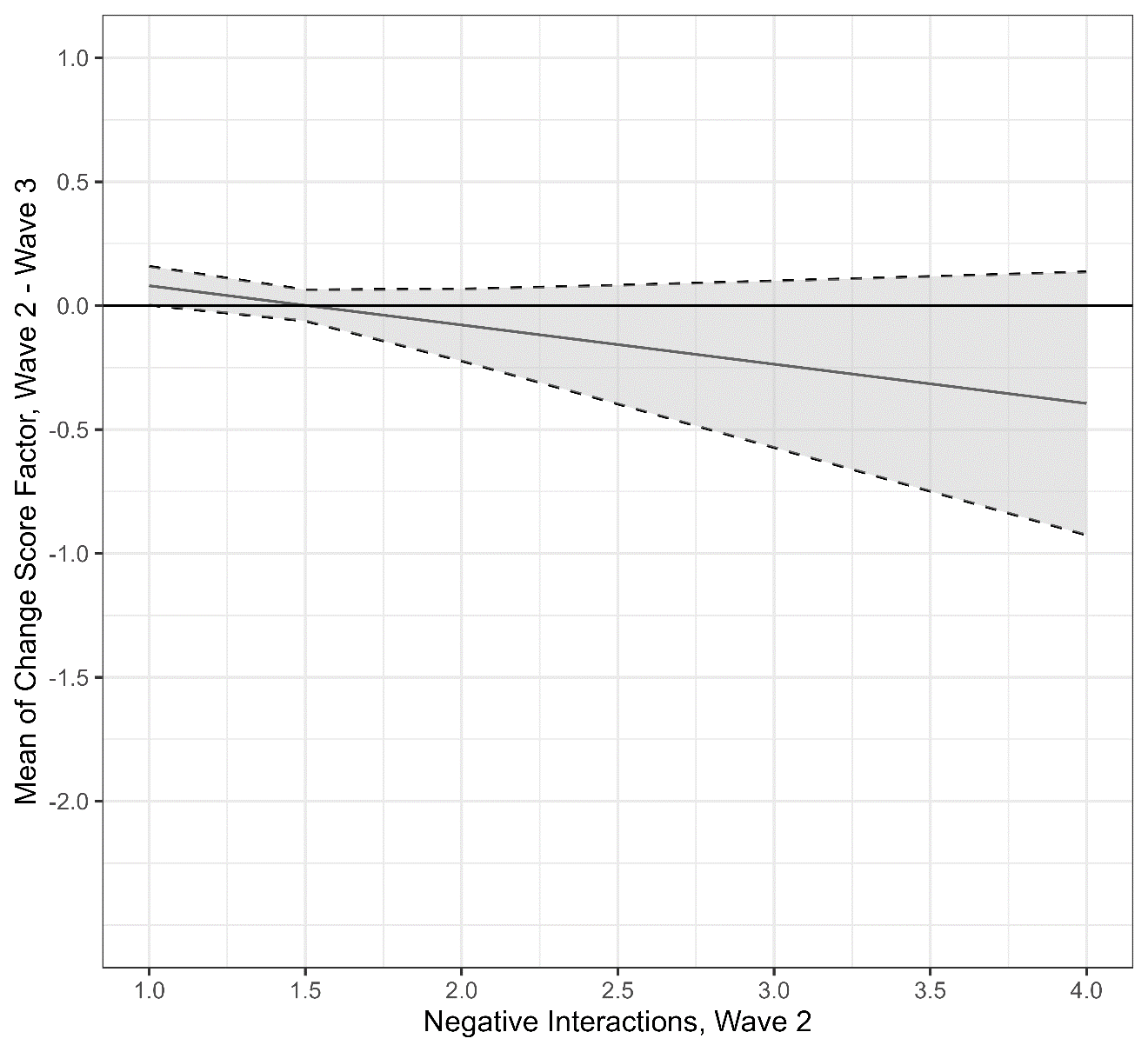


Figure 2. Latent Change Score Mean of Negative Interactions from Wave 2 to Wave 3 Plotted Against Previous Wave Negative Interaction Score.


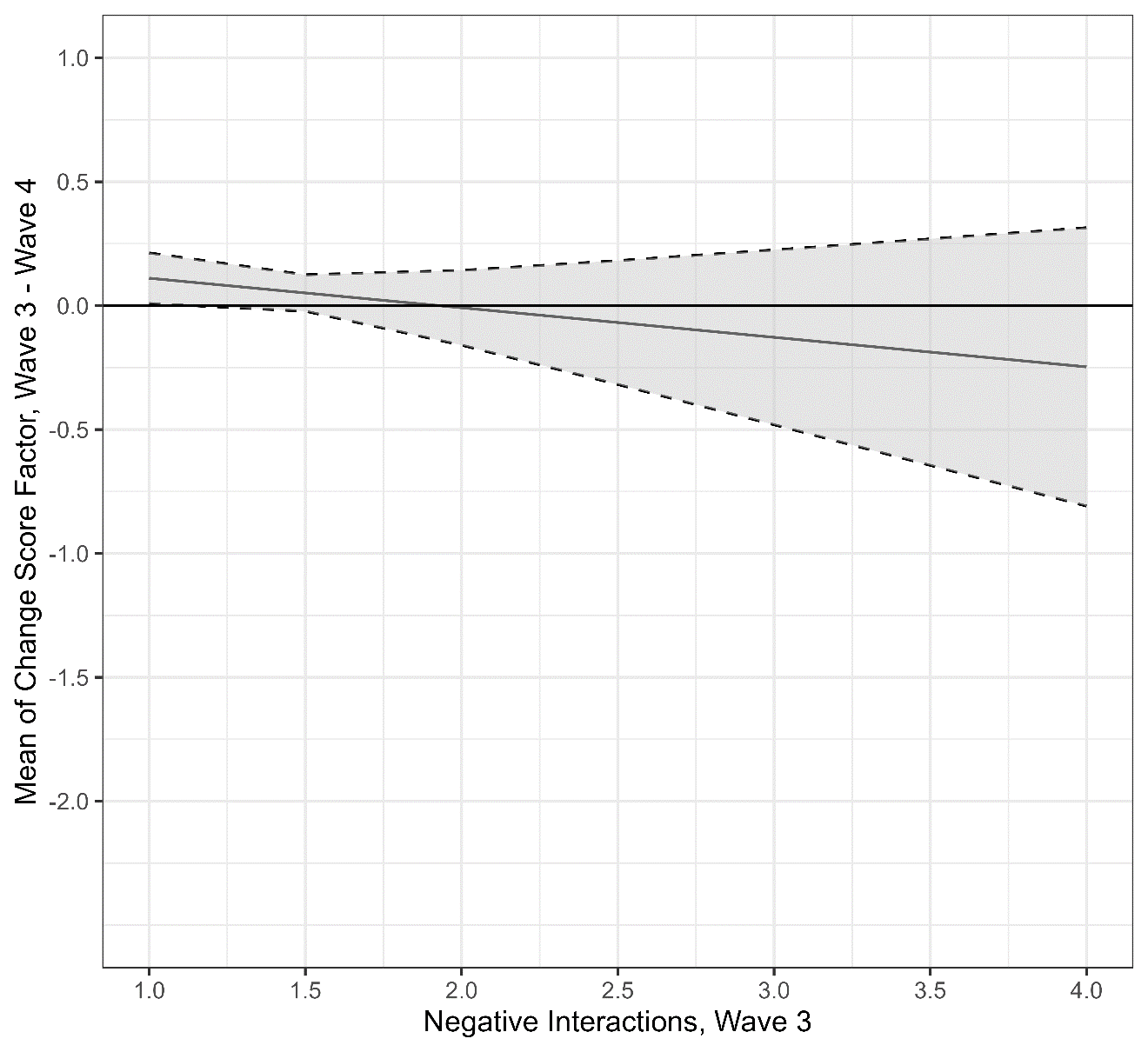


Figure 3. Latent Change Score Mean of Negative Interactions from Wave 3 to Wave 4 Plotted Against Previous Wave Negative Interaction Score.


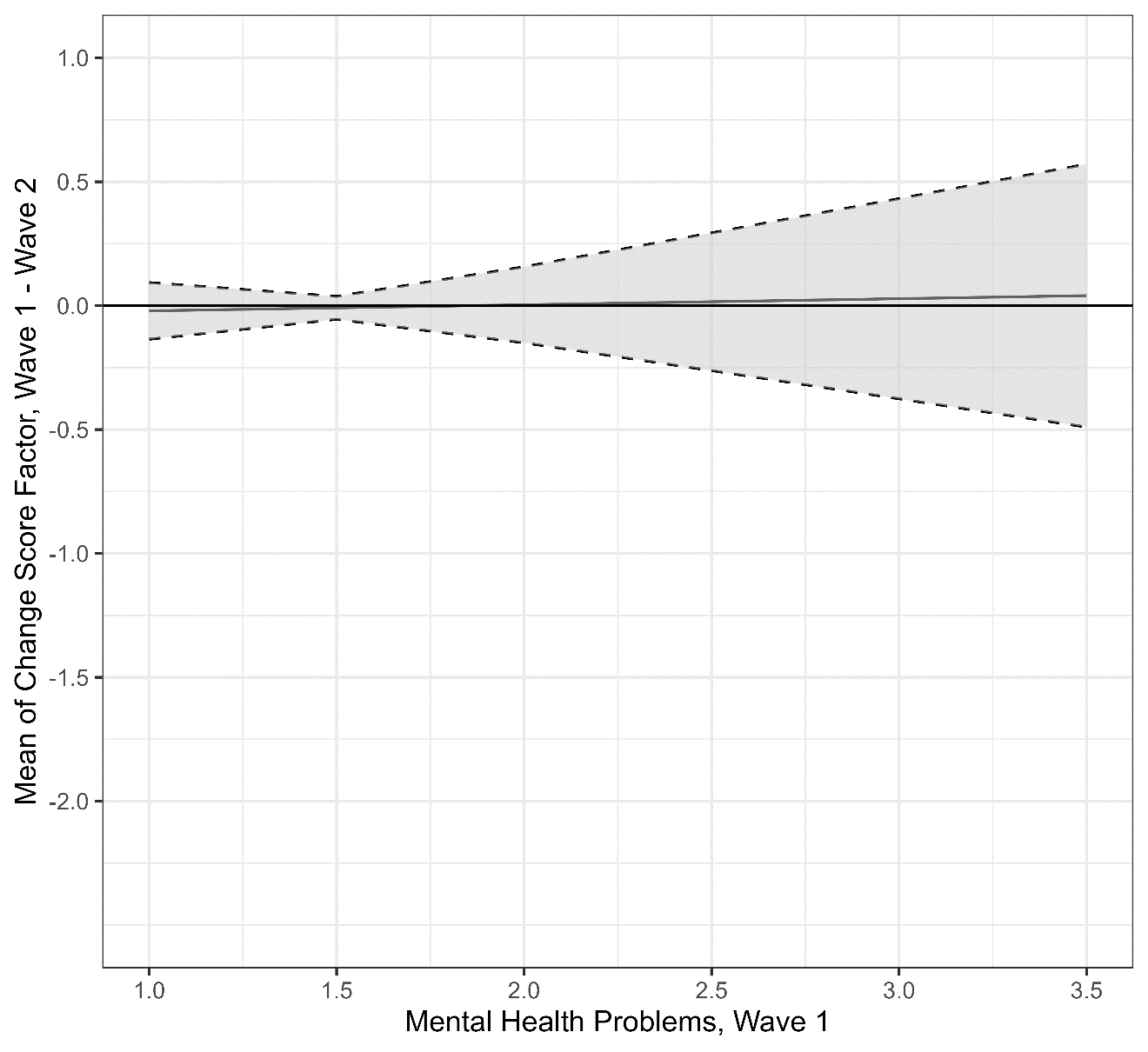


Figure 4. Latent Change Score Mean of Mental Health Problems from Wave 1 to Wave 2 Plotted Against Previous Wave Mental Health Problems Score.


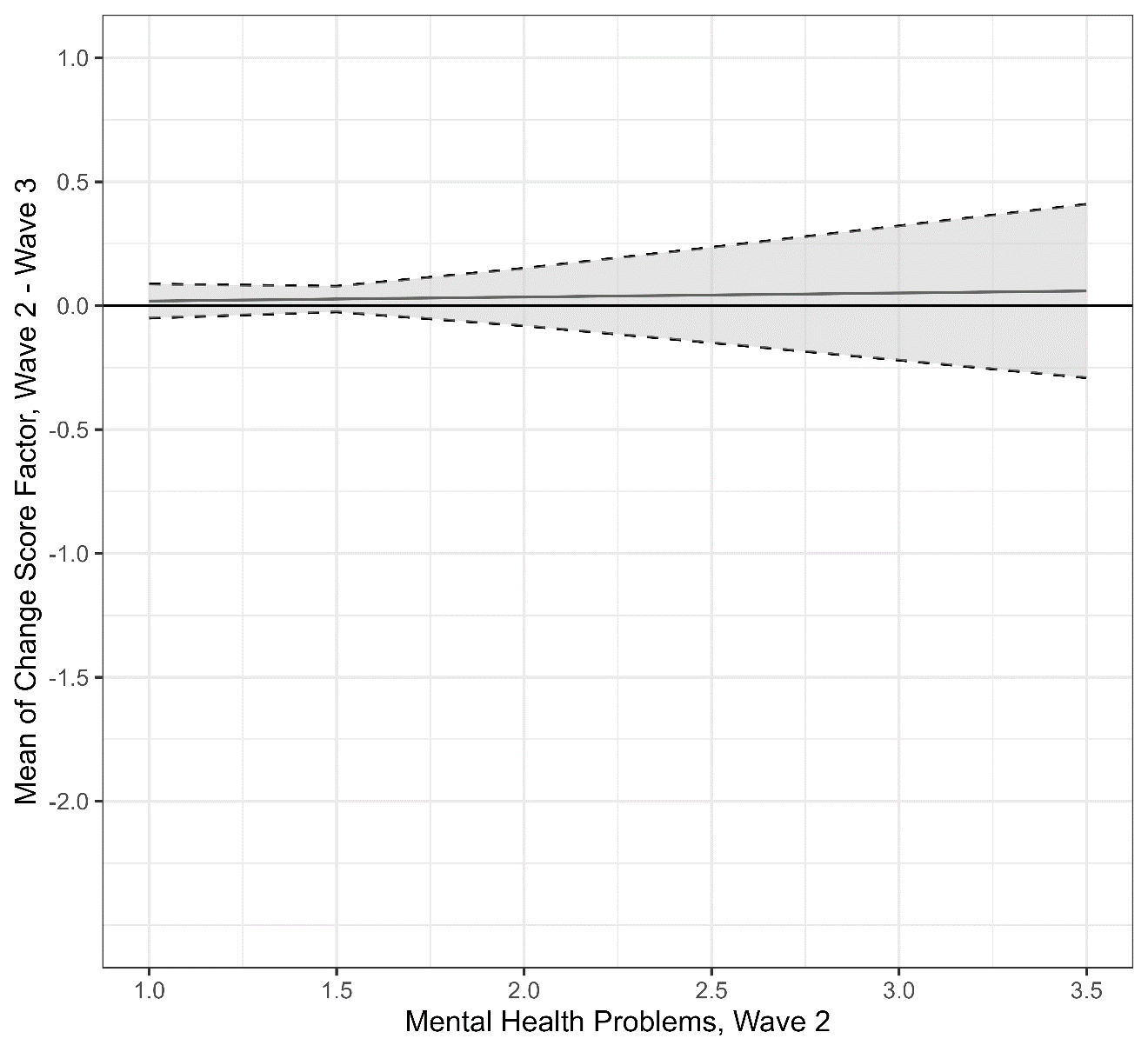


Figure 5. Latent Change Score Mean of Mental Health Problems from Wave 2 to Wave 3 Plotted Against Previous Wave Mental Health Problems Score.


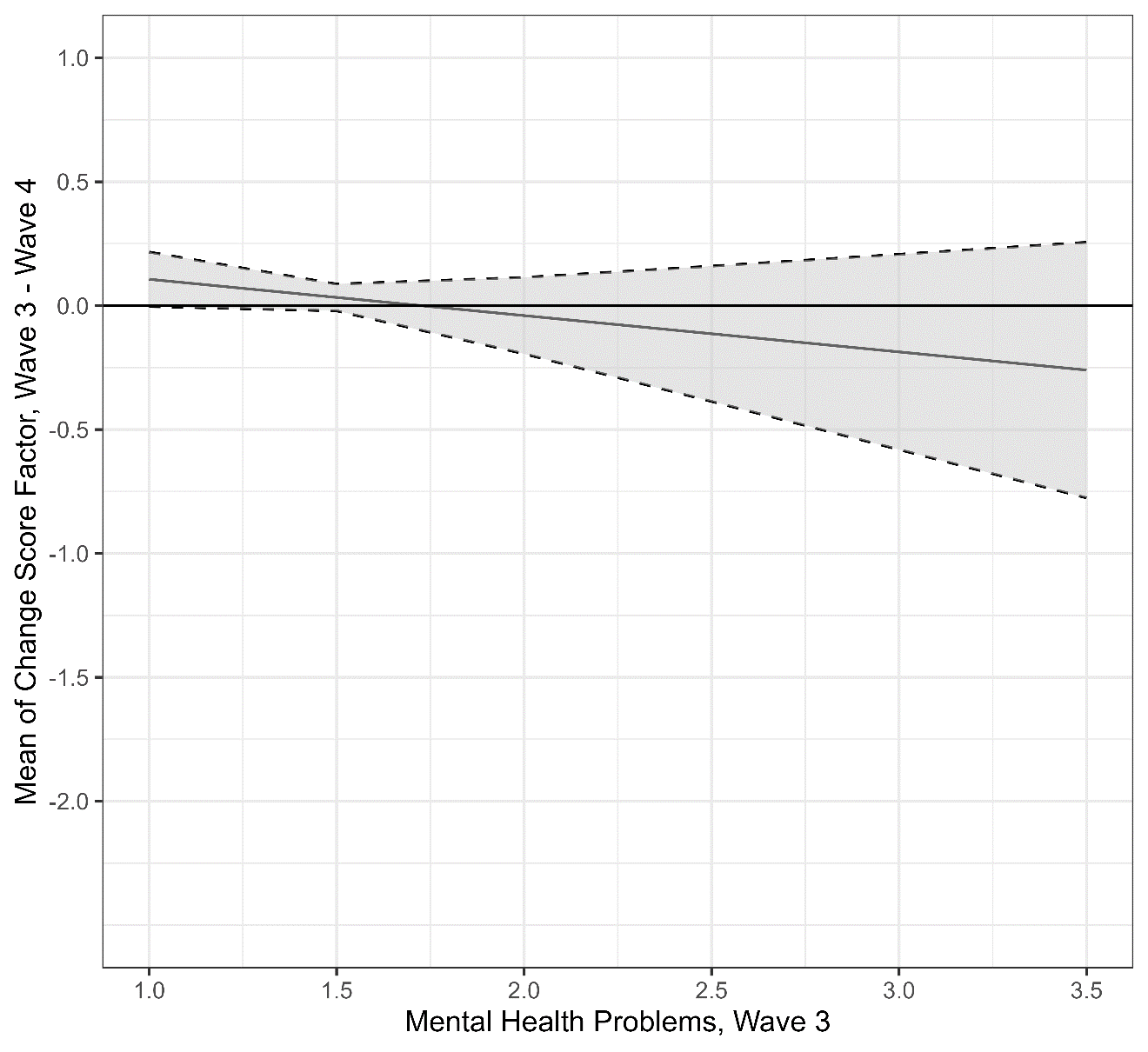


Figure 6. Latent Change Score Mean of Mental Health Problems from Wave 3 to Wave 4 Plotted Against Previous Wave Mental Health Problems Score.


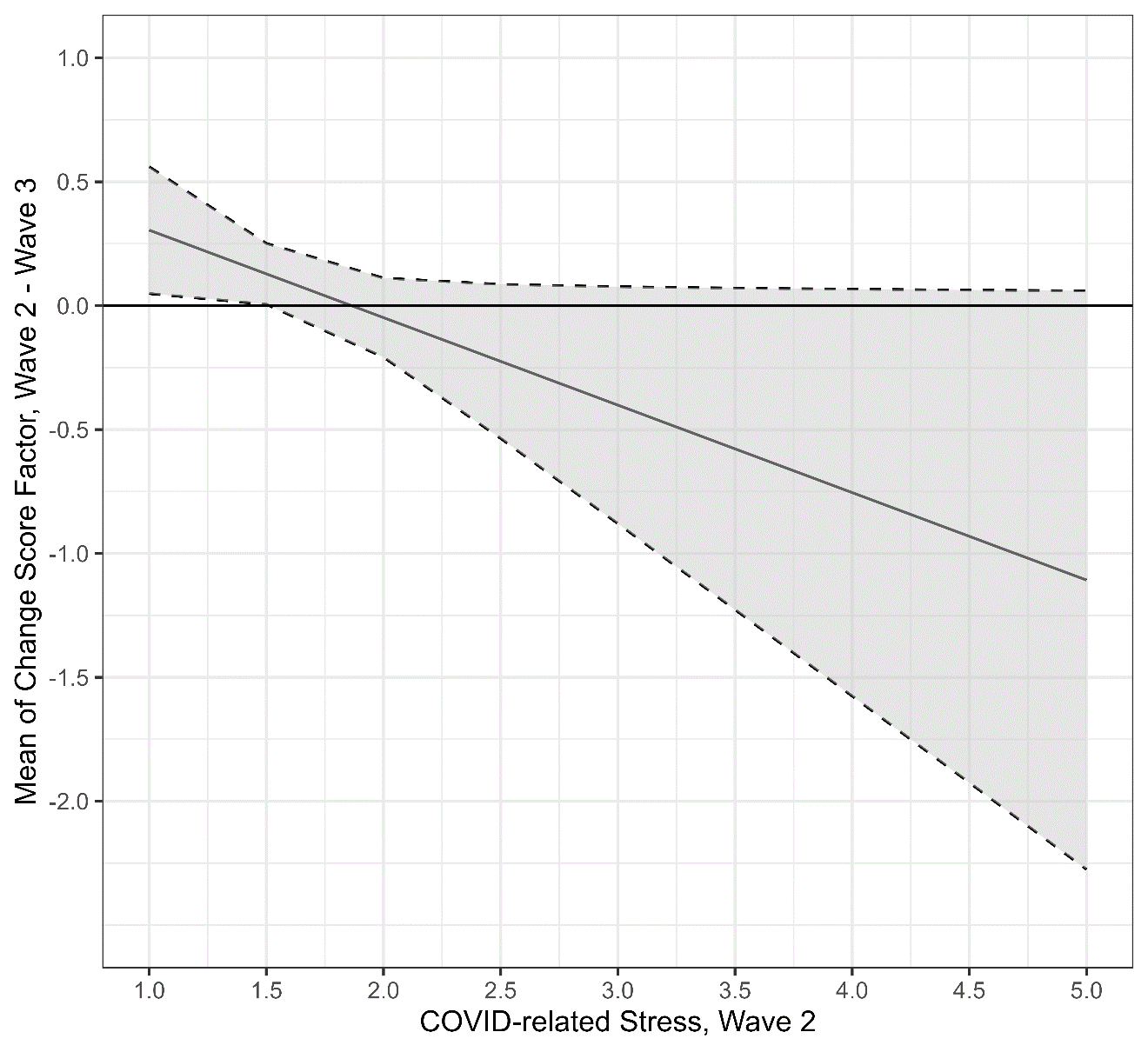


Figure 7. Latent Change Score Mean of COVID-related Stress from Wave 2 to Wave 3 Plotted Against Previous Wave COVID-related Stress Score.


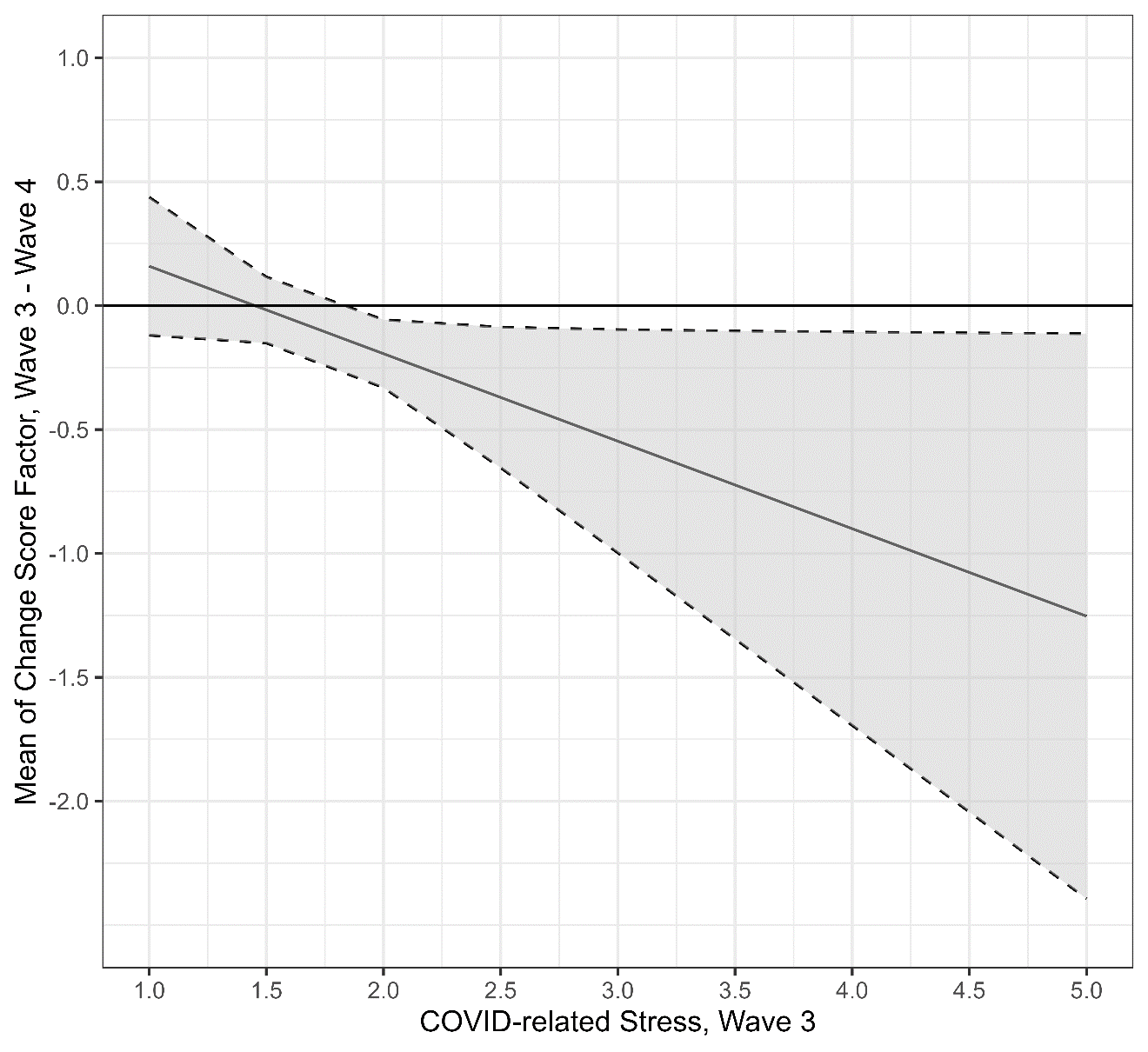


Figure 8. Latent Change Score Mean of COVID-related Stress from Wave 3 to Wave 4 Plotted Against Previous Wave COVID-related Stress Score.
