# Supplementary material for: Changes in Adolescents’ COVID-19-Health-Related Stress, Parent-Adolescent Relationships, and Mental Health During the COVID-19 Pandemic: The Effect of Personality Traits
Source: J Youth Adolesc. 2024 Jul 23;54(1):209–24. doi: 10.1007/s10964-024-02048-w (PMC11742768; doi:10.1007/s10964-024-02048-w)
Supplement: Supplementary file 2 — Supplementary Material #2 [file 10964_2024_2048_MOESM2_ESM.docx]

Supplementary Material #2 - Traceplots

Effects of Openness to Experience


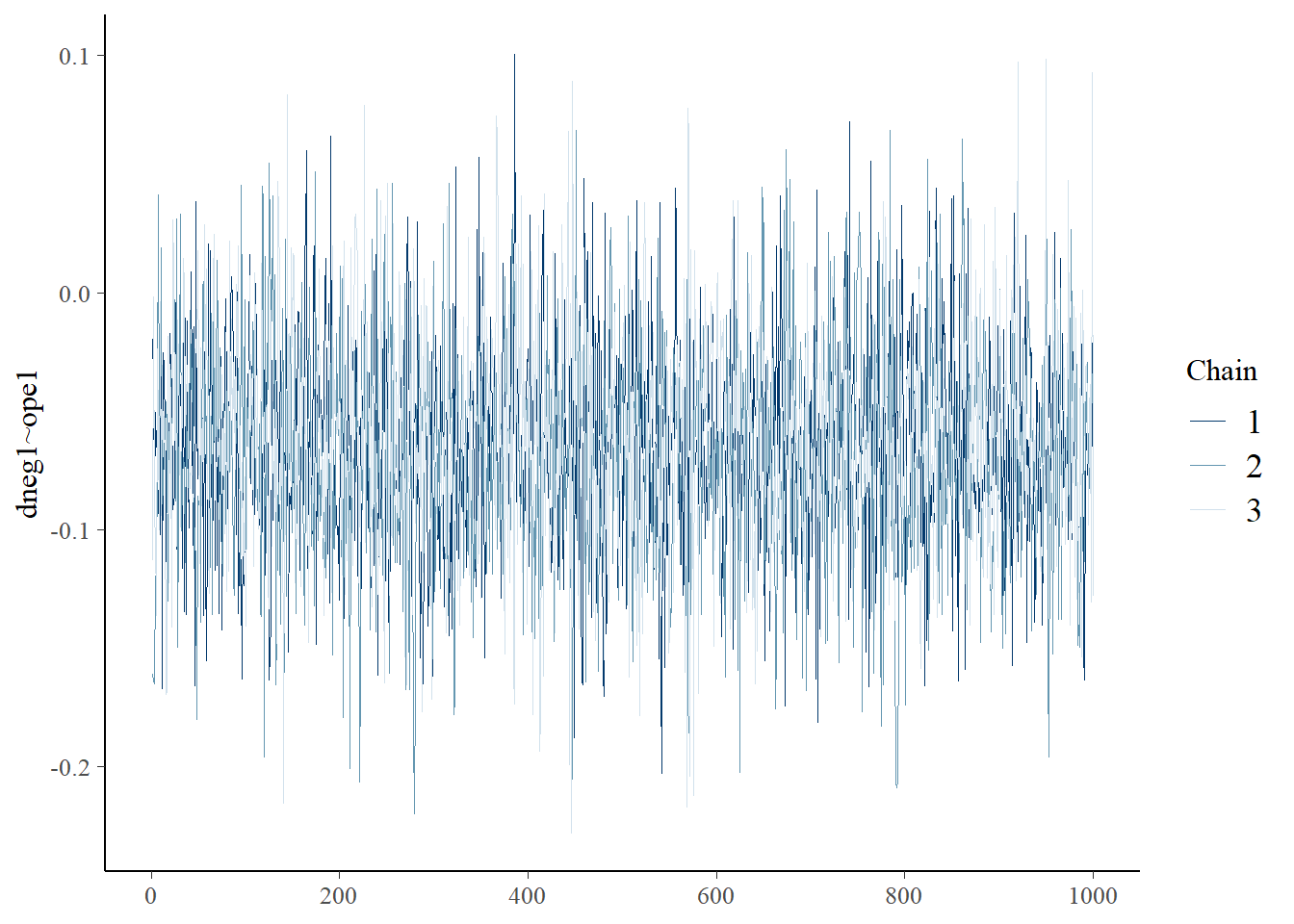


Figure 1. Traceplot of the effect of Openness to Experience on change in Negative Interactions between Wave 1 and Wave 2.


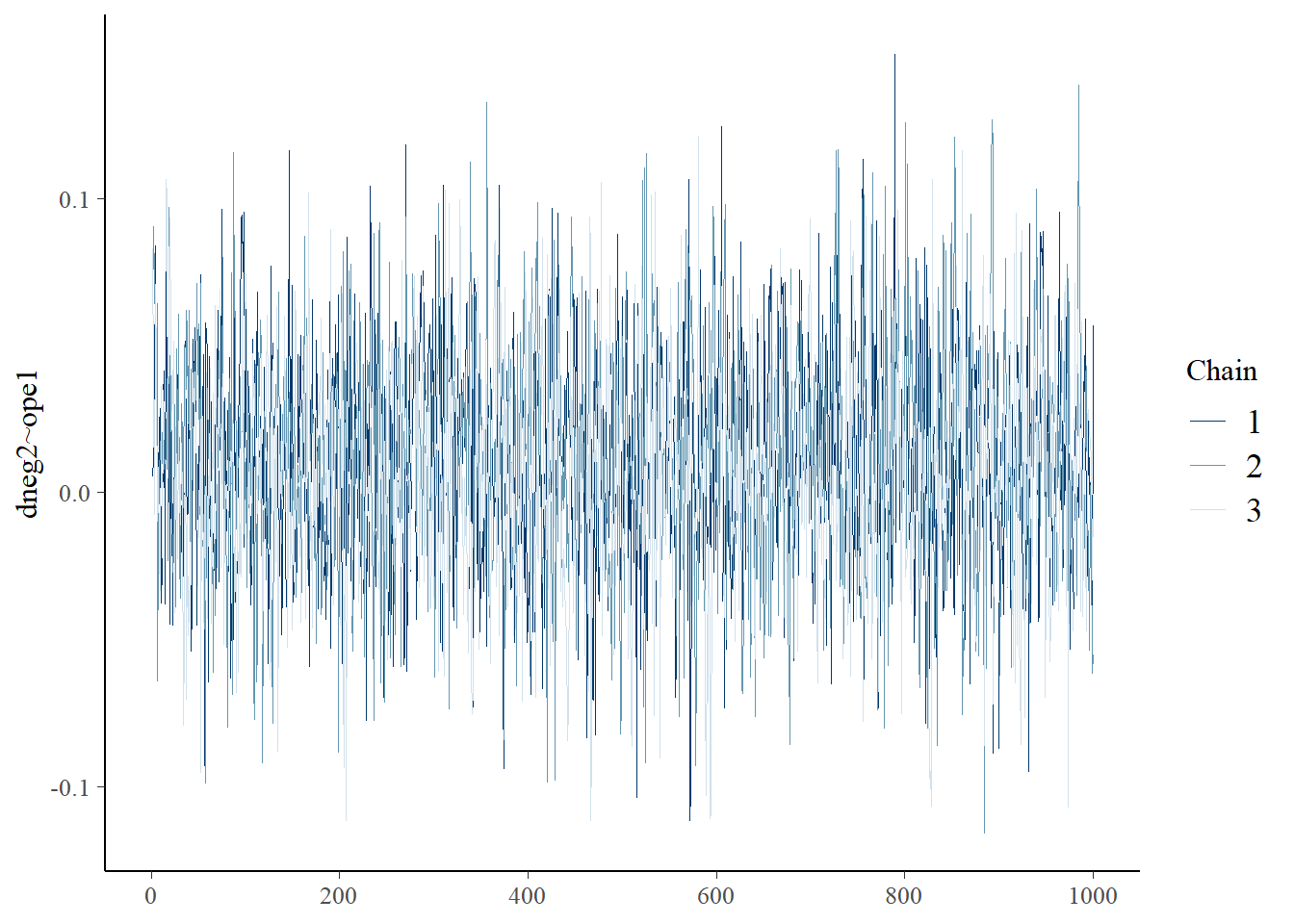


Figure 2. Traceplot of the effect of Openness to Experience on change in Negative Interactions between Wave 2 and Wave 3.


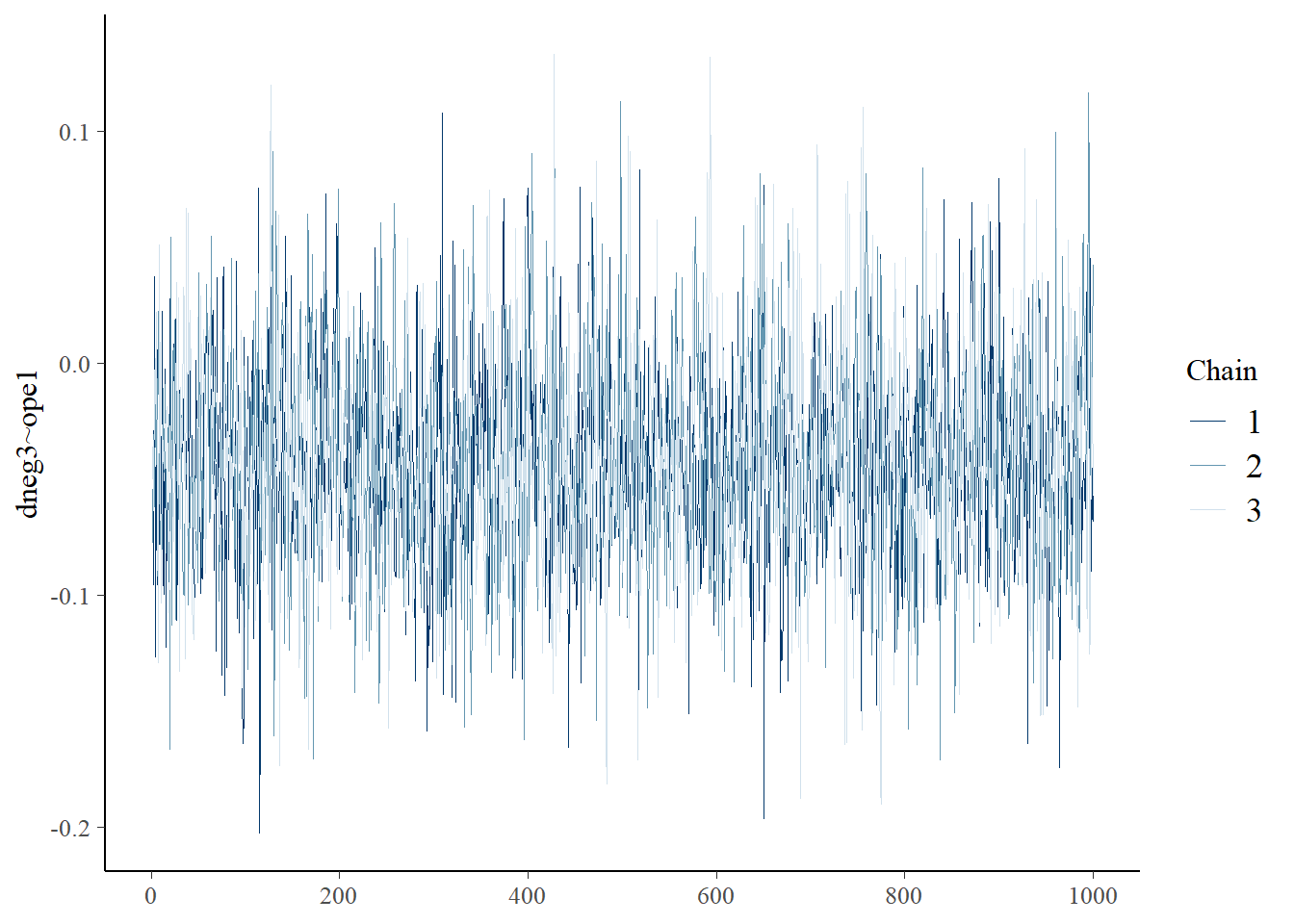


Figure 3. Traceplot of the effect of Openness to Experience on change in Negative Interactions between Wave 3 and Wave 4.


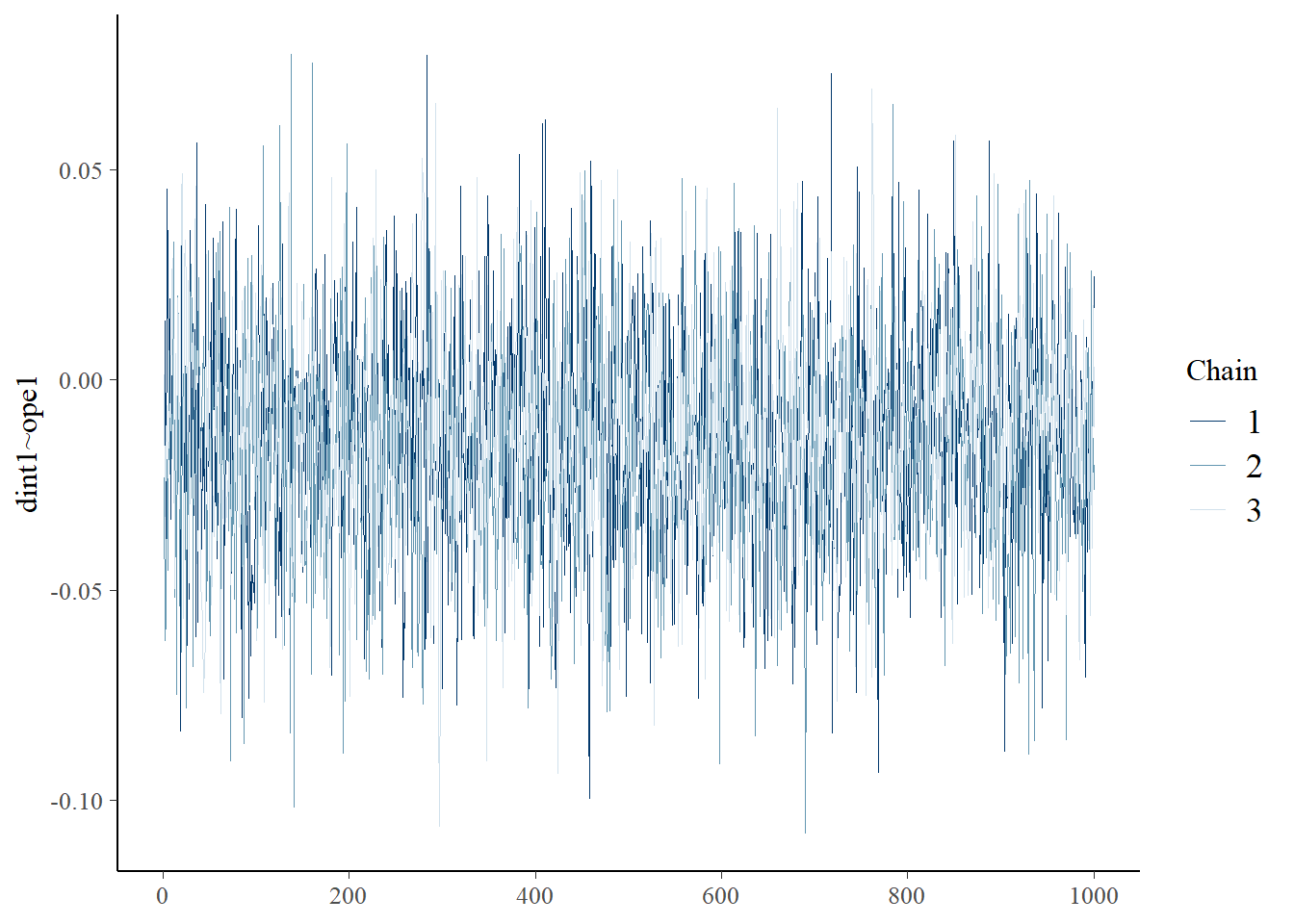


Figure 4. Traceplot of the effect of Openness to Experience on change in Mental Health Problems between Wave 1 and Wave 2.


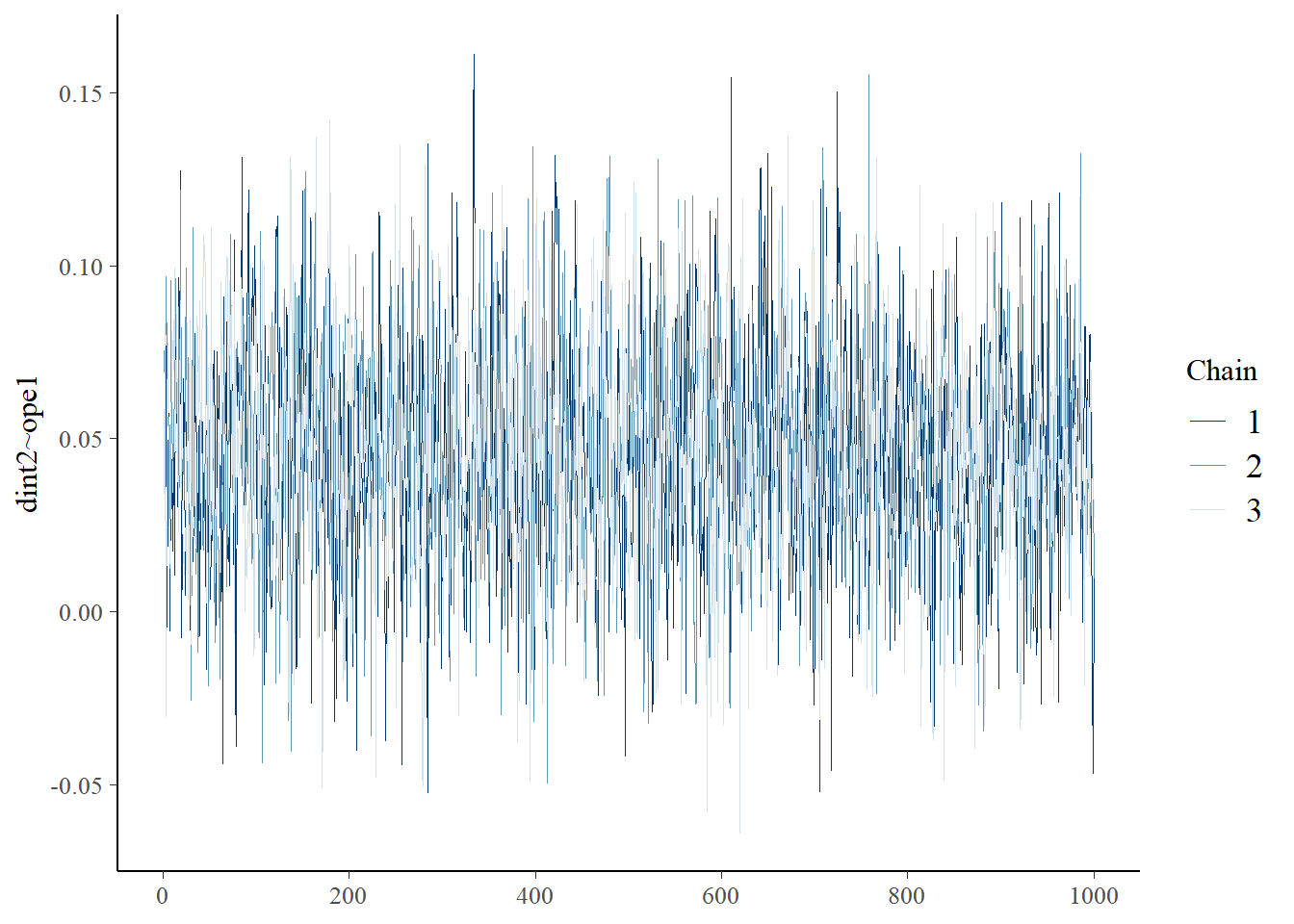


Figure 5. Traceplot of the effect of Openness to Experience on change in Mental Health Problems between Wave 2 and Wave 3.


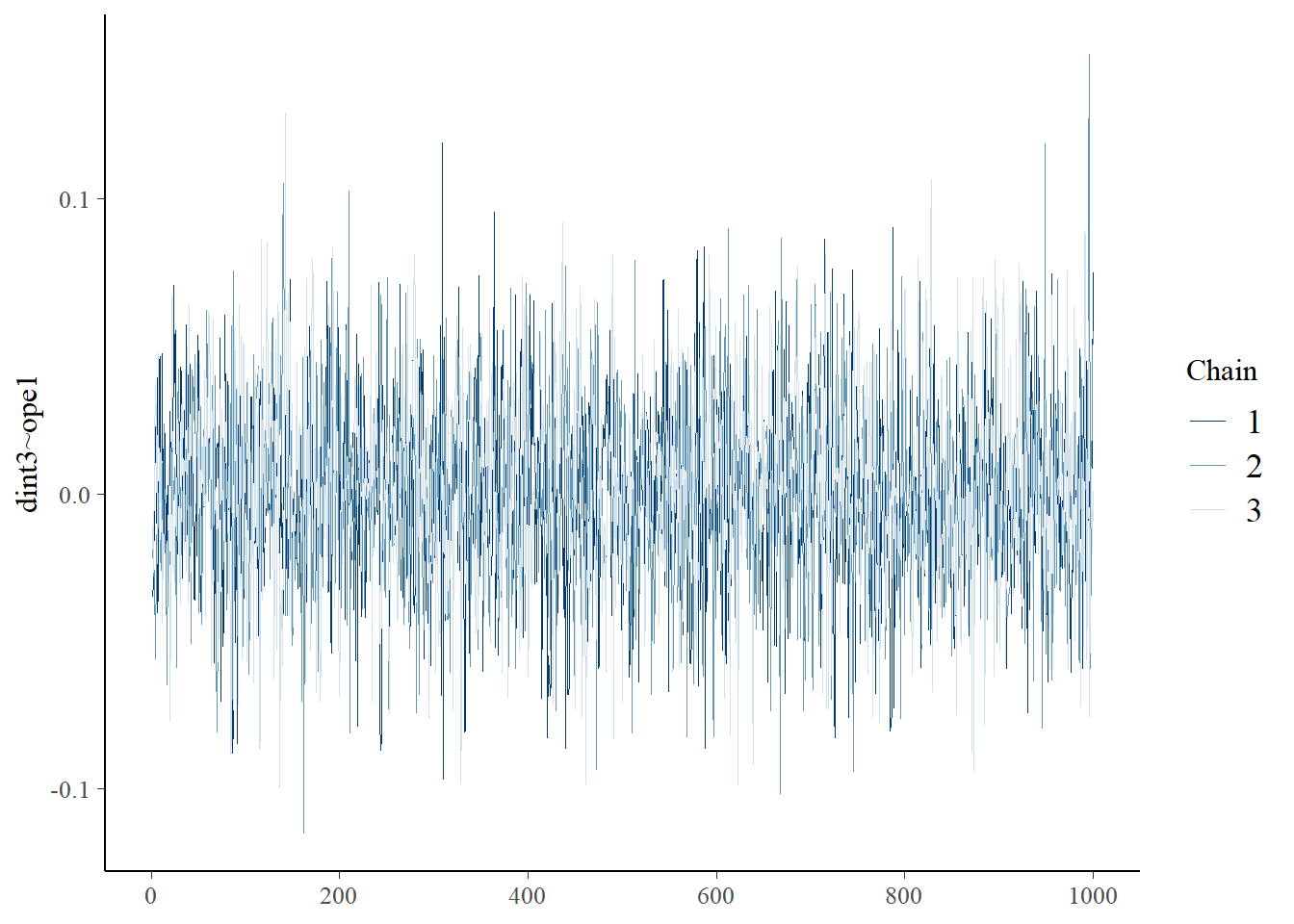


Figure 6. Traceplot of the effect of Openness to Experience on change in Mental Health Problems between Wave 3 and Wave 4.


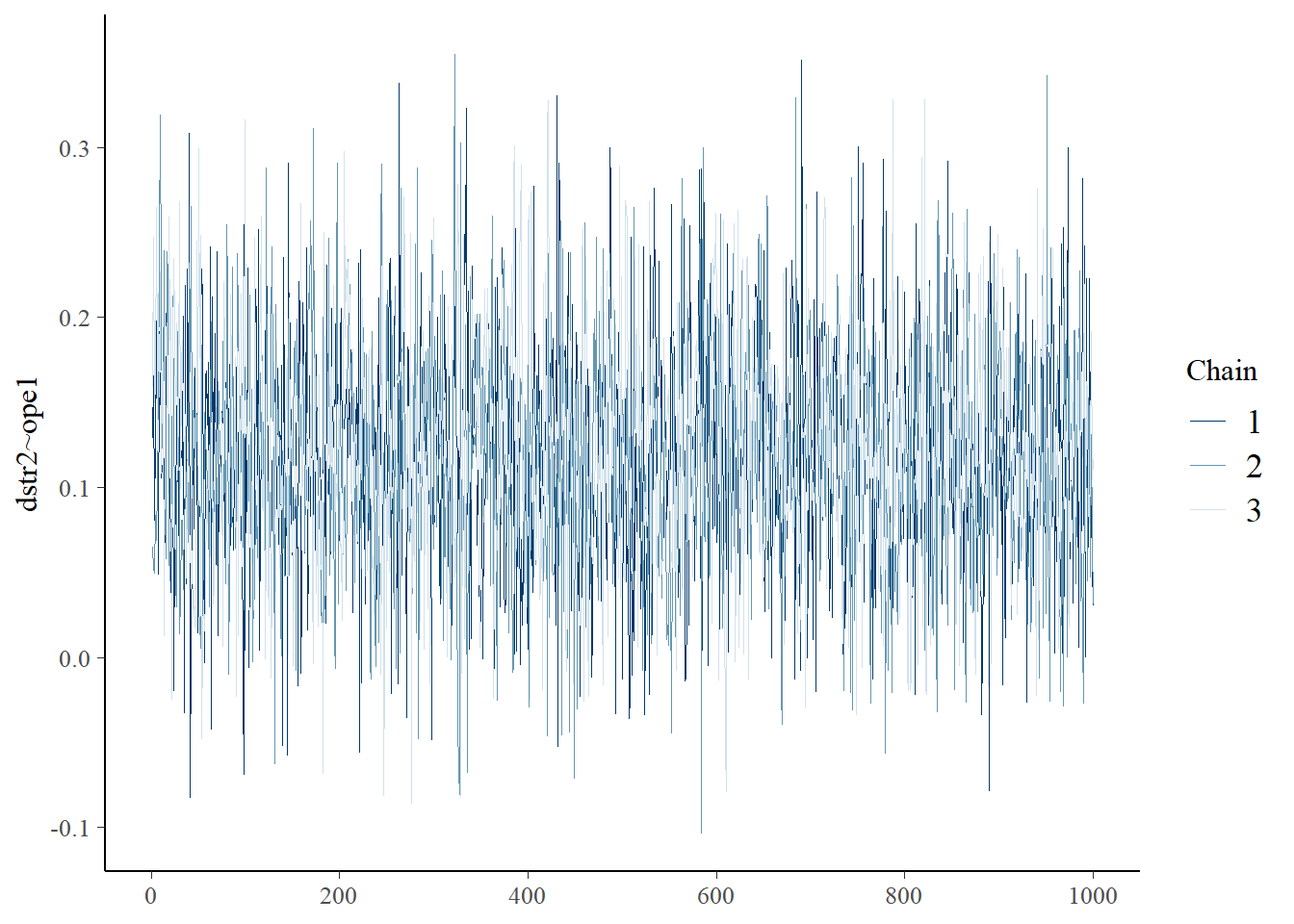


Figure 7. Traceplot of the effect of Openness to Experience on change in COVID-related Stress between Wave 2 and Wave 3.


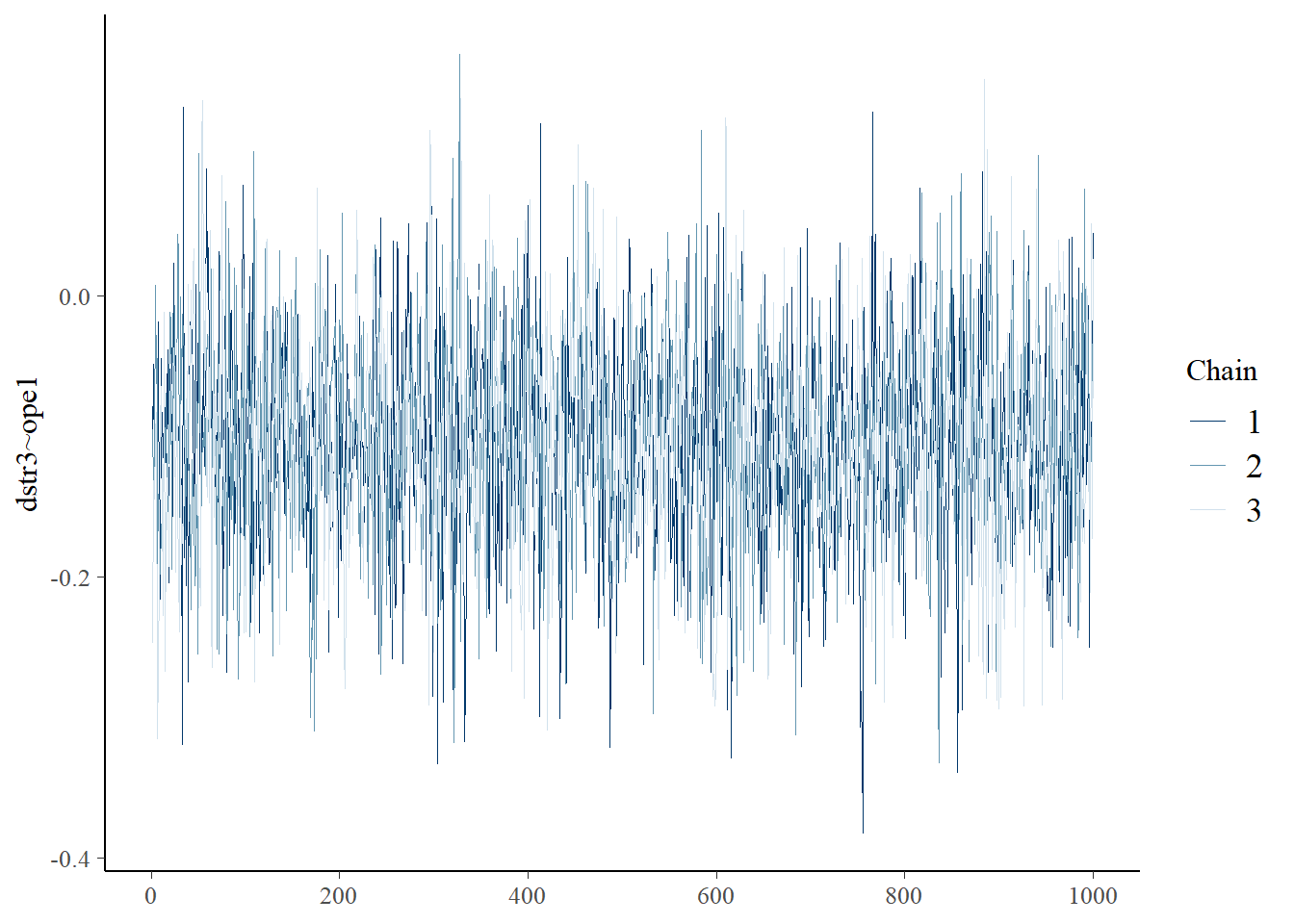


Figure 8. Traceplot of the effect of Openness to Experience on change in COVID-related Stress between Wave 3 and Wave 4.

Effects of Conscientiousness


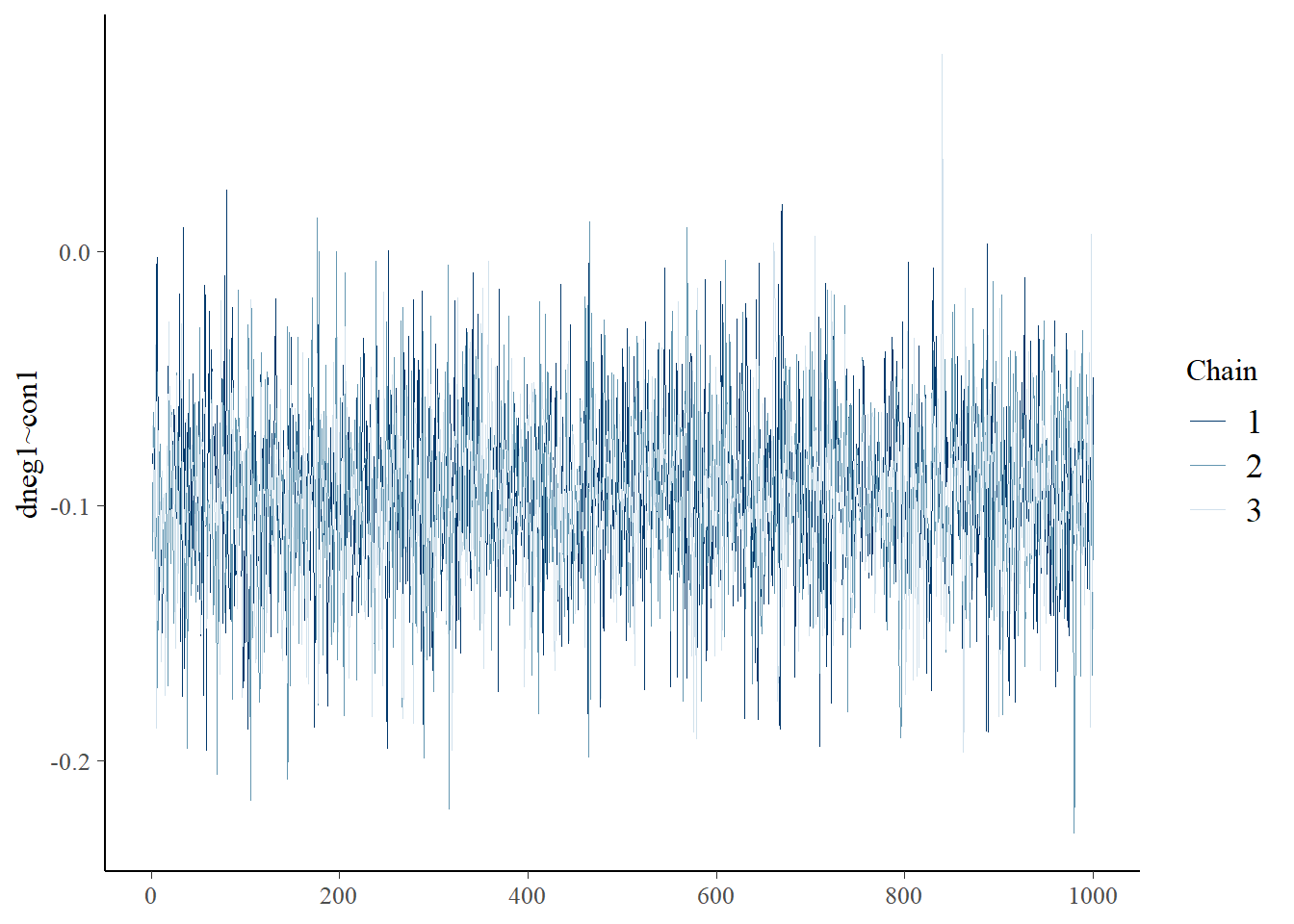


Figure 9. Traceplot of the effect of Conscientiousness on change in Negative Interactions between Wave 1 and Wave 2.


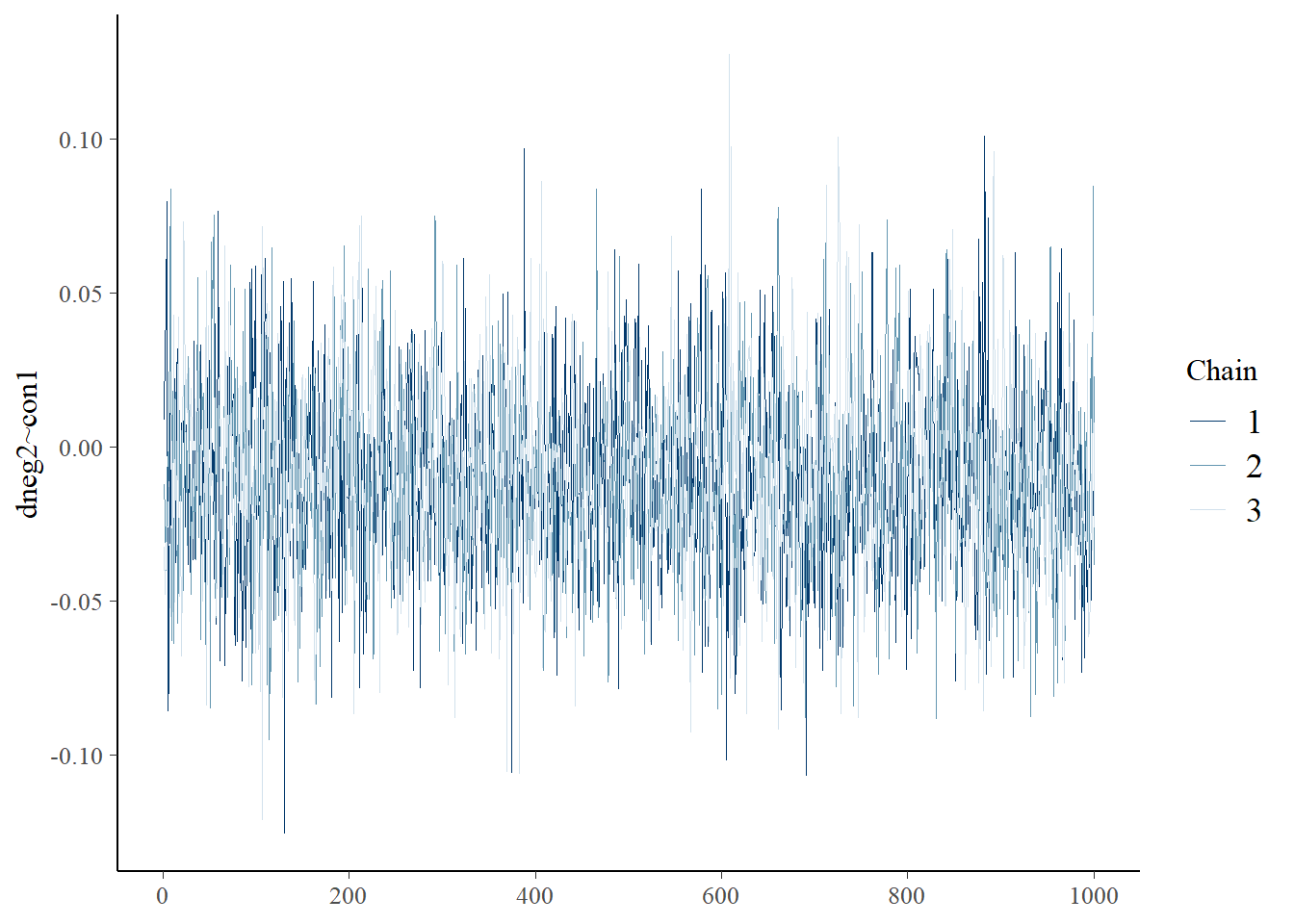


Figure 10. Traceplot of the effect of Conscientiousness on change in Negative Interactions between Wave 2and Wave 3.


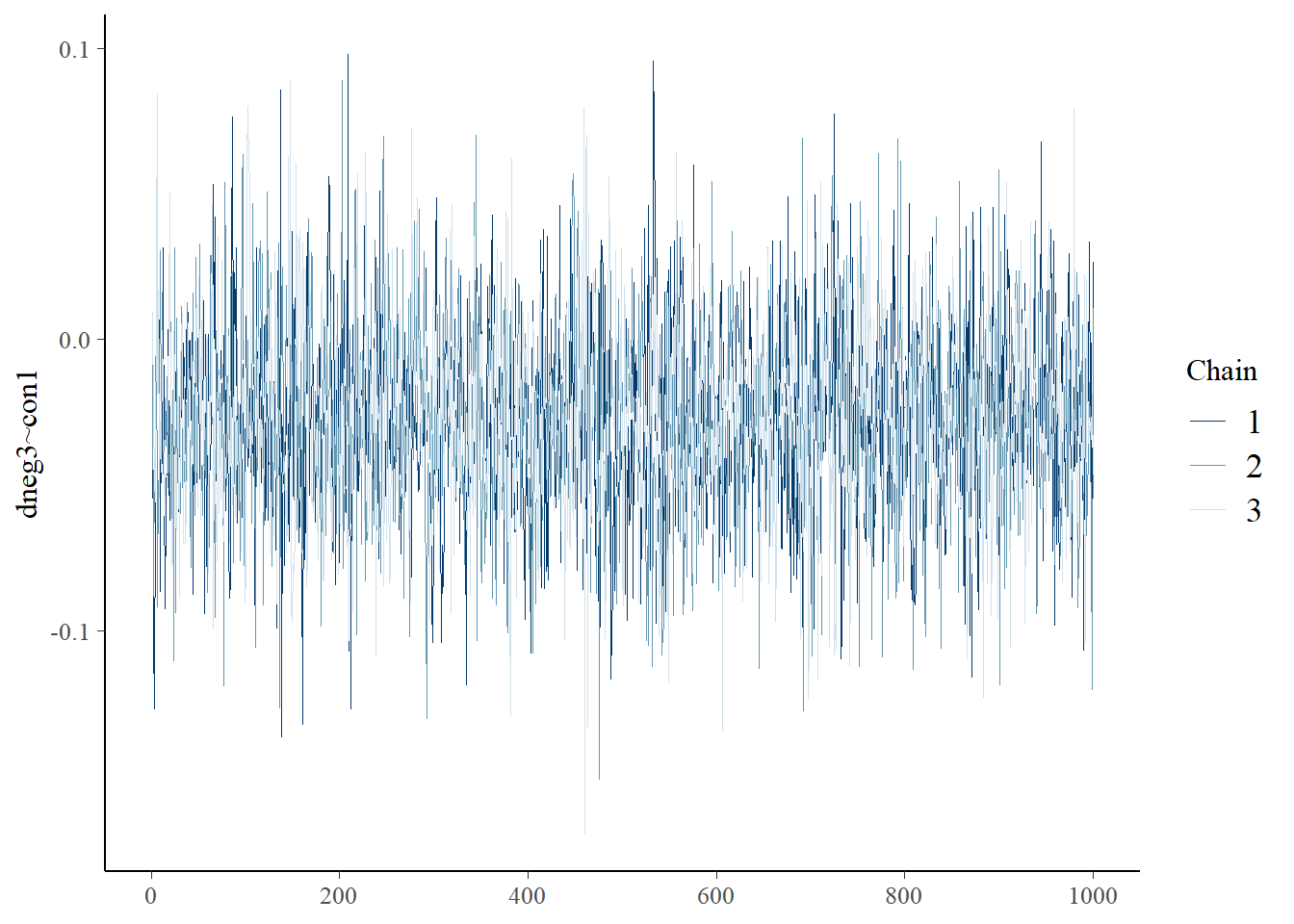


Figure 11. Traceplot of the effect of Conscientiousness on change in Negative Interactions between Wave 3 and Wave 4.


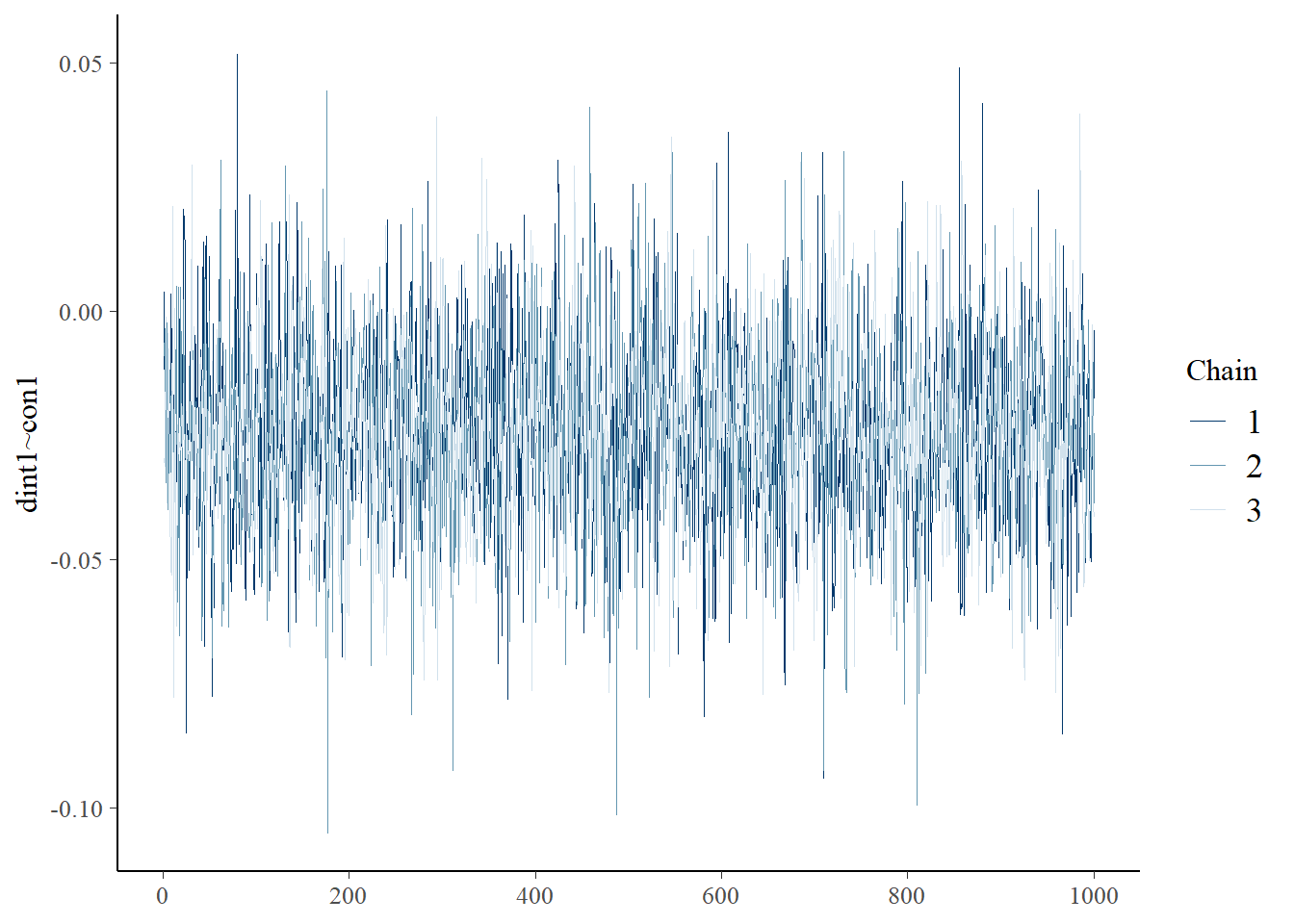


Figure 12. Traceplot of the effect of Conscientiousness on change in Mental Health Problems between Wave 1 and Wave 2.


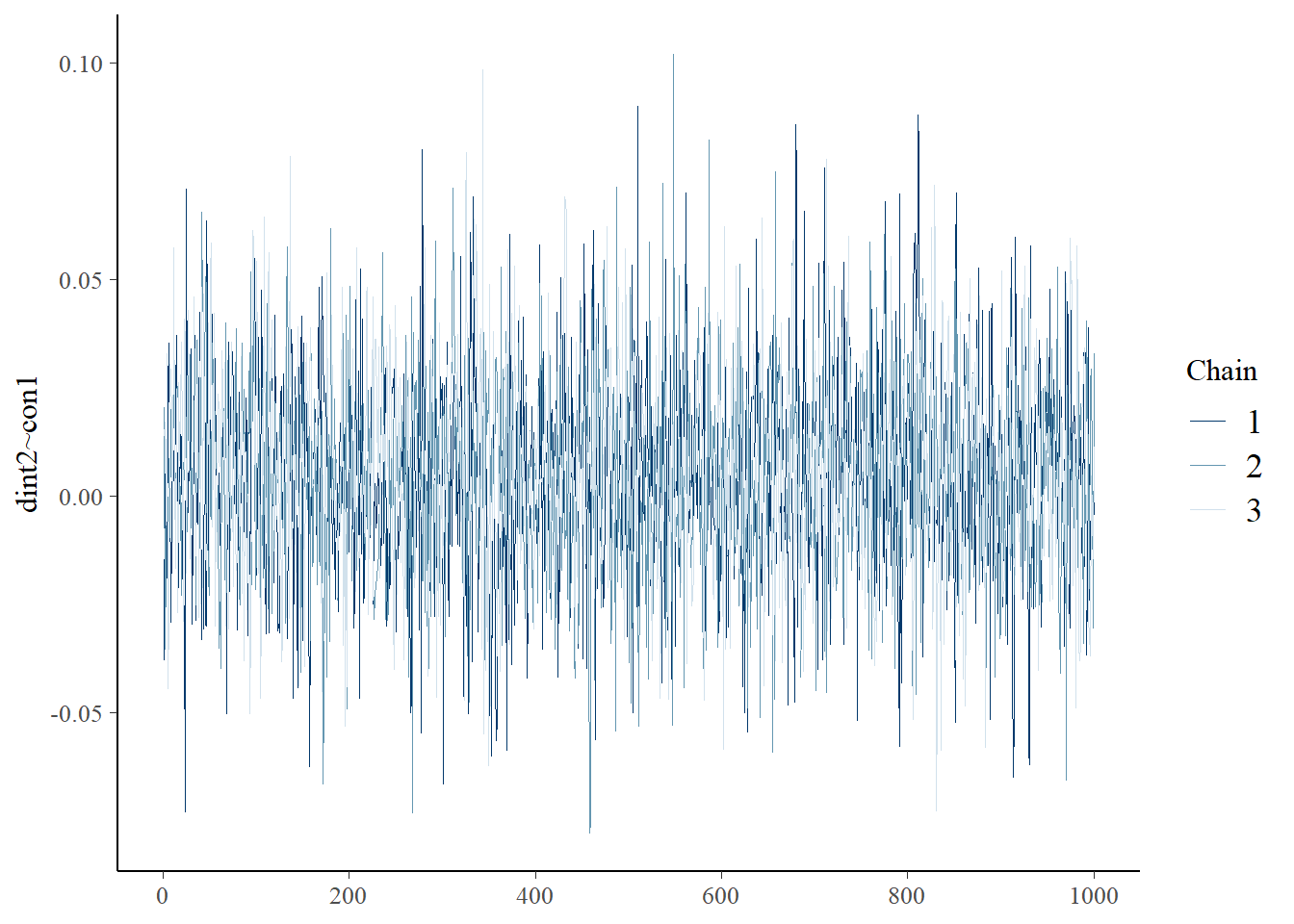


Figure 13. Traceplot of the effect of Conscientiousness on change in Mental Health Problems between Wave 2 and Wave 3.


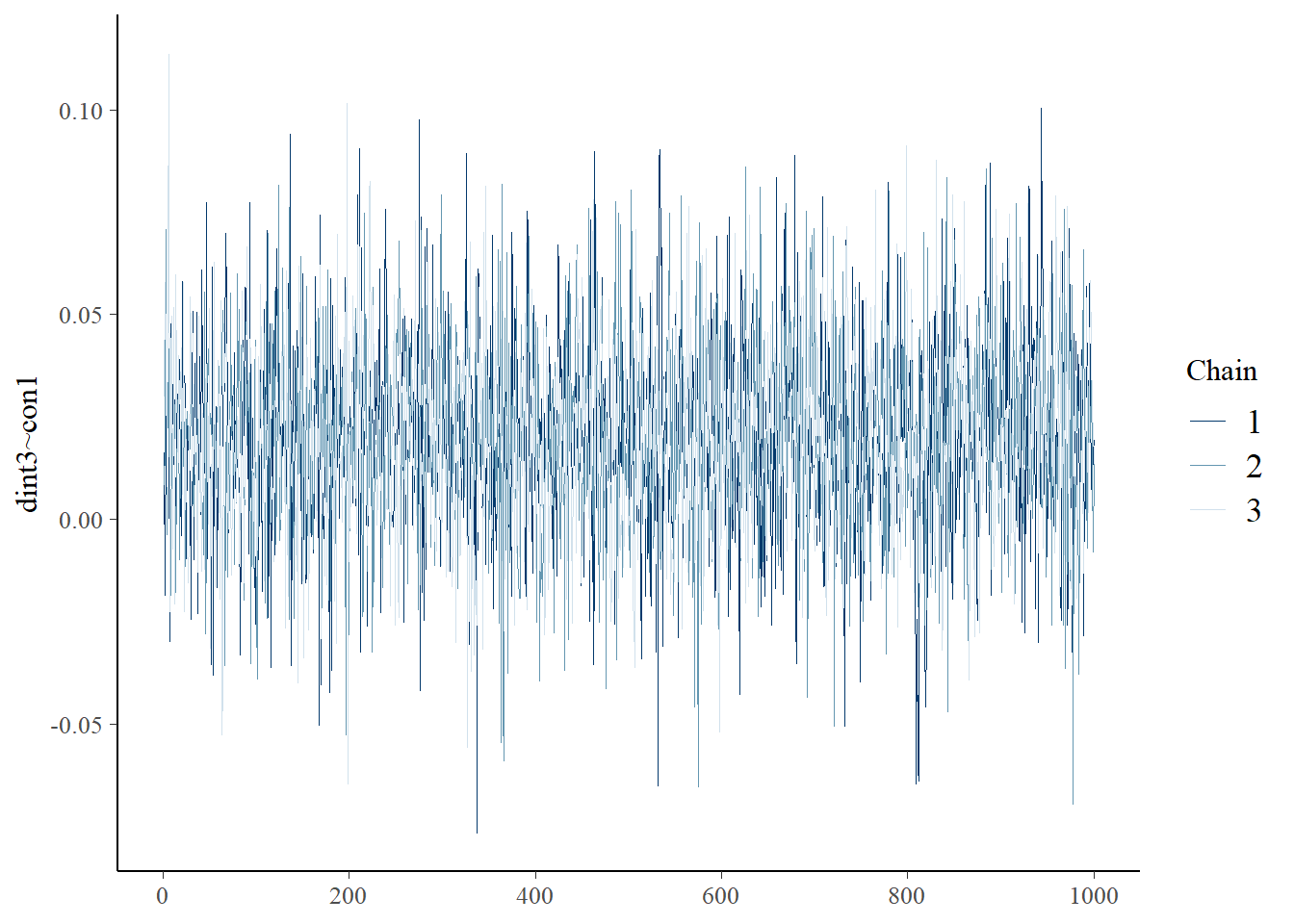


Figure 14. Traceplot of the effect of Conscientiousness on change in Mental Health Problems between Wave 3 and Wave 4.


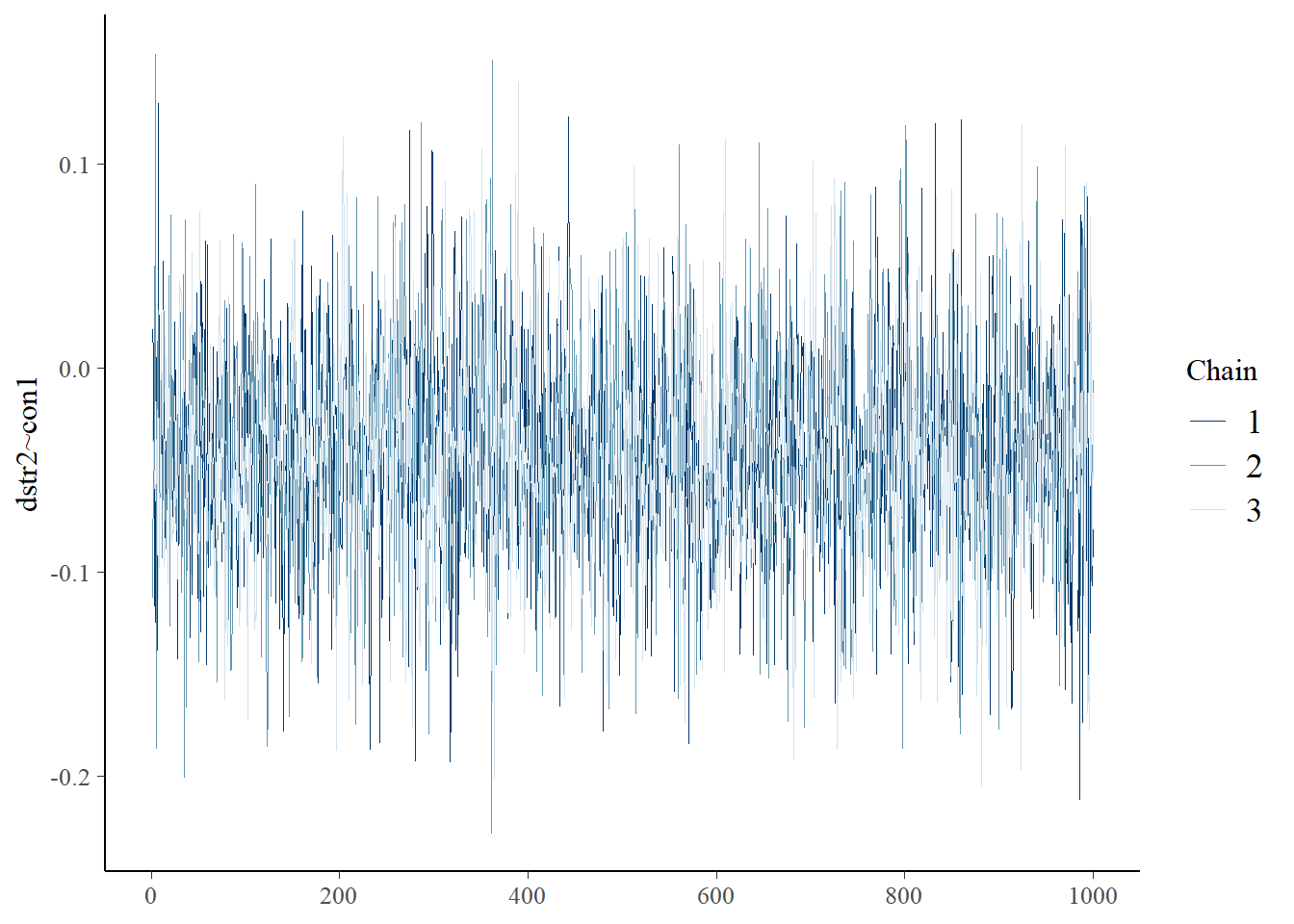


Figure 15. Traceplot of the effect of Conscientiousness on change in COVID-related Stress between Wave 2 and Wave 3.


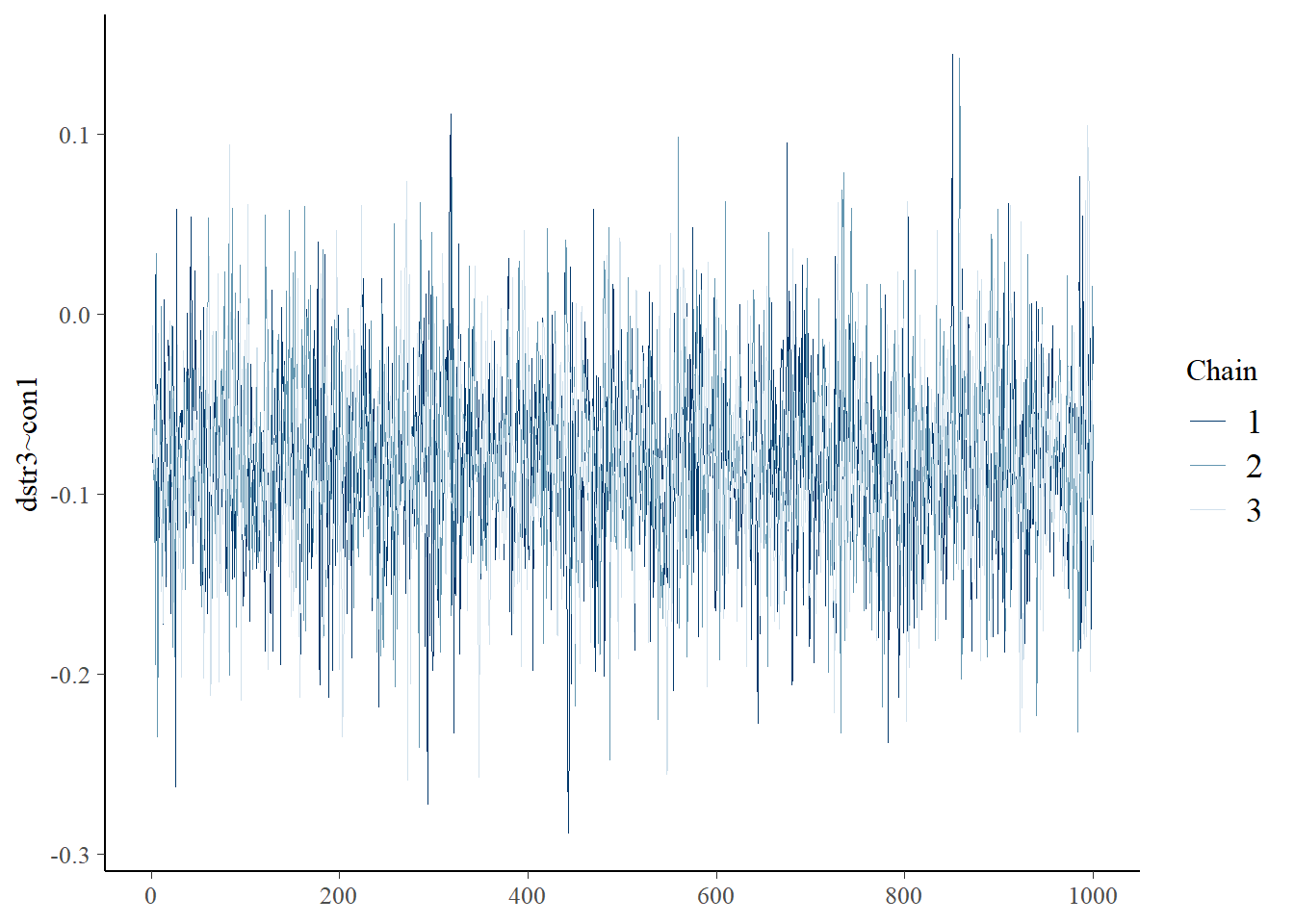


Figure 16. Traceplot of the effect of Conscientiousness on change in COVID-related Stress between Wave 3 and Wave 4.

Effects of Extraversion
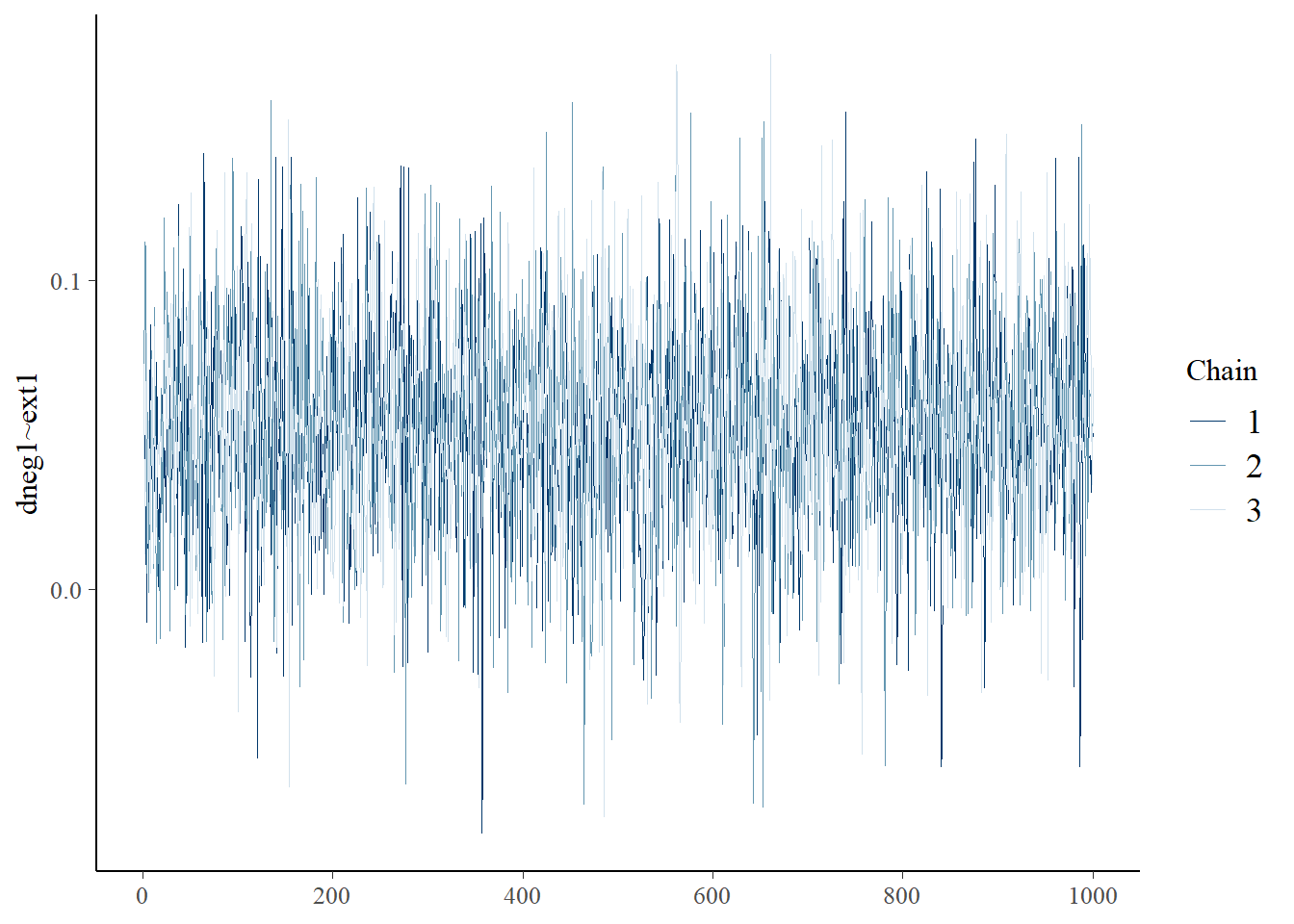


Figure 17. Traceplot of the effect of Extraversion on change in Negative Interactions between Wave 1 and Wave 2.


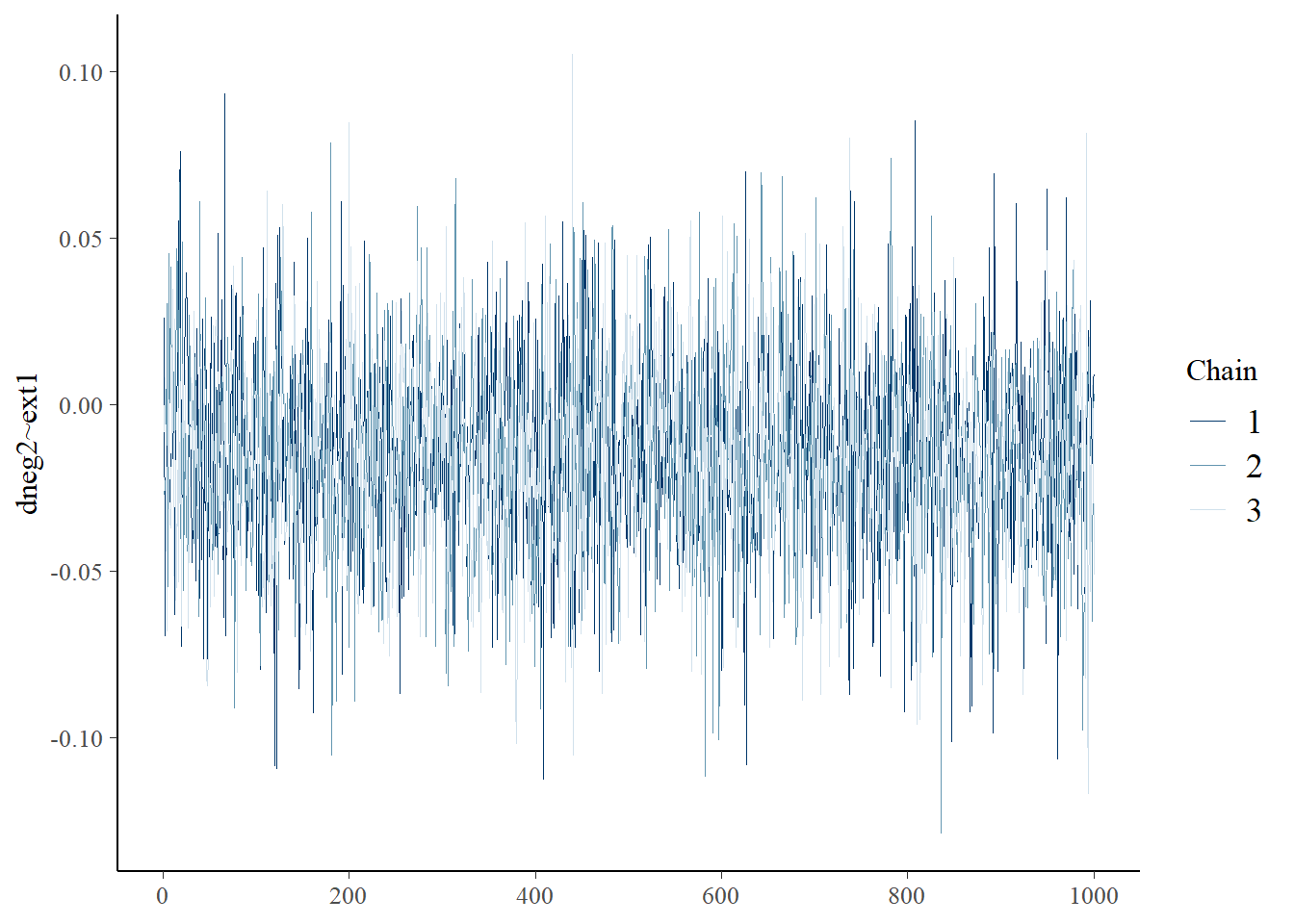


Figure 18. Traceplot of the effect of Extraversion on change in Negative Interactions between Wave 2 and Wave 3.


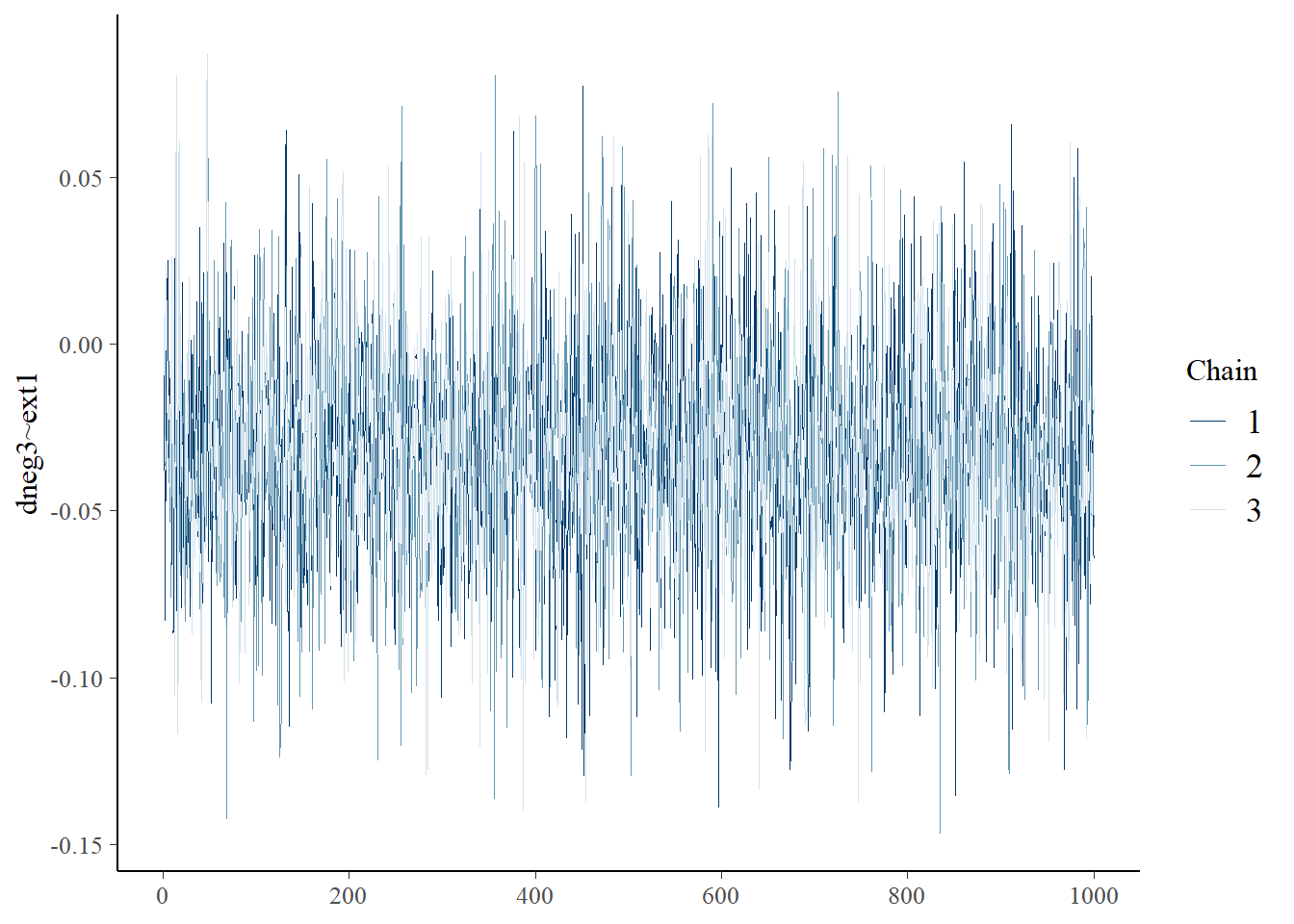


Figure 19. Traceplot of the effect of Extraversion on change in Negative Interactions between Wave 3 and Wave 4.


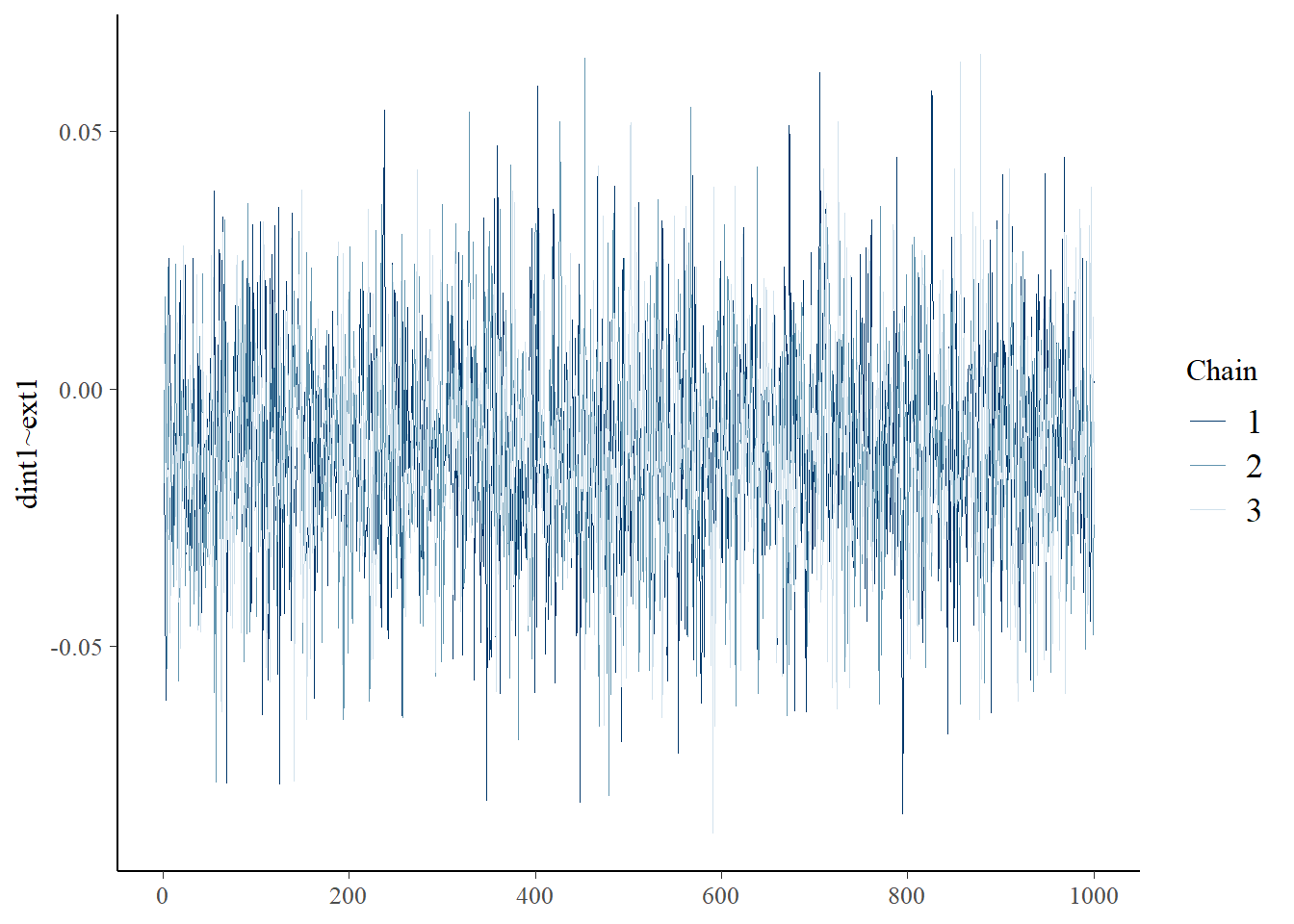


Figure 20. Traceplot of the effect of Extraversion on change in Mental Health Problems between Wave 1 and Wave 2.


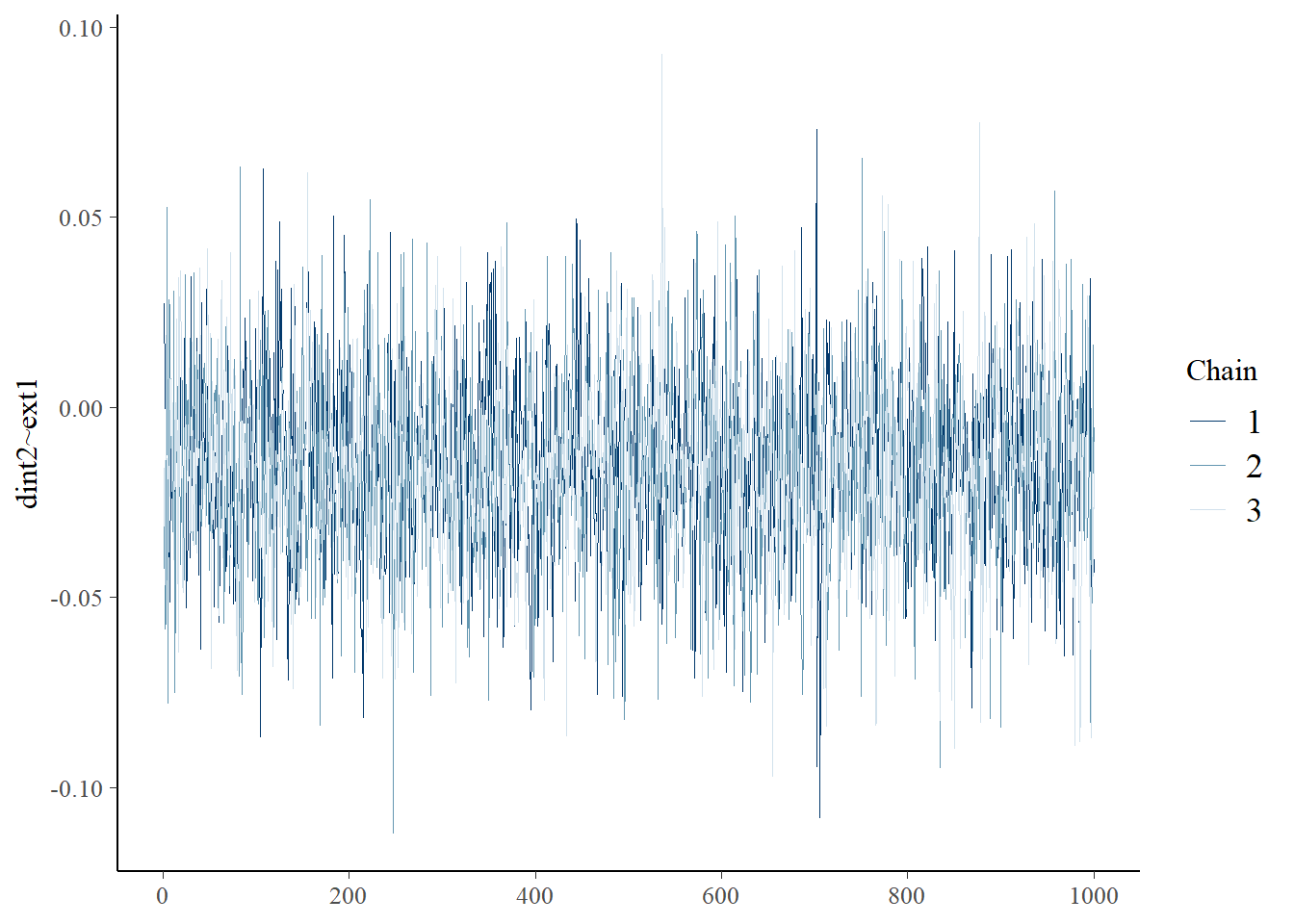


Figure 21. Traceplot of the effect of Extraversion on change in Mental Health Problems between Wave 2 and Wave 3.


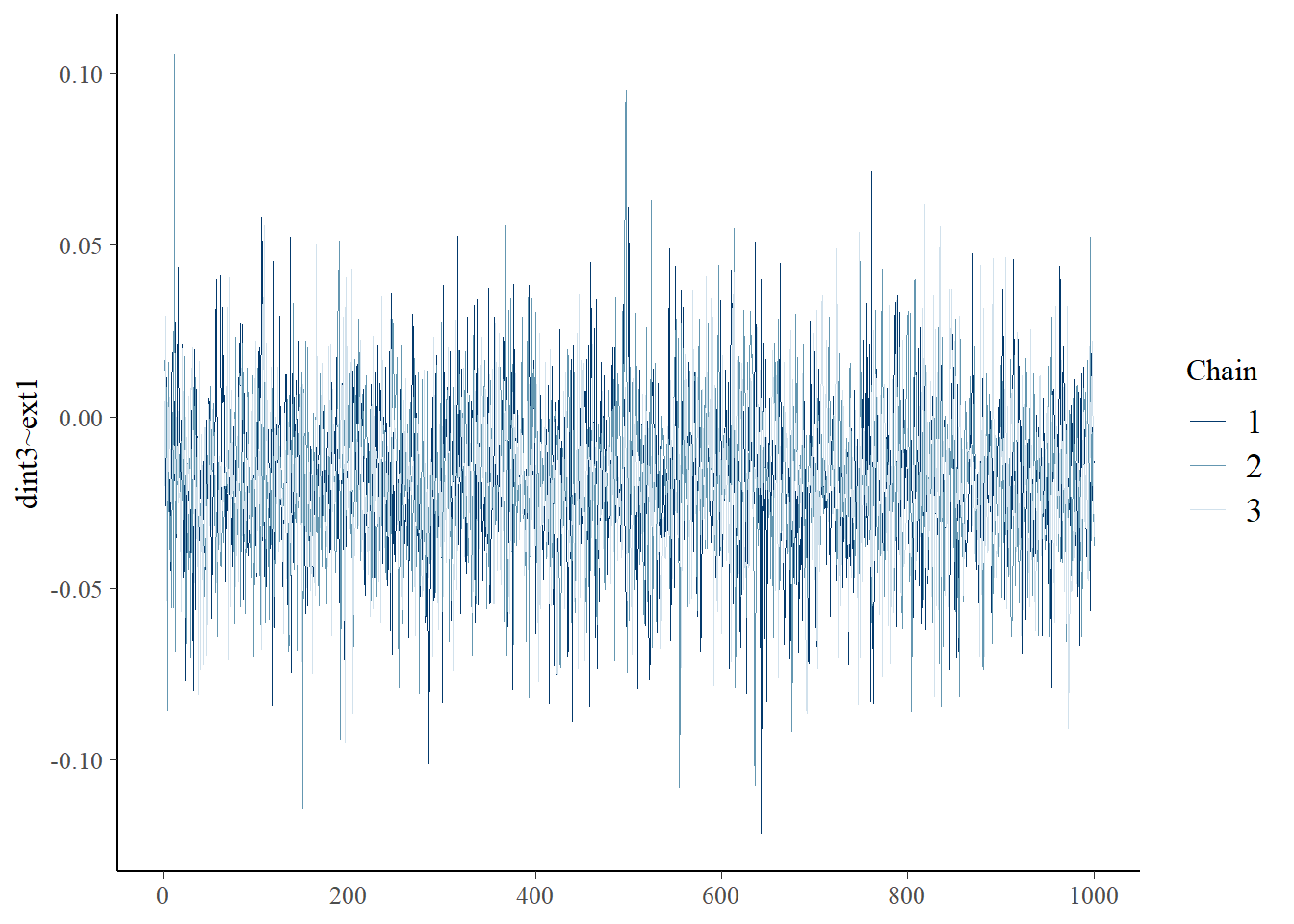


Figure 22. Traceplot of the effect of Extraversion on change in Mental Health Problems between Wave 3 and Wave 4.


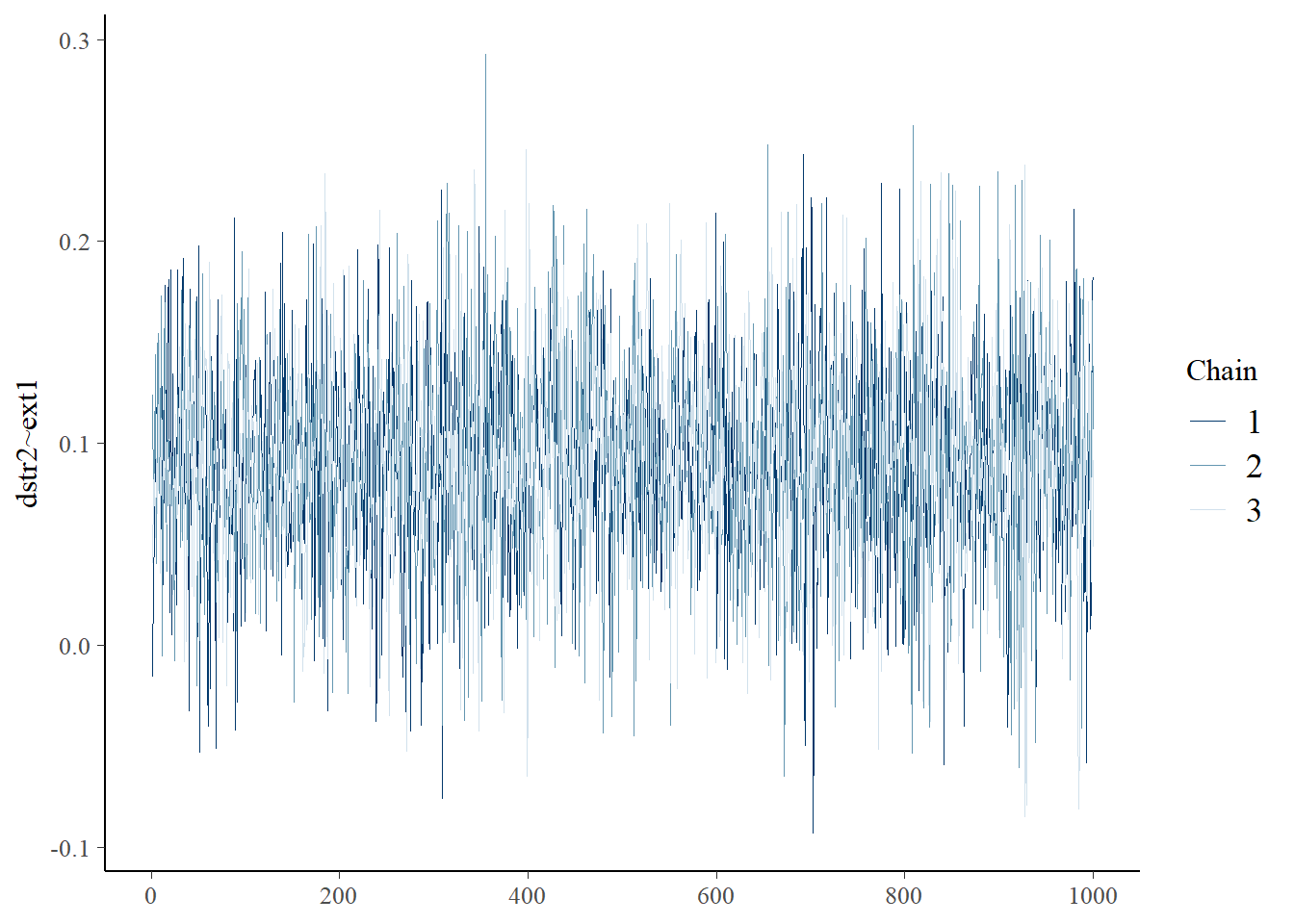


Figure 23. Traceplot of the effect of Extraversion on change in COVID-related Stress between Wave 2 and Wave 3.


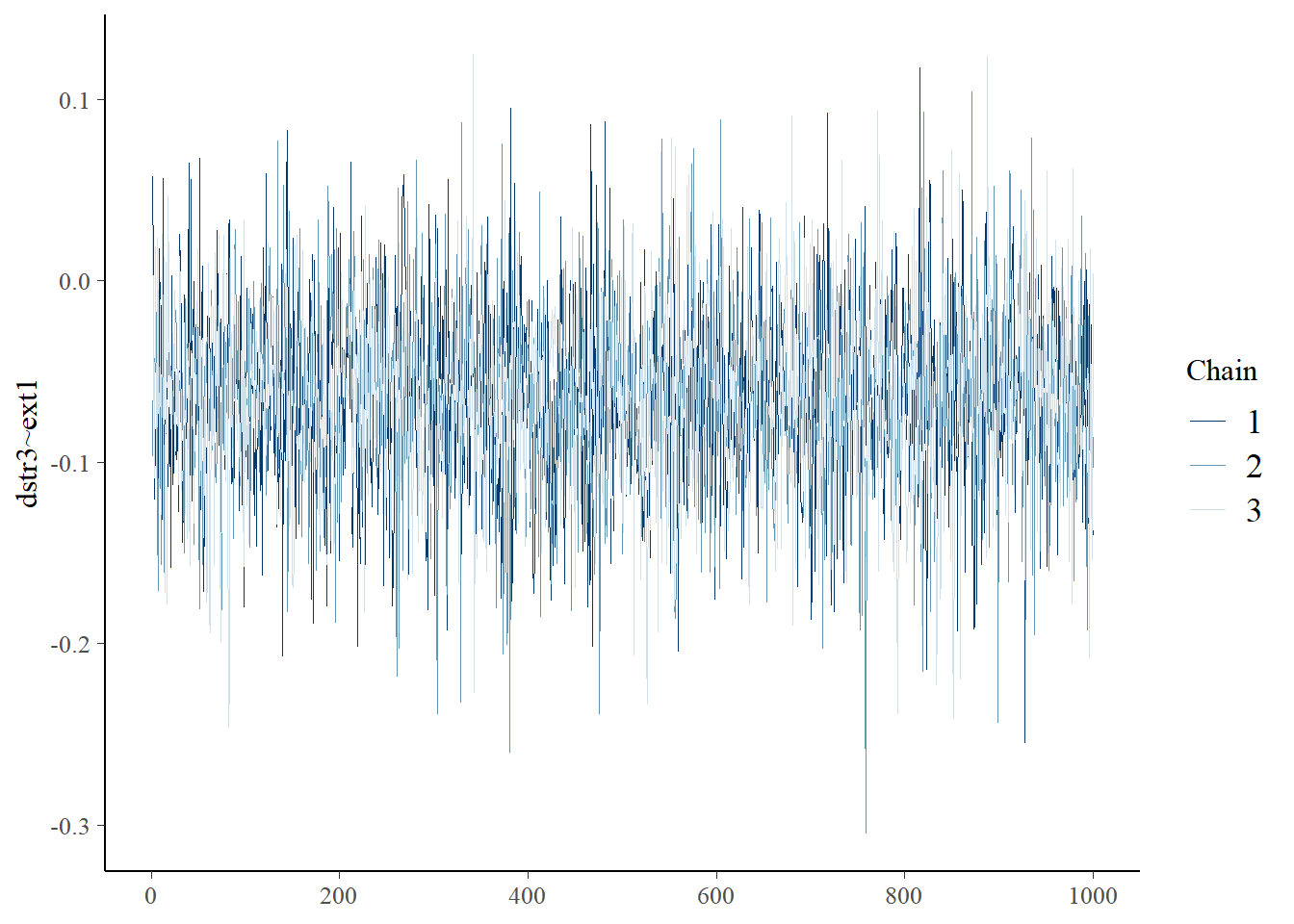


Figure 24. Traceplot of the effect of Extraversion on change in COVID-related Stress between Wave 3 and Wave 4.

Effects of Agreeableness
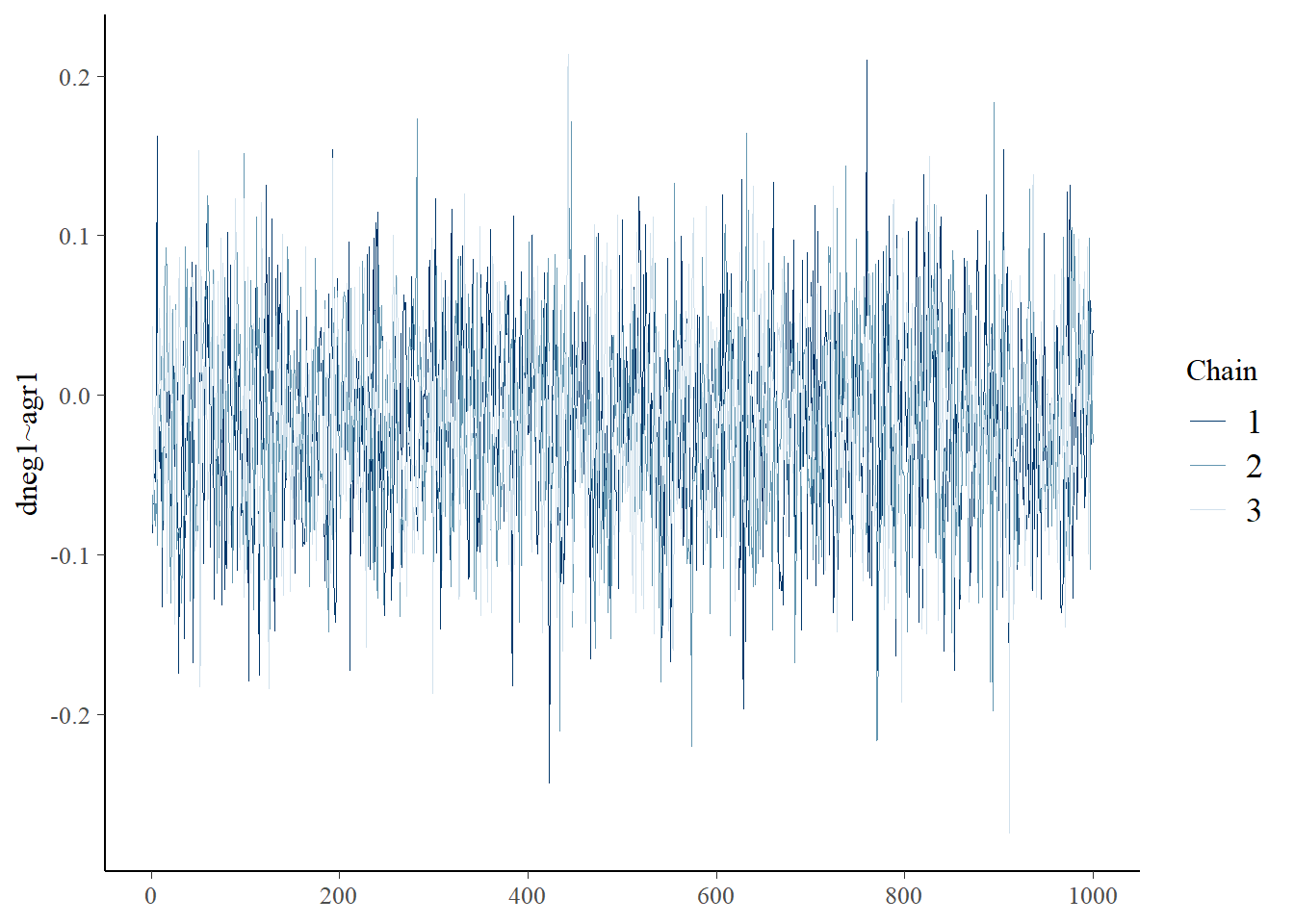


Figure 25. Traceplot of the effect of Agreeableness on change in Negative Interactions between Wave 1 and Wave 2.


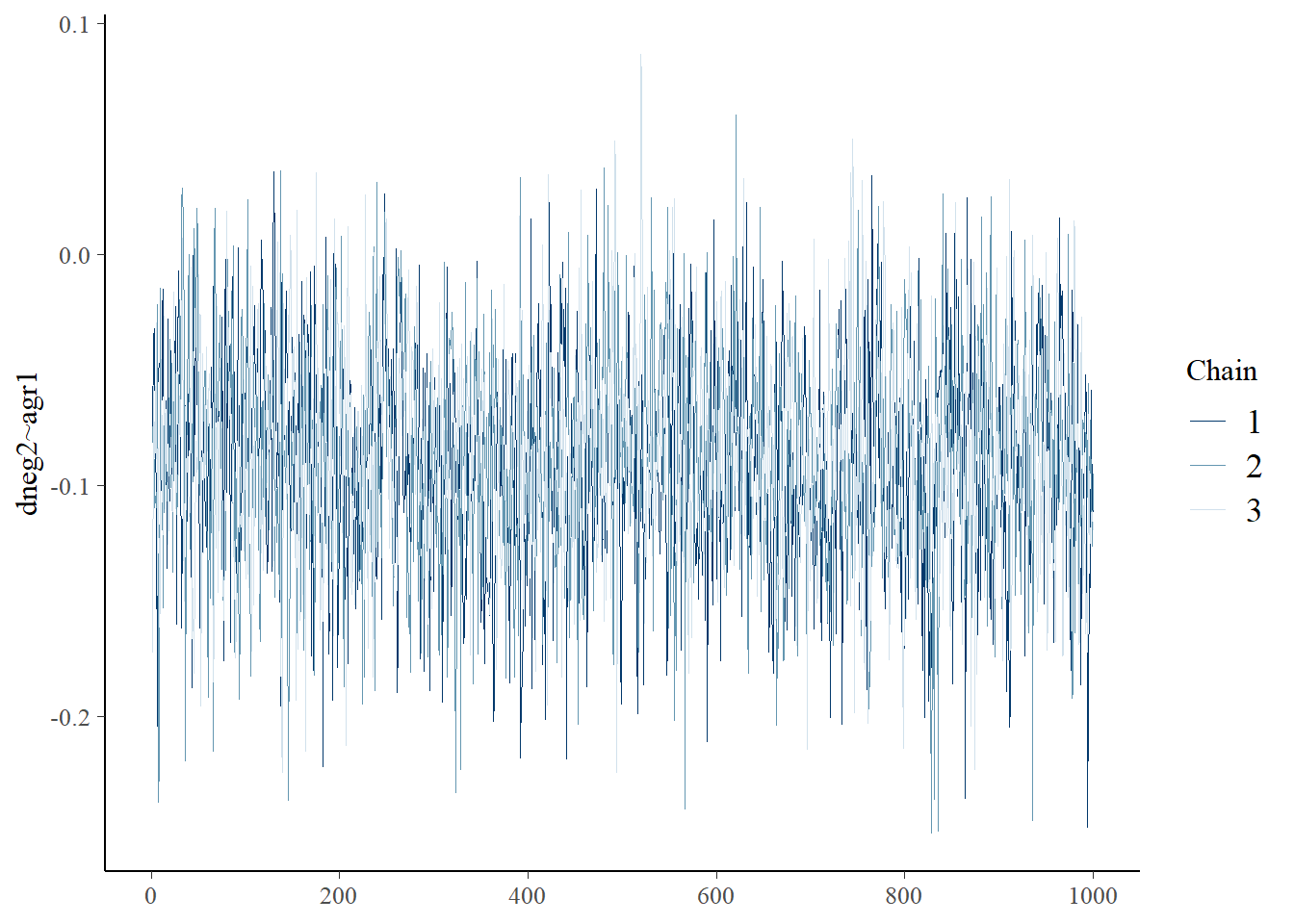


Figure 26. Traceplot of the effect of Agreeableness on change in Negative Interactions between Wave 2 and Wave 3.


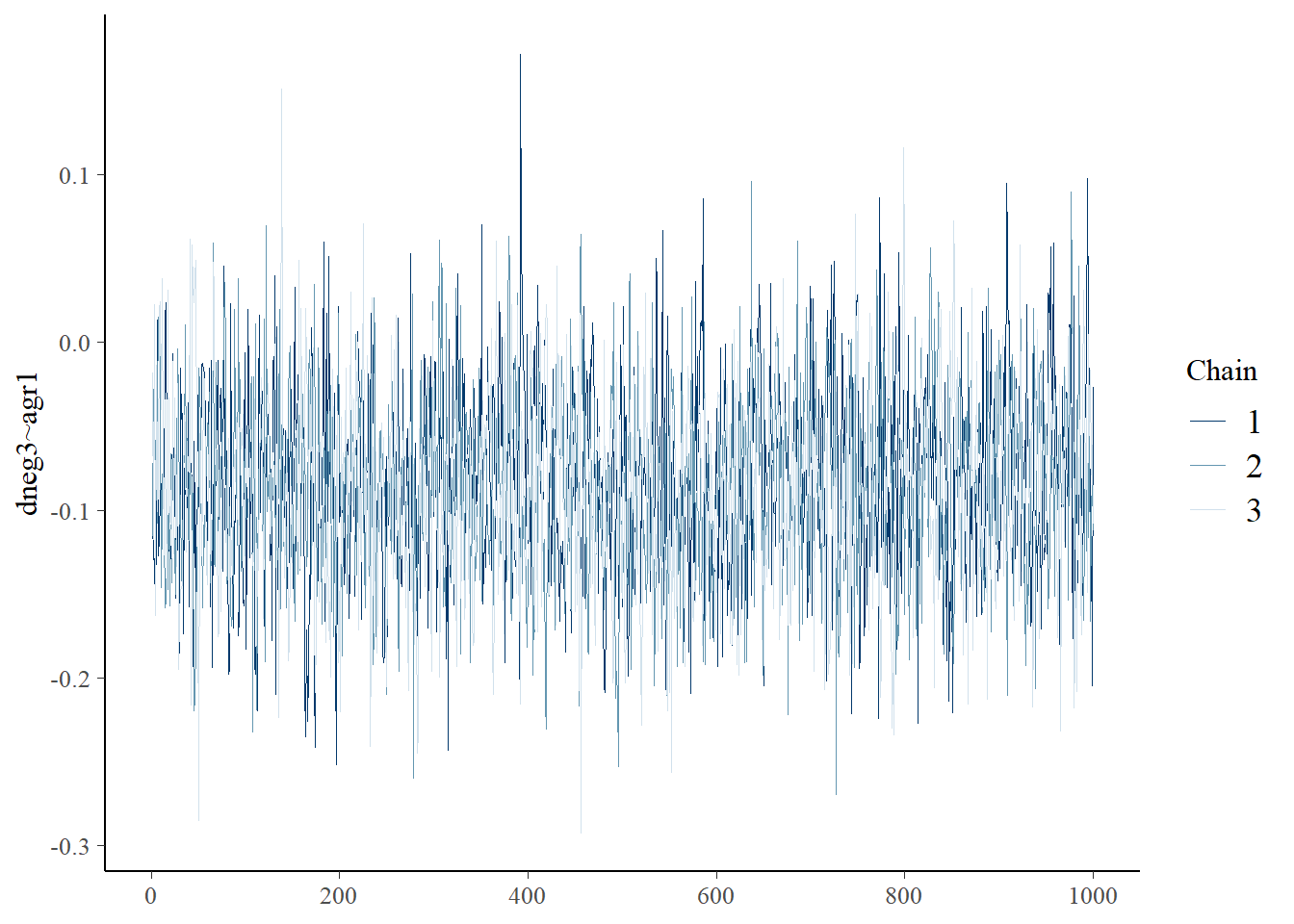


Figure 27. Traceplot of the effect of Agreeableness on change in Negative Interactions between Wave 3 and Wave 4.


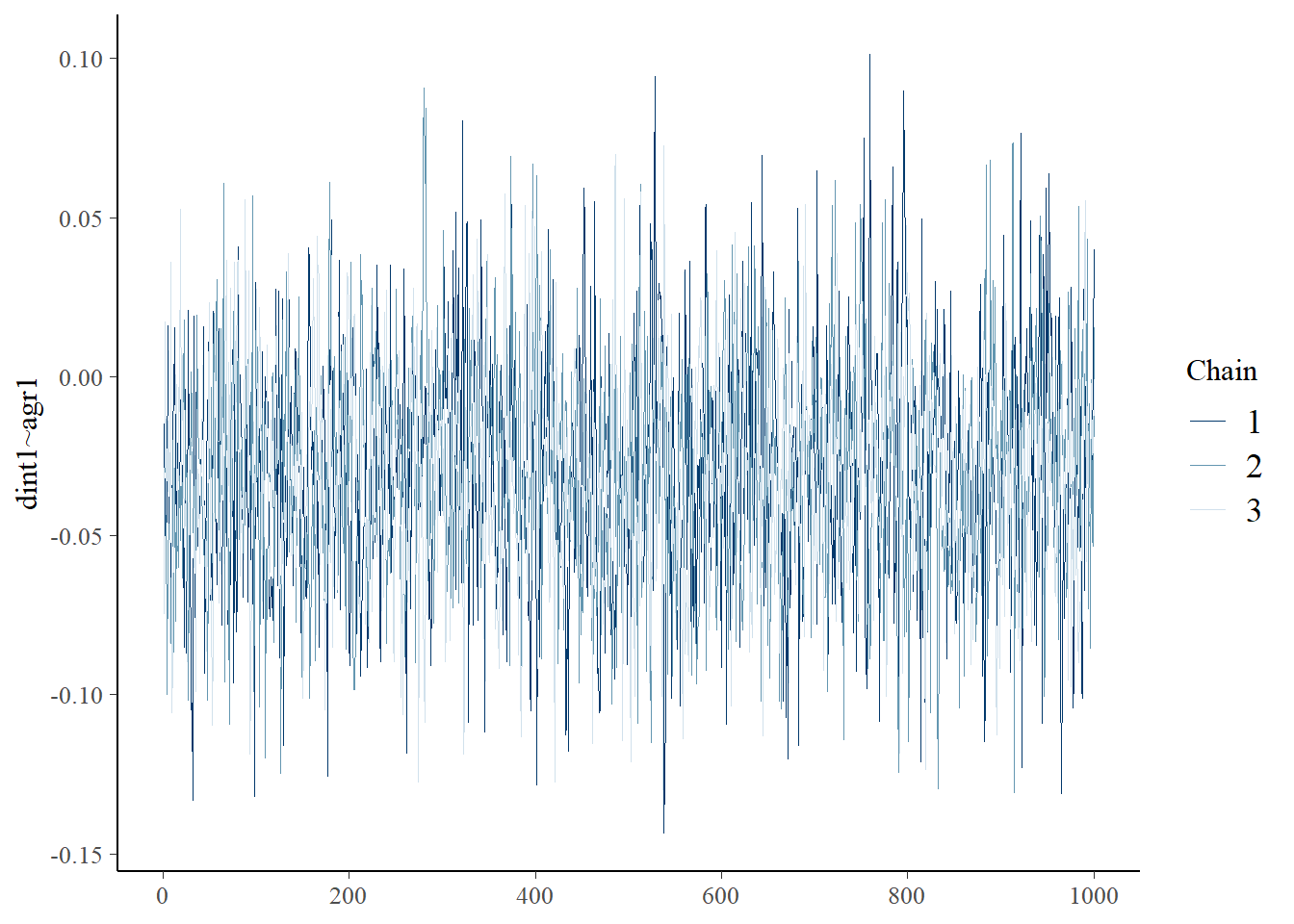


Figure 28. Traceplot of the effect of Agreeableness on change in Mental Health Problems between Wave 1 and Wave 2.


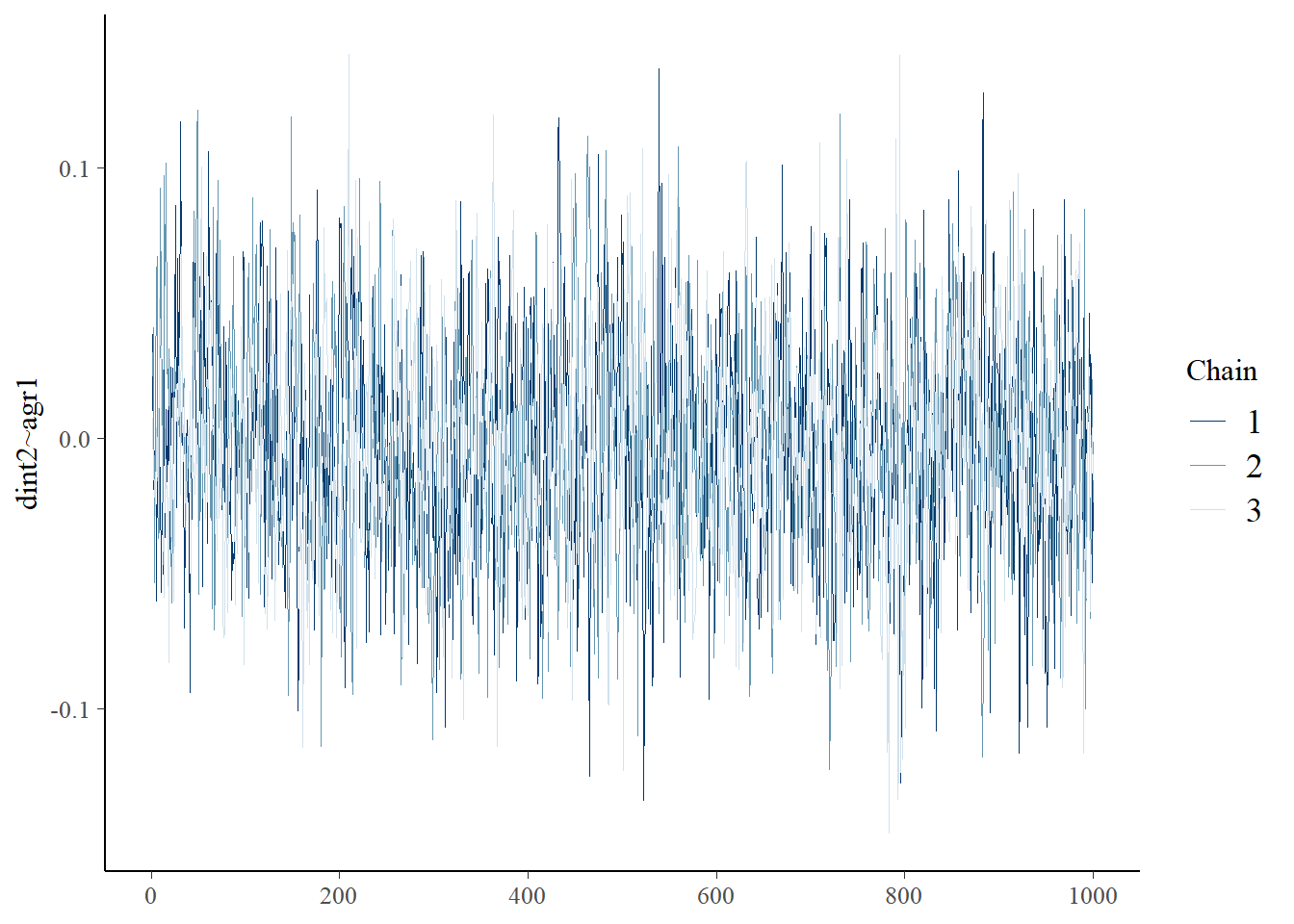


Figure 29. Traceplot of the effect of Agreeableness on change in Mental Health Problems between Wave 2 and Wave 3.


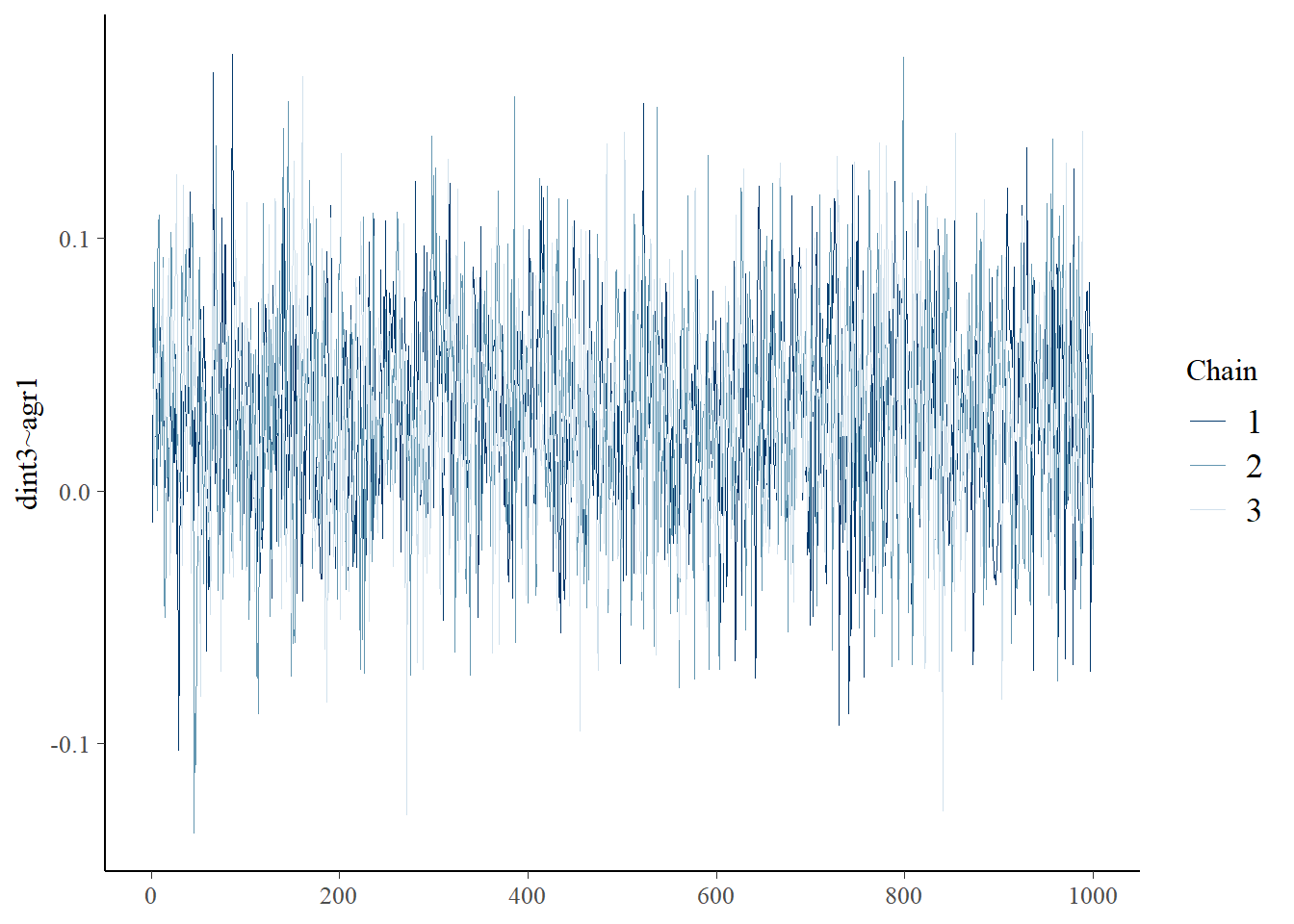


Figure 30. Traceplot of the effect of Agreeableness on change in Mental Health Problems between Wave 3 and Wave 4.


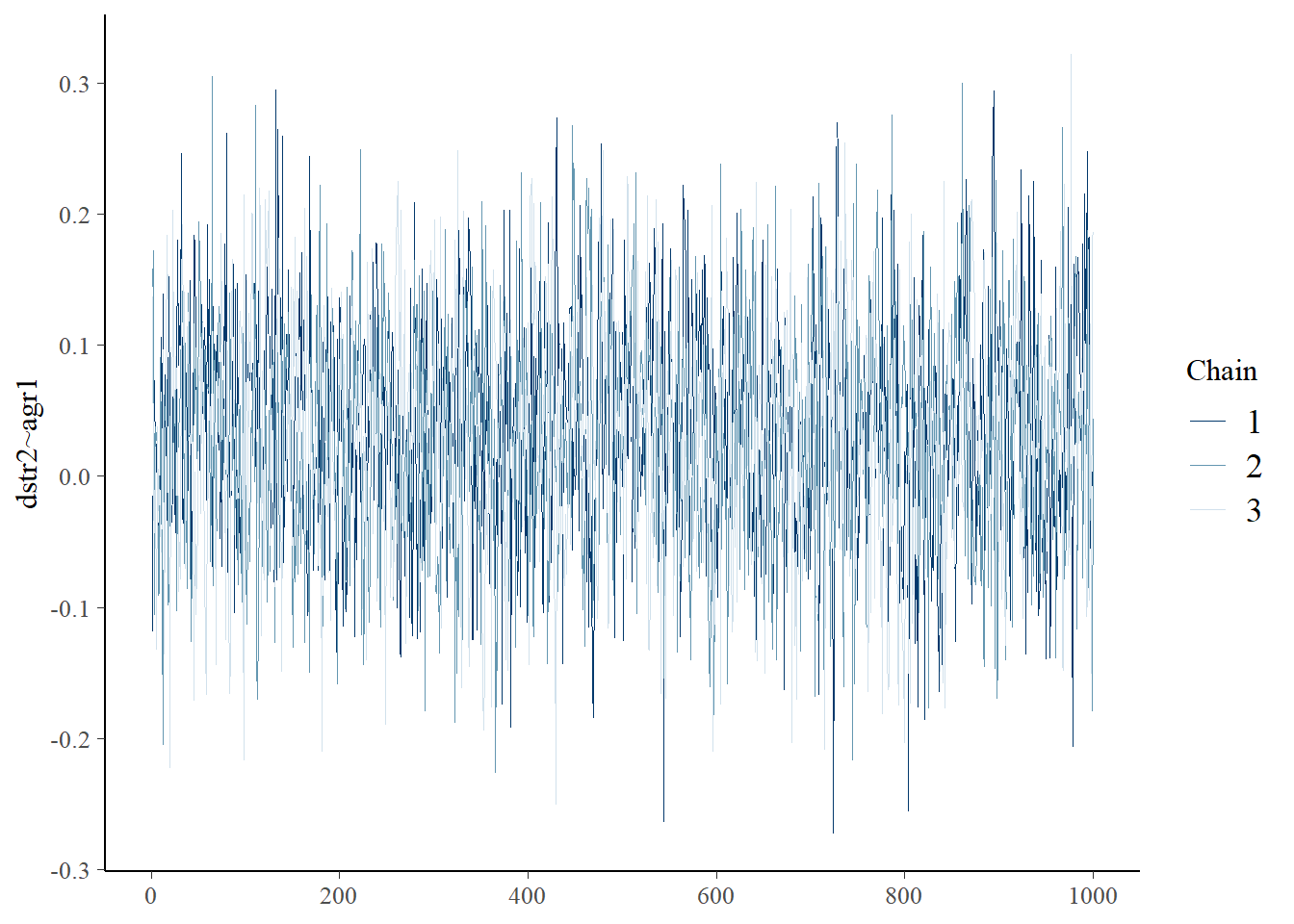


Figure 31. Traceplot of the effect of Agreeableness on change in COVID-related Stress between Wave 2 and Wave 3.


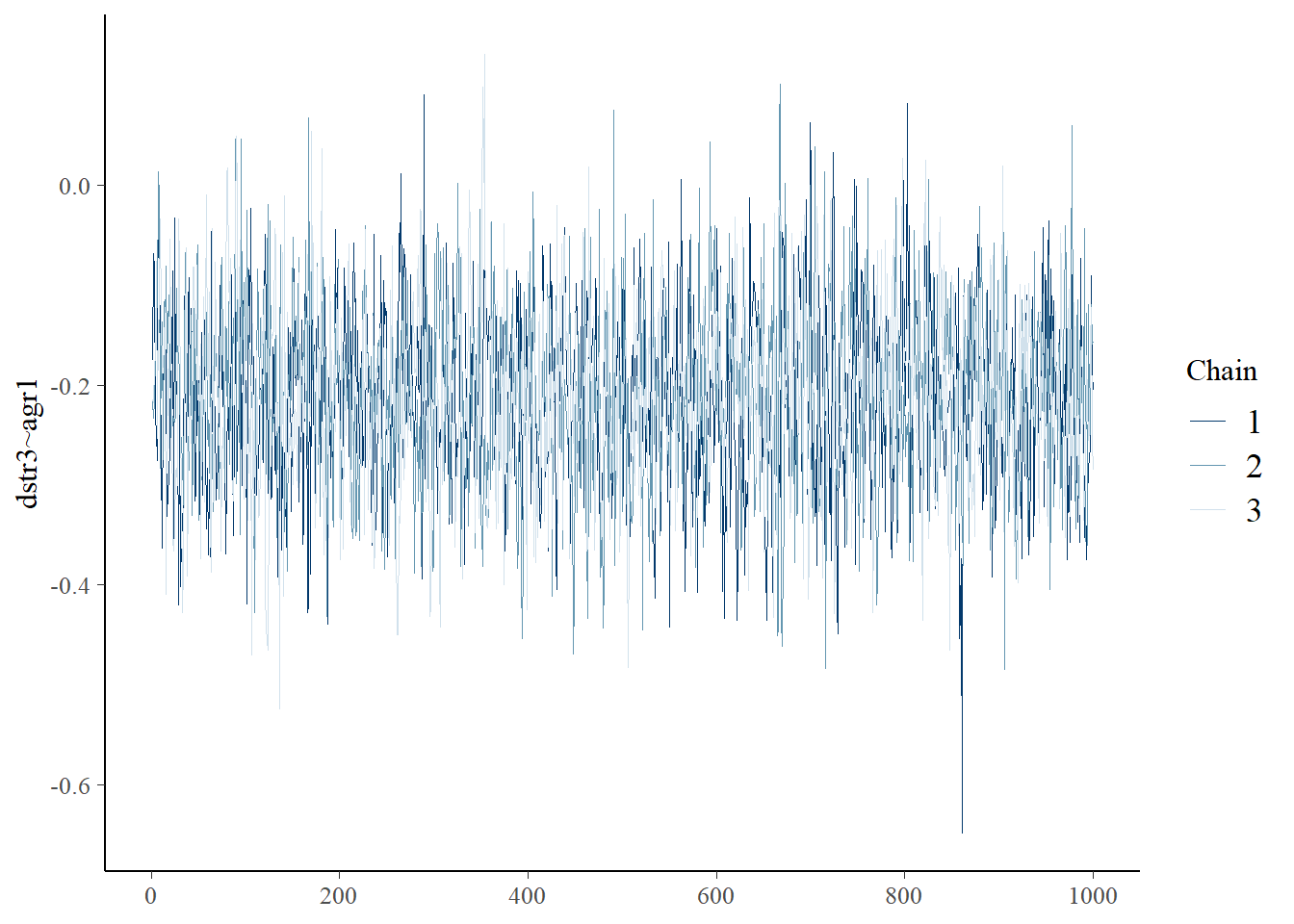


Figure 32. Traceplot of the effect of Agreeableness on change in COVID-related Stress between Wave 3 and Wave 4.

Effects of Emotional Stability
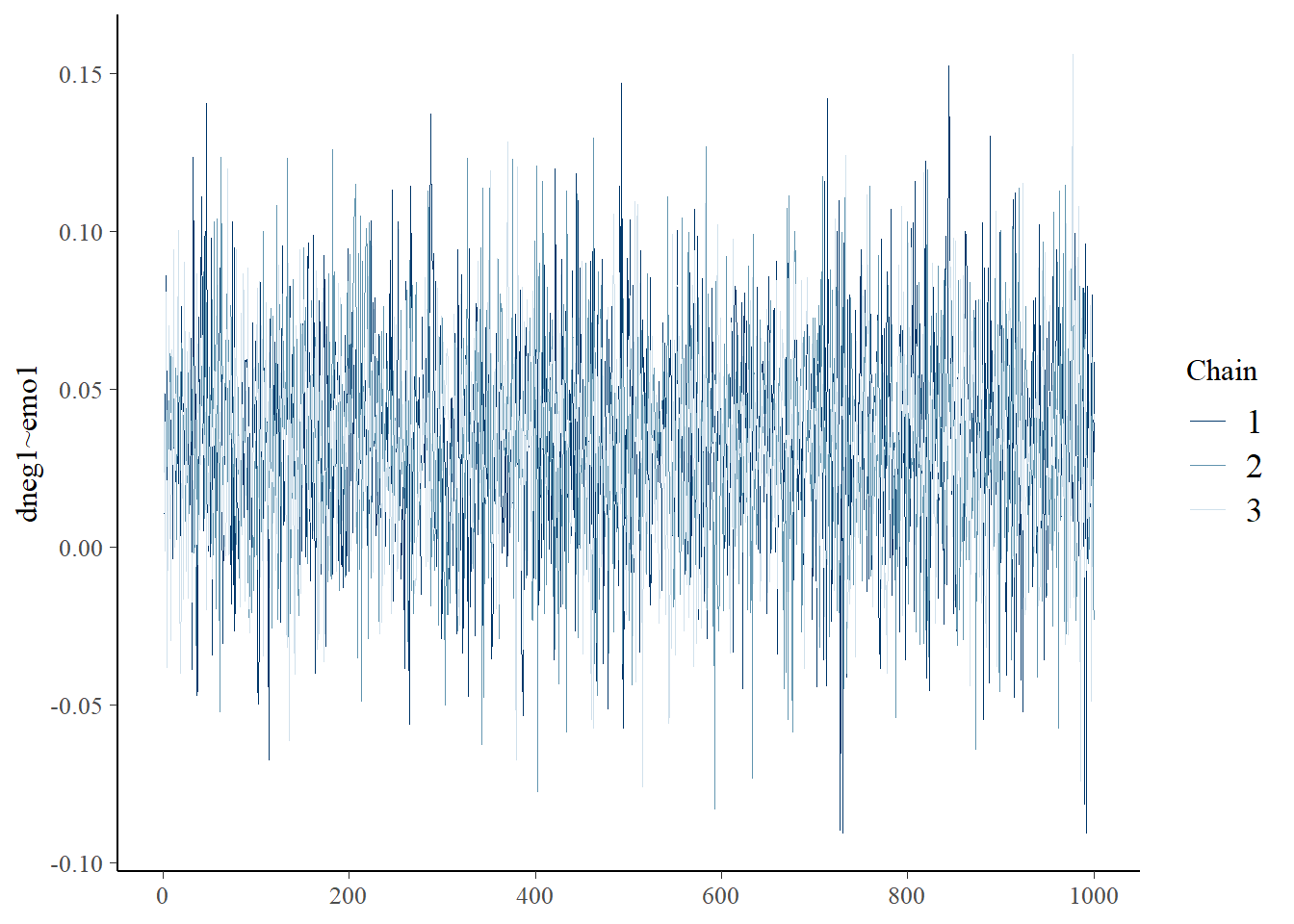


Figure 33. Traceplot of the effect of Emotional Stability on change in Negative Interactions between Wave 1 and Wave 2.


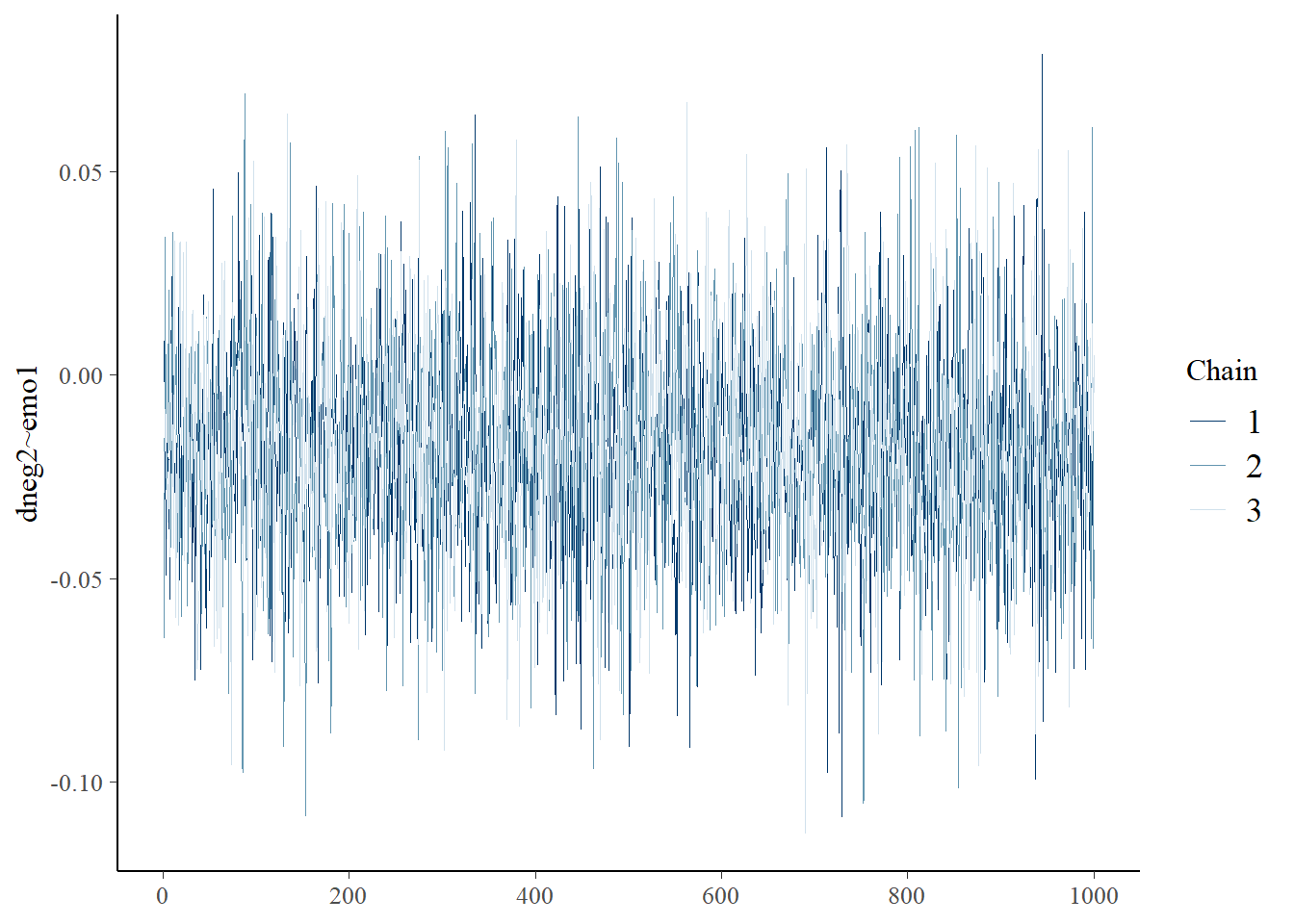


Figure 34. Traceplot of the effect of Emotional Stability on change in Negative Interactions between Wave 2 and Wave 3.


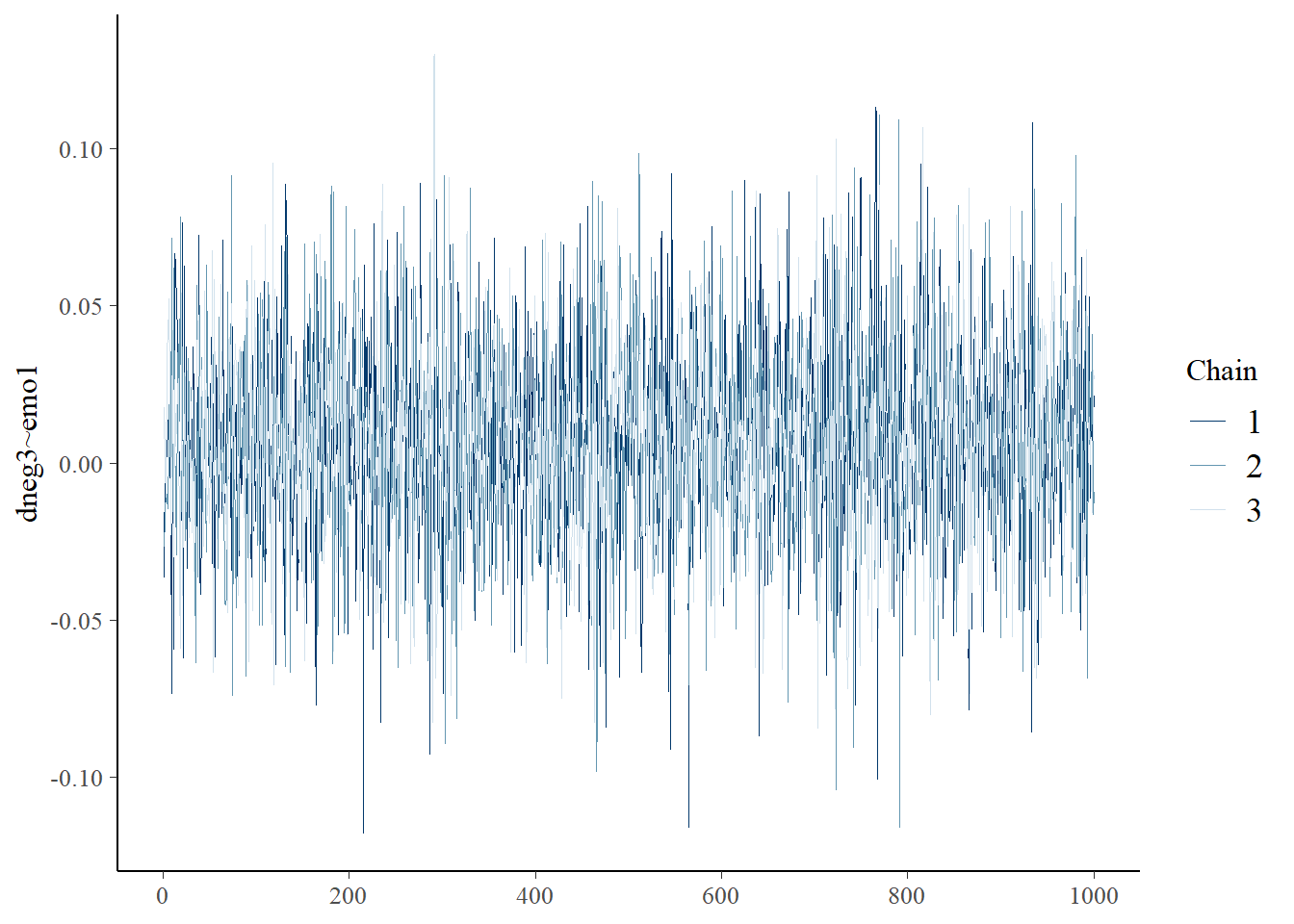


Figure 35. Traceplot of the effect of Emotional Stability on change in Negative Interactions between Wave 3 and Wave 4.


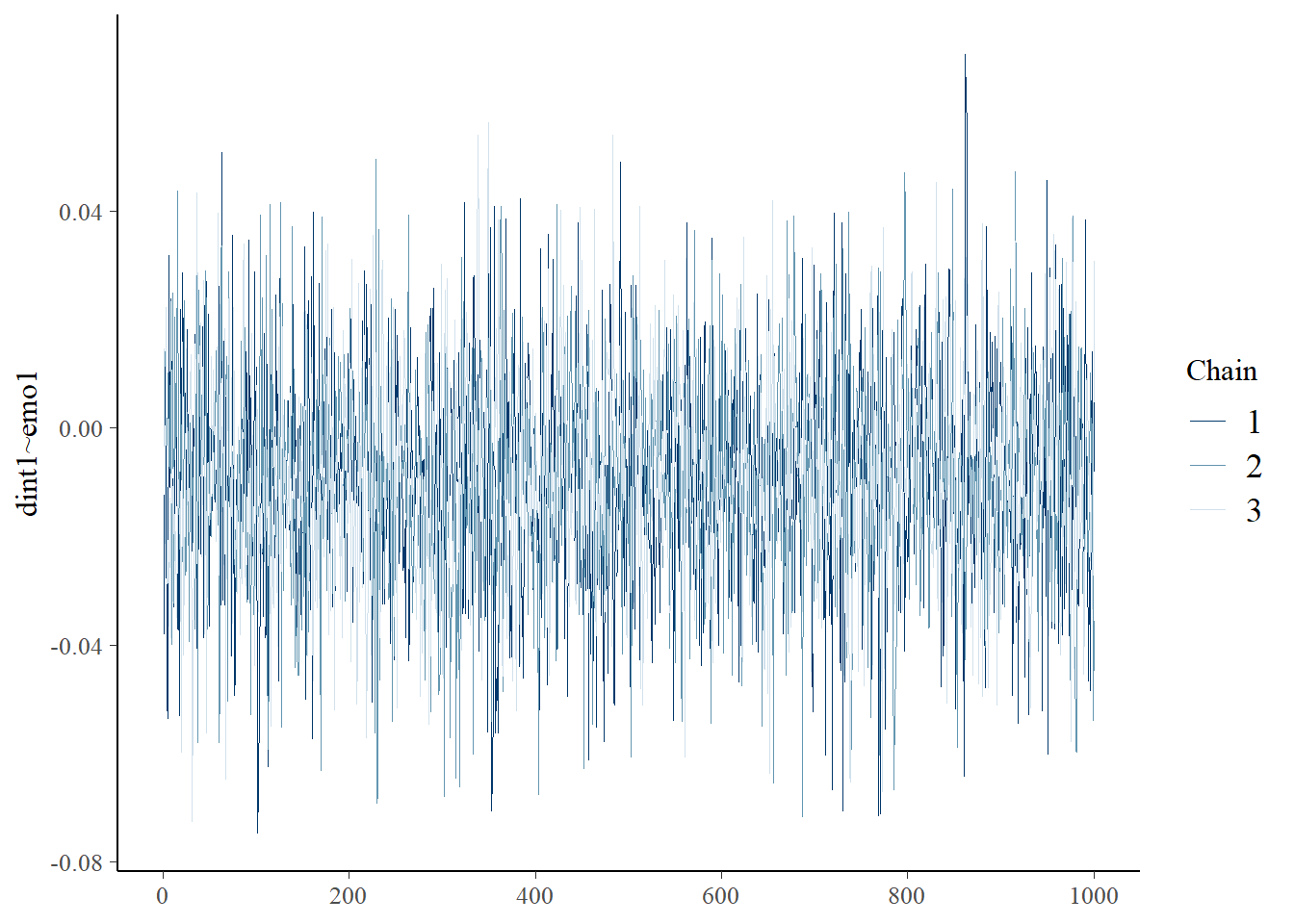


Figure 36. Traceplot of the effect of Emotional Stability on change in Mental Health Problems between Wave 1 and Wave 2.


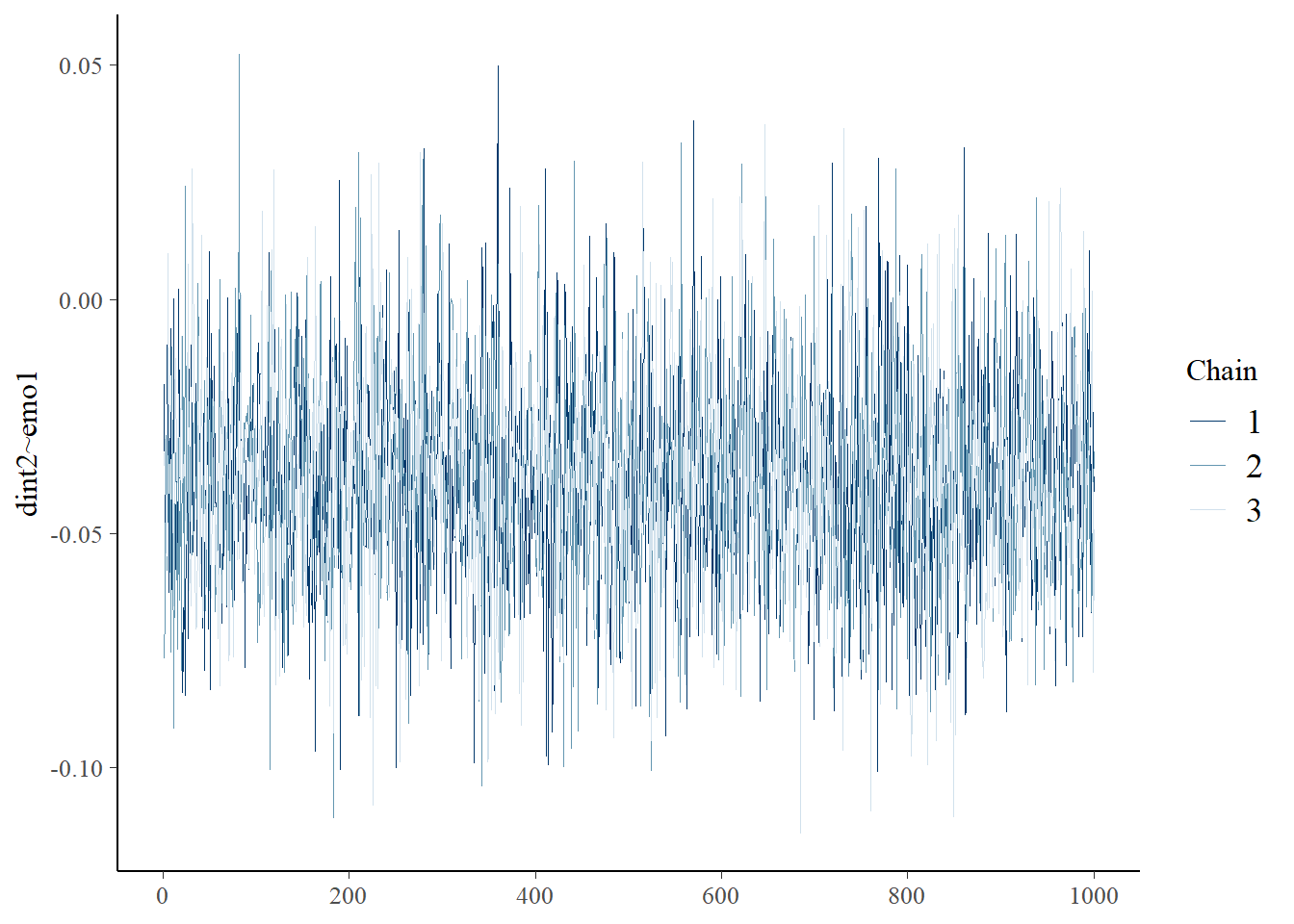


Figure 37. Traceplot of the effect of Emotional Stability on change in Mental Health Problems between Wave 2 and Wave 3.


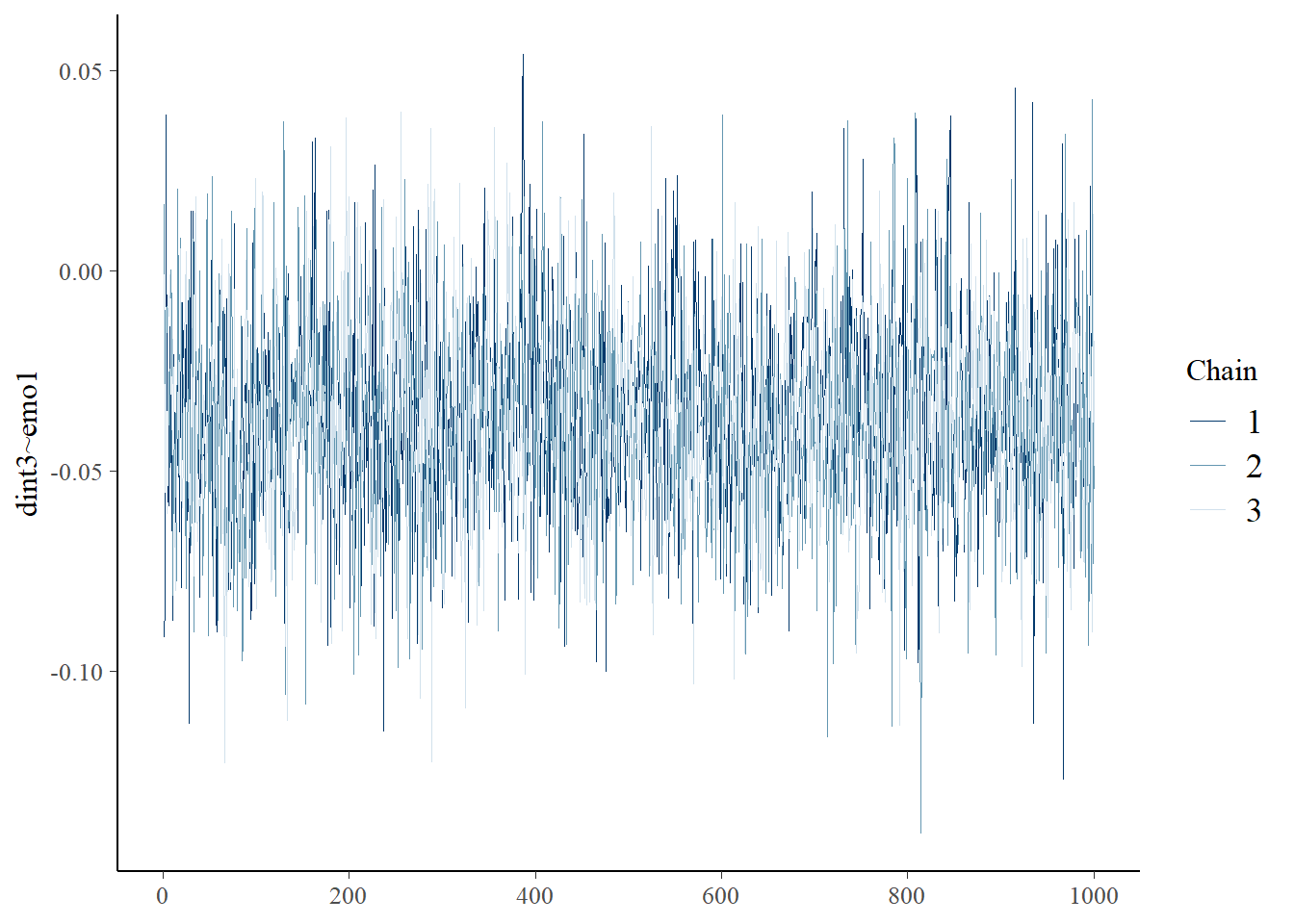


Figure 38. Traceplot of the effect of Emotional Stability on change in Mental Health Problems between Wave 3 and Wave 4.


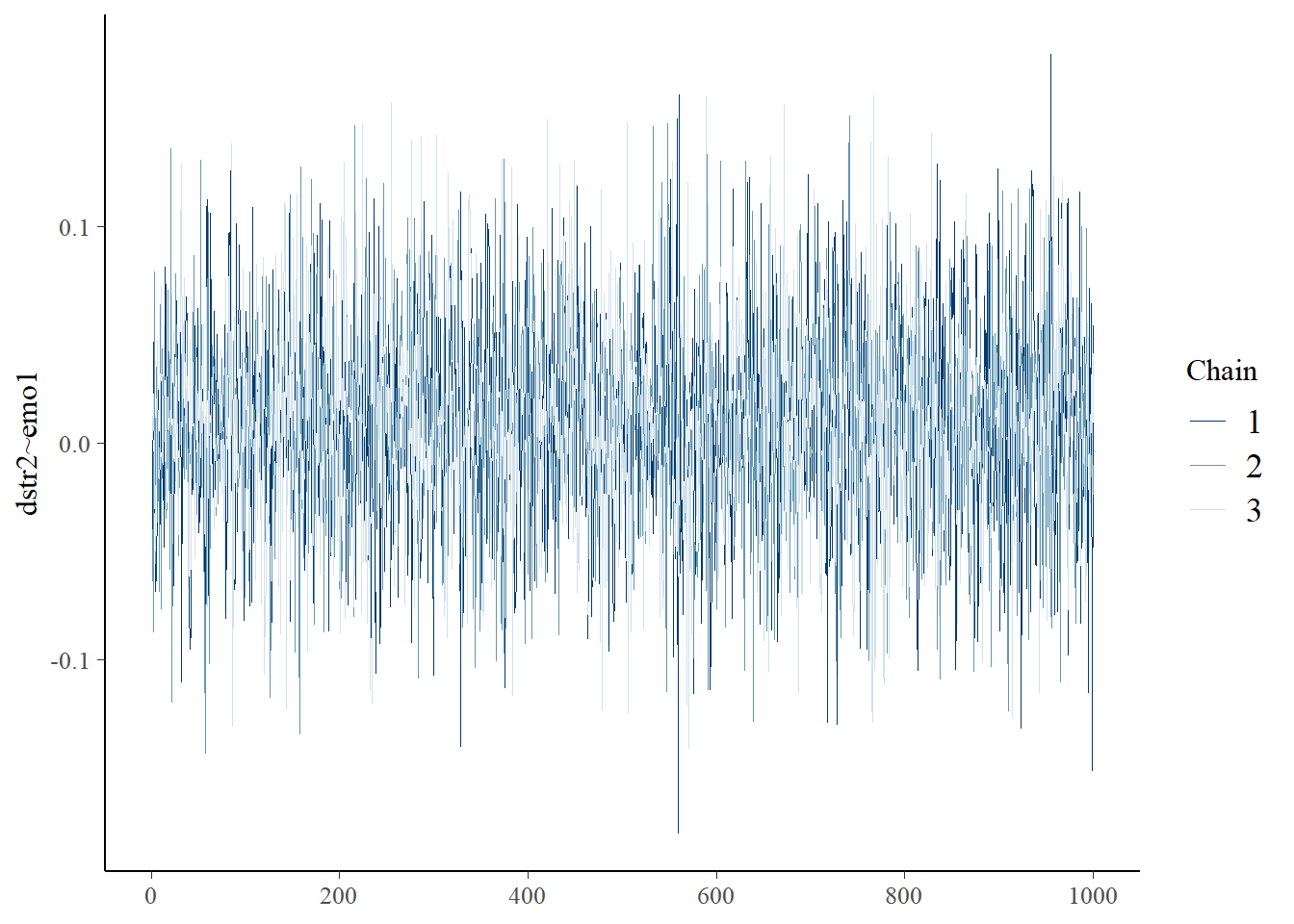


Figure 39. Traceplot of the effect of Emotional Stability on change in COVID-related Stress between Wave 2 and Wave 3.


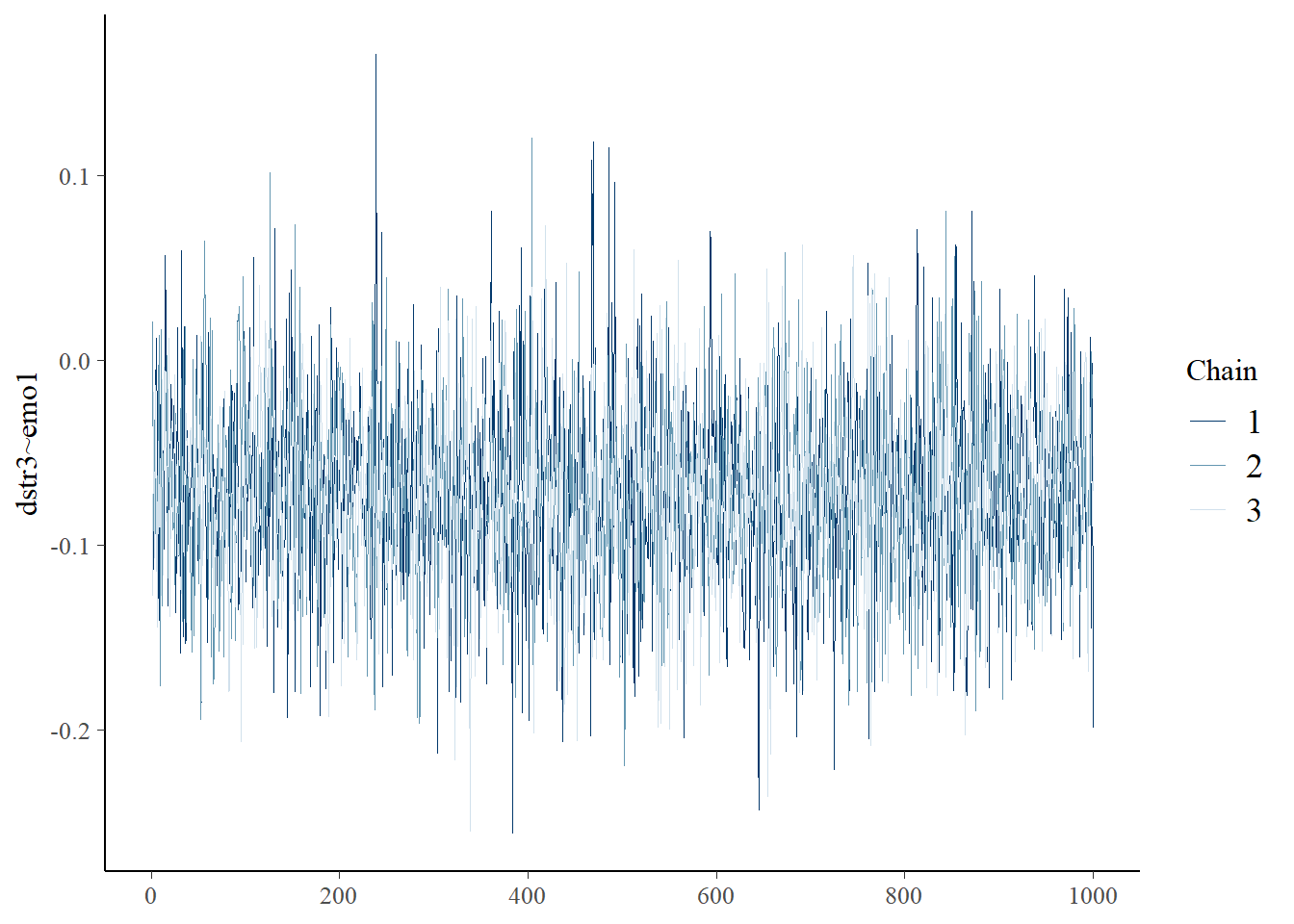


Figure 40. Traceplot of the effect of Emotional Stability on change in COVID-related Stress between Wave 3 and Wave 4.
